# PIEZO1 Ion Channel Mediates Ionizing Radiation-Induced Pulmonary Endothelial Cell Ferroptosis *via* Ca^2+^/Calpain/VE-Cadherin Signaling

**DOI:** 10.3389/fmolb.2021.725274

**Published:** 2021-09-09

**Authors:** Xue-Wei Guo, Hao Zhang, Jia-Qi Huang, Si-Nian Wang, Yan Lu, Bo Cheng, Su-He Dong, Ying-Ying Wang, Feng-Sheng Li, Yong-Wang Li

**Affiliations:** ^1^ The Postgraduate Training Base of Jinzhou Medical University (The PLA Rocket Force Characteristic Medical Center), Beijing, China; ^2^ Department of Anesthesiology, The PLA Rocket Force Characteristic Medical Center, Beijing, China; ^3^ Department of Nuclear Radiation Injury and Monitoring, The PLA Rocket Force Characteristic Medical Center, Beijing, China; ^4^ Department of Neurology, The PLA Rocket Force Characteristic Medical Center, Beijing, China; ^5^ Department of Pathology, The PLA Rocket Force Characteristic Medical Center, Beijing, China; ^6^ Department of Anesthesiology, Beijing Ditan Hospital, Capital Medical University, Beijing, China

**Keywords:** ferroptosis, Piezo1, calpain, VE-cadherin, ionizing radiation

## Abstract

Pulmonary endothelial cell dysfunction plays an important role in ionizing radiation (IR)-induced lung injury. Whether pulmonary endothelial cell ferroptosis occurs after IR and what are the underlying mechanisms remain elusive. Here, we demonstrate that 15-Gy IR induced ferroptosis characterized by lethal accumulation of reactive oxygen species (ROS), lipid peroxidation, mitochondria shrinkage, and decreased glutathione peroxidase 4 (GPX4) and SLC7A11 expression in pulmonary endothelial cells. The phenomena could be mimicked by Yoda1, a specific activator of mechanosensitive calcium channel PIEZO1. PIEZO1 protein expression was upregulated by IR *in vivo* and *in vitro*. The increased PIEZO1 expression after IR was accompanied with increased calcium influx and increased calpain activity. The effects of radiation on lung endothelial cell ferroptosis was partly reversed by inhibition of PIEZO1 activity using the selective inhibitor GsMTx4 or inhibition of downstreaming Ca^2+^/calpain signaling using PD151746. Both IR and activation of PIEZO1 led to increased degradation of VE-cadherin, while PD151746 blocked these effects. VE-cadherin knockdown by specific siRNA causes ferroptosis-like phenomena with increased ROS and lipid peroxidation in the lung endothelial cells. Overexpression of VE-cadherin partly recused the ferroptosis caused by IR or PIEZO1 activation as supported by decreased ROS production, lipid peroxidation and mitochondria shrinkage compared to IR or PIEZO1 activation alone. In summary, our study reveals a previously unrecognized role of PIEZO1 in modulating ferroptosis, providing a new target for future mitigation of radiation-induced lung injury.

## Introduction

Radiation-induced lung injury (RILI) is sometimes a critical issue in radiotherapy of thoracic malignancies (Hanania et al., 2019; Zheng et al., 2020). Preventing death of pulmonary endothelial cells is key in prevention of RILI (Guan et al., 2018). Various types of cell death including ferroptosis exist. Ferroptosis is a stress-induced iron-dependent form of cell death initiated by failure of the glutathione (GSH)-dependent antioxidant defenses, resulting in excessive reactive oxygen species (ROS) accumulation and lipid peroxidation (Bayir et al., 2020; Chen et al., 2020). Previous studies have demonstrated that ionizing radiation (IR) can induce ferroptosis in lung cancer cells, and ferroptosis inhibitor ferrostatin-1 can ameliorate cell death and restore cell function (Li X. et al., 2019; Lei et al., 2020). There is also a study showing that liproxstatin-1 reduces pathological damage related to ferroptosis in a mouse model of acute radiation-induced lung injury (Li X. et al., 2019). However, whether ionizing radiation can induce pulmonary endothelial cell ferroptosis remains obscure.

PIEZO1, a mechanically activated calcium (Ca^2+^)-permeable ion channel (Coste et al. 2010), is highly expressed in lung tissues (Zhao et al., 2018; Solis et al., 2019). Previous studies established that PIEZO1/Ca^2+^ signaling mediates release of flow-induced ATP and subsequent destruction of pulmonary endothelial barrier in pulmonary endothelial cells (Wang et al. 2016; Friedrich et al., 2019). More research evidences aver that PIEZO1 is a critical regulator of iron metabolism (Andolfo et al., 2020; Hanchard and Wonkam, 2021; Ma et al., 2021). Furthermore, activation of PIEZO1 can increase the levels of ROS and the concentration of Ca^2+^ ions in cardiomyocytes and nucleus pulposus cells (Jiang F. et al., 2021; Wang et al., 2021). Iron overload and ROS excess are closely related with ferroptosis. Therefore, PIEZO1/Ca^2+^ signaling possibly regulates ferroptosis of pulmonary endothelial cells as well.

PIEZO1 acts mainly through calcium signaling. Activation of PIEZO1 leads to increased influx of Ca^2+^, which activates calpain, a calcium-dependent cysteine proteases involved in degradation of several proteins. Vascular endothelial-cadherin (VE-cadherin) is a calcium-dependent adhesive molecule exclusively expressed in endothelial cells (Gory et al., 1999). Homophilic adhesion of VE-cadherin constitutes adherent junctions (Vandenbroucke St Amant et al. 2012), which help in maintaining vascular integrity and stability (Chen et al., 2012). Calpain plays essential roles in mediating internalization and degradation of VE-cadherin (Tiruppathi et al., 2002; Su and Kowalczyk, 2017; Bhatia et al., 2021). Previous studies reported that calpain/VE-cadherin pathway is responsible for PIEZO1-mediated disruption of the pulmonary endothelial barrier (Friedrich et al., 2019). Increased degradation of VE-cadherin has also been found to cause cerebrovascular dysfunction (Bhatia et al. 2021), ventilation-induced lung vascular hyperpermeability (Jiang L. et al. 2021) and lung edema (Cowan et al., 2010). Previous studies have recently established that increase in VE-cadherin internalization occurred in erastin-induced endothelial cell ferroptosis (Stockwell et al., 2017; Lopes-Coelho et al., 2021). E-cadherin, analog of VE-cadherin, regulates ferroptosis (Wu et al., 2019a). Therefore, the current study explored the role of PIEZO1 in regulation of pulmonary endothelial cell ferroptosis through calpain/VE-cadherin pathway.

## Materials and Methods

### Cell Culture

Human pulmonary microvascular endothelial cells (HULEC-5a) were purchased from Qincheng Biological Company (Shanghai, China) and were cultured in DMEM (Gibco) medium containing 10% fetal bovine serum (FBS), 2 mM l-glutamine, 100 U/ml penicillin, and 100 mg/ml streptomycin at 37°C with 5% CO_2_. Medium was changed every day, and cells were passaged when confluency reached around 80%. Cells used in the current study underwent 6–18 passages.

### Mice

Adult C57BL/6 mice were purchased from the SPF (Beijing) Biotechnology Co., Ltd. and kept in animal room at the PLA Rocket Force Characteristic Medical Center. Mice were anesthetized with pentobarbital sodium (40 mg/kg body weight), and were subjected to 15-Gy ionizing radiation (IR) on the whole thorax under general anesthesia. Other animal parts outside chest were covered with 5 mm thick lead block. Three mice were assigned to each group. All experimental procedures were performed according to Guide for Care and Use of Laboratory Animals (8^th^ edition, National Academies Press, Washington, DC, 2011) and were approved by local Ethics Review Board.

### Cell Transfection

VE-cadherin scrambled siRNA (si-VE-cadherin) for human VE-cadherin was synthesized and verified by GenePharma (GenePharma, Shanghai, China). Cells with about 80% confluency were used for transfection. siRNA and negative control were transfected using Lipofectamine 3,000 (Thermo Fisher Scientific, Inc. Waltham, MA, United States). 2.5 μg/ml of siRNA or equal amount of negative control was used. After 48-hour transfection, cells were eluted and used for further experiment. Efficiency of VE-cadherin silencing in cells was verified using Western blot and reverse transcription quantitative real-time PCR (RT-qPCR). Previously validated siRNA sequence targeting VE-cadherin (5′-AAC​CAG​AUG​CAC​AUU​GAU​UTT-3′, 5′-AAU​CAA​UGU​GCA​UCU​GGU​UCC-3′ (Li R. et al. 2019)) was used. The sequence of negative control were 5′-UUC​UCC​GAA​CGU​GUC​ACG​UTT-3′, 5′-ACG​UGA​CAC​GUU​CGG​AGA​ATT-3′.

The plasmid for overexpressing human VE-cadherin (NM_001795.5, OE-VE-cadherin) was constructed and synthesized by Hanbio (Hanbio, shanghai, China). The study used the pHBLV-CMV-MCS-3FLAG-EF1-PURO plasmid. Lipofectamine 3,000 (Thermo Fisher Scientific, Inc. Waltham, MA, United States) was used for transfection. Transfection concentration for both overexpression VE-cadherin and negative control groups was 2.5 μg/ml. Cells were collected at 24 h post-transfection. The transfection efficiency was verified by RT-qRCR and Western blot.

### RT-qPCR

Total RNA was extracted from HULEC-5a cells with TRIzol (Sigma, United States). Total RNA was then reverse-transcribed into cDNA using PrimeScript™ RT reagent kit with gDNA Eraser (Takara, Japan). cDNA solution was amplified on PCR instrument (Bio-Rad) following two steps of pre-denaturation and PCR reaction. Findings were subsequently analyzed based on Bio-Rad CFX Manager software using 2^−ΔΔCt^ method with GAPDH as internal control. The sequences of primers used were as follows: VE-cadherin, forward: CCT​CTG​TGG​GCT​CTC​TGT​TTG​TTG and reverse: TGT​CTC​AAT​GGT​GAA​AGC​GTC​CTG. GAPDH, forward: GCC​ATC​ACT​GCC​ACT​CAG​AA and reverse: GGC​ATG​TCA​GAT​CCA​CAA​CG.

### Immunohistochemistry

Lung tissues of mice at 24 h following IR were fixed in 4% paraformaldehyde fixative solution, and then sliced at thickness of 5 µm after embedding in paraffin. Paraffin sections of lung tissues were deparaffinized and antigen retrieval was then undertaken using Tris-EDTA Buffer (pH 9.0) at 100°C for 30 min. Endogenous peroxidase was then blocked using 0.3% hydrogen peroxide methanol solution. Sections were incubated overnight with rabbit primary antibodies, which included anti-PIEZO1 (Abcam, ab128245, 1:50), anti-GPX4 (Abcam, ab125066, 1:100), anti-VE-cadherin (Cell Signaling Technology, #2500,1:50), and anti-SCL7A11 (Abcam, ab37185, 1:100). Horseradish peroxidase-conjugated goat anti-rabbit IgG was used as the secondary antibody. Sections were dehydrated and fixed after counterstaining with hematoxylin. Sections were chemically developed based on the DAB method, and then observed and photographed under microscope.

### Fluorescent Ca^2+^ Imaging

Previous studies aver that Fluo4-AM detects cellular Ca^2+^ ion concentration (Murphy et al., 2008). HULEC-5a cells were plated in small confocal dish in advance. HULEC-5a cells were then incubated with Fluo4-AM at a working concentration of 5 µM for 30 min at 37°C. Cell were washed with calcium-free and magnesium-free balanced salt solution, and then incubated with balanced salt solution at 37°C for 20 min. Fluo4-AM fluorescence signal was then detected using fluorescence microscope at excitation wavelength of 494 nm and emission wavelength of 516 nm. Ca^2+^ concentration in images were analyzed based on average fluorescence intensity using the ImageJ software.

### Western Blot Analysis

Protein was obtained from cells using RIPA buffer (Thermo, TL281708) supplemented with protease and phosphatase cocktails (NCM biotech, P002). Equal amounts of proteins were separated by 10% SDS-PAGE and transferred to polyvinylidene fluoride membranes (Bio-Rad, United States). Membrane was blocked in 5% non-fat milk for 1 h at room temperature and then incubated with primary antibodies overnight at 4 °C. Primary antibodies that were used included rabbit anti-GPX4 (Abcam, ab125066, 1:1,000), rabbit anti-VE-cadherin (CST, #2500,1:1,000), rabbit anti-DMT1 (Cell Signaling Technology, #15083.1:1,000), rabbit anti-SLC7A11 (Abcam, ab37185, 1:1,000), mouse anti-PIEZO1 (Novus Biologcals, NBP2-75617, 1:1,000), rabbit anti-ACSL4 (abcam, ab155282, 1:5,000), mouse anti-α-tubulin (Proteintech, 66,031–1,1:5,000) and mouse anti-GAPDH (Proteintech, 60,004–1,1:1,000). Proteins were incubated on the following day with horseradish peroxidase-conjugated secondary antibody for 1 h at room temperature. Chemiluminescence was captured on gel imaging system (Bio-Rad ChemiDoc MP). Protein expression was analyzed by computing gray value using ImageJ software, with GAPDH or α-tubulin as the internal control.

### Transmission Electron Microscopy

Cells were attached to small confocal dish in advance. They were rinsed with 0.1 M sodium cacodylate buffer (pH 7.2), and a mixture of 2.5% electron microscope grade glutaraldehyde fixing solution and 2% paraformaldehyde were added to fix cells at room temperature for 30 min followed by overnight incubation at 4°C. Samples were washed with 0.1 M sodium cacodylate buffer and treated with 0.1% Millipore-filtered cacodylate-buffered tannic acid, postfixed with 1% buffered osmium and stained *en bloc* using 1% Millipore-filtered uranyl acetate. Samples were dehydrated using ethanol, permeated and embedded in LX-112 medium. Samples were then polymerized in 60°C oven for about 3 days. Ultrathin sections were cut using Leica Ultracut Microtome, stained with uranyl acetate and citrate in Leica EM STAPRAR, and examined using JEM-2100F transmission electron microscope (Chinese Academy of Sciences, Beijing). Digital images were obtained using AMT Imaging System.

### Lipid Peroxidation Assay

Lipid peroxidation levels were determined as previously described (Zhang et al., 2018; Lei et al., 2020). 5 μM C11-BODIPY 581/591(GLPBIO, GC40165) were added to cell medium 24 h following irradiation. Cells were further incubated for 30 min and then were washed using PBS, digested with 2.5% trypsin and neutralized with 10% FBS in PBS at 1:1 volume. Supernatants were discarded and 400 µL of Hank's Balanced Salt Solution (HBSS) were added to resuspend cells. Lipid peroxidation levels were then measured using flow cytometer at 495/529 nm.

### ROS Measurement

Levels of ROS in HULEC-5a cells were determined using ROS determination kit (Gene Copoeia, A507) based on manufacturer’s instructions. Briefly, treated HULEC-5a cells were washed twice with calcium and magnesium-free balanced salt solution. Cells were treated with working concentration of 10 µM H_2_DCFDA for 30 min at 37°C under light-proof conditions. Cells were digested with trypsin and resuspended with balanced salt solution. Cellular ROS production was determined using flow cytometer at 495/529 nm.

### Calpain Activity

Calpain activity was determined using calpain activity assay kit (Abcam, ab65308). Briefly, HULEC-5a cells were collected and homogenized in supplied extraction buffer. Protein concentration was determined using BCA method and standardized. Samples were mixed with reaction buffer and calpain, and incubated in darkness at 37°C for 60 min. Fluorescence value of each sample was determined using excitation at 400 nm and emission at 505 nm. Calpain activities of samples were computed after normalizing negative and positive data.

### Statistical Analysis

GraphPad Prism 7 (GraphPad Software, San Diego, CA, United States) was used for data analysis. All experiments were repeated at least thrice, and numerical data was presented as mean ± standard error (SEM). One-way Analysis of Variance (ANOVA) with post hoc Tukey test was used to compare data between and among groups. A *p* value of smaller than 0.05 was considered statistically significant.

## Results

### Ionizing Radiation Induces Pulmonary Endothelial Cell Ferroptosis and Increases Expression of PIEZO1 Protein

**FIGURE 1:**
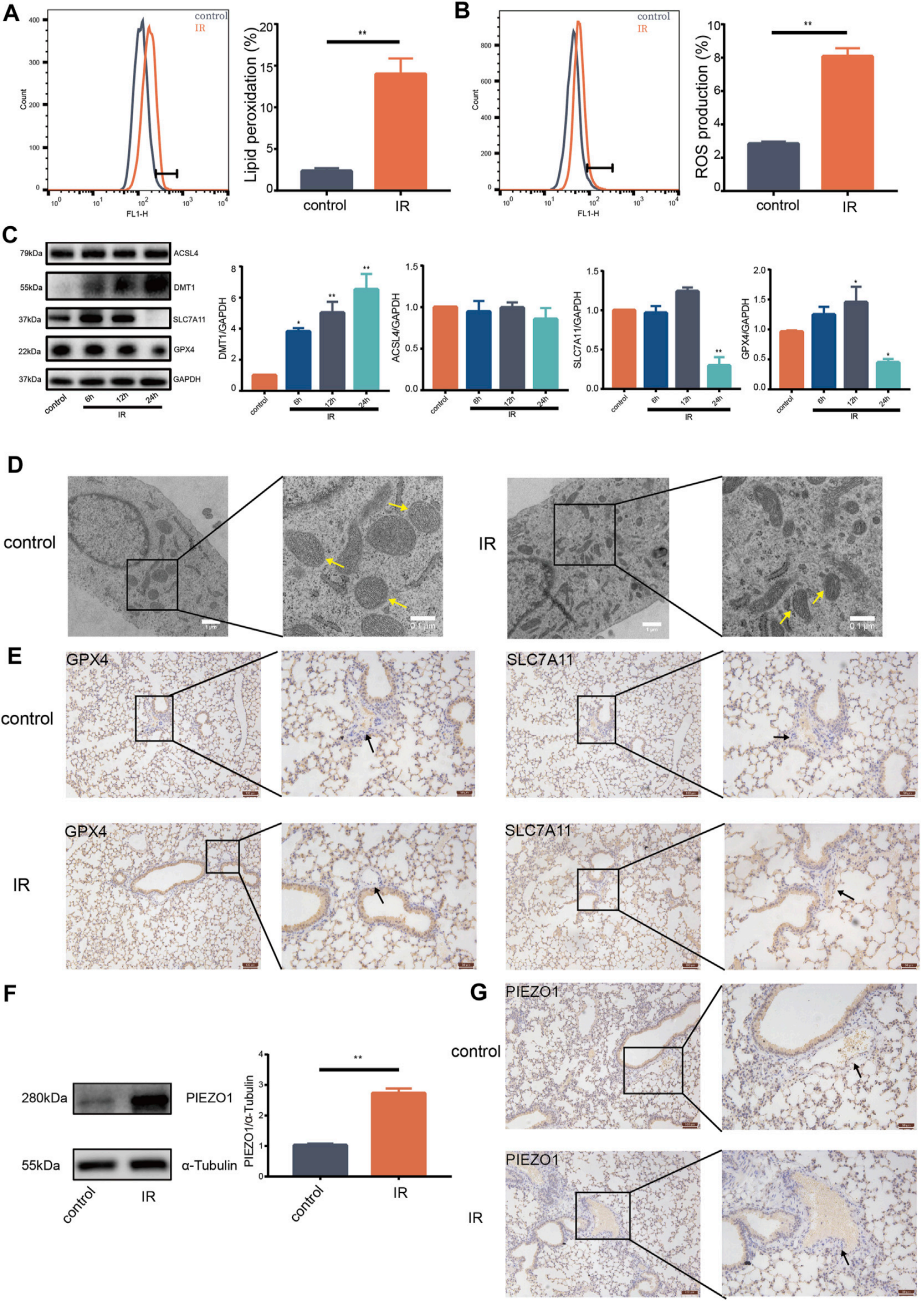
Ionizing radiation induced induces pulmonary endothelial cell ferroptosis and increases expression of PIEZO1 protein. **(A)** Lipid peroxidation assessment in HULEC-5a cells, 24 h after exposure to 15-Gy of ionizing radiation (IR). Bar graphs showed relative levels of lipid peroxidation by C11-BODIPY staining in indicated cells. **(B)** ROS measurement in HULEC-5a cells, 24 h after IR. ROS levels were determined using H_2_DCFDA and analyzed using flow cytometry. **(C)** Western blotting analysis of ACSL4, SLC7A11, DMT1, and GPX4 protein expression in HULEC-5a cells, 6, 12, and 24 h after IR. **(D)** Representative transmission electron microscopy images of HULEC-5a cells after IR. Yellow arrows show mitochondria. **(E)** Expression of GPX4 and SLC7A11 detected by immunohistochemistry assay in lungs of mice after IR. Scale bars: 100 µm (left) and 50 µm (right). **(F)** Western blot analysis of PIEZO1 protein expression in HULEC-5a cells after IR. **(G)** Expression of PIEZO1 protein detected by immunohistochemistry assay. Scale bars: 100 µm (left) and 50 µm (right). Data were plotted as means ± SEM. *n* = 3 independent repeats. **, *p* < 0.01 *vs* control.

Ionizing radiation (IR) can cause death of vascular endothelial cells in short time, leading to radiation damage (Guan et al., 2018). Previous studies reported that increase of ROS and accumulation of lipid peroxides are biological hallmarks of ferroptosis (Stockwell et al., 2017; Kazan and Kalaipandian, 2019). The current study found that IR induces increase of lipid peroxides (Figure 1A) and accumulation of ROS in lung endothelial cells (Figure 1B). Moreover, expression of GPX4 and SLC7A11 protein was decreased (Figure 1C). Expression of DMT1 protein was increased compared with expression in control group (Figure 1C). Notably, there was no significant change in the expression of ACSL4 protein (Figure 1C). HULEC-5a cells had shrunken mitochondria and increased mitochondrial membrane density after 24 h following IR as shown by transmission electron microscopy (Figure 1D). Expression of GPX4 and SLC7A11 proteins on the pulmonary vascular endothelial cells was reduced 24 h after IR in mice (Figure 1E). These results support that pulmonary endothelial cell undergo ferroptosis after IR. Western blot findings showed that IR induces an increase in the expression of PIEZO1 protein in HULEC-5a cells (Figure 1F). We also observed that the expression of PIEZO1 protein in the lung epithelial cells of mice was increased 24 h after IR compared with the expression in control mice (Figure 1G). These results provide a potential link between PIEZO1 and radiation-induced ferroptosis of lung endothelium.

### PIEZO1 Mediates Pulmonary Endothelial Cell Ferroptosis Induced by Ionizing Radiation

**FIGURE 2:**
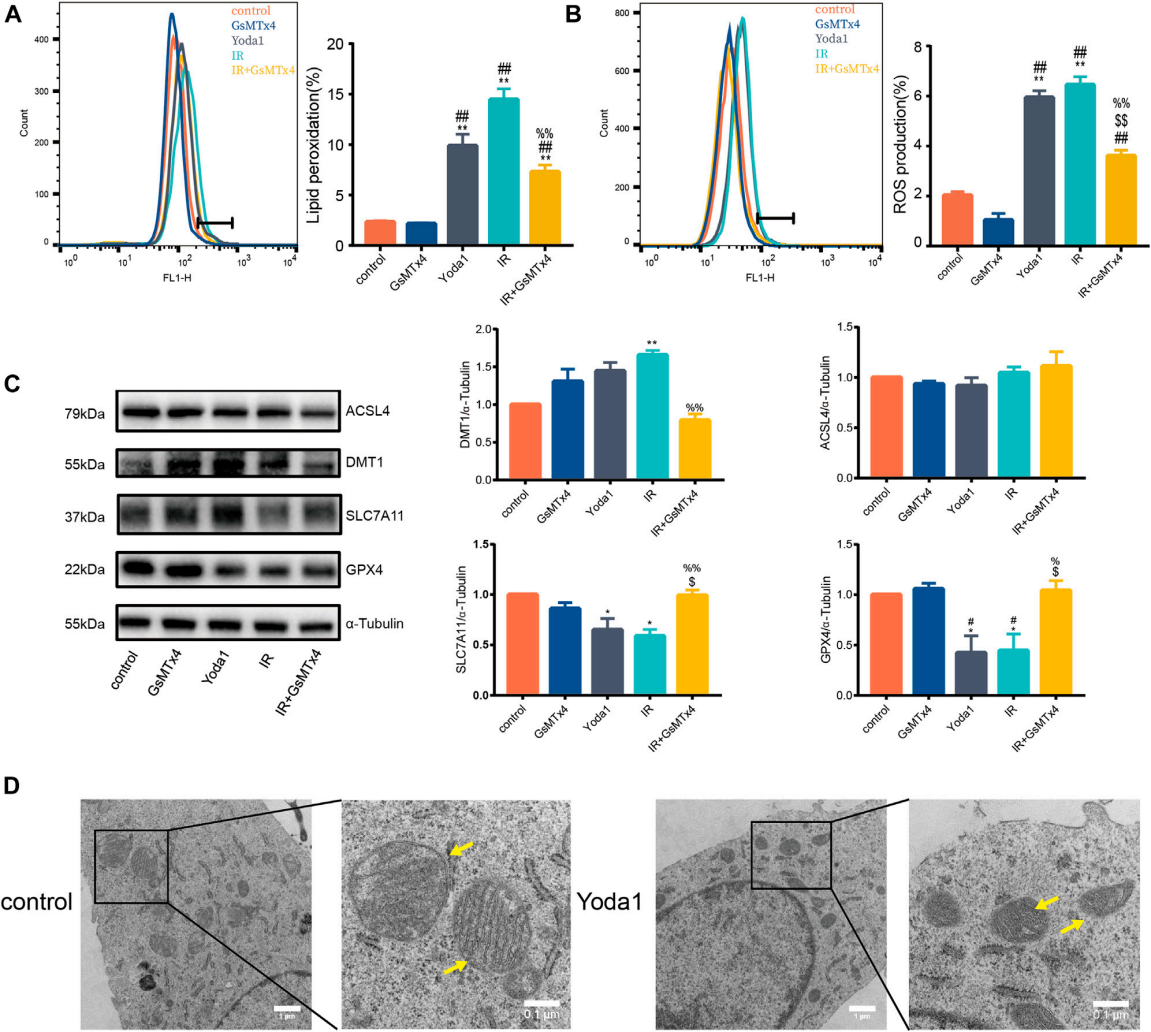
PIEZO1 mediates pulmonary endothelial cell ferroptosis induced by ionizing radiation. **(A)** Lipid peroxidation assessment in HULEC-5a cells pre-treated with Yoda1 (2.5 µM) or GsMTx4 (5 µM) before ionizing radiation (IR). **(B)** ROS measurement in HULEC-5a cell pre-treated with Yoda1 (2.5 µM) or GsMTx4 (5 µM) 30 min before IR until the end of the experiment (24 h post IR). **(C)** Western blotting analysis of ACSL4, SLC7A11, DMT1, and GPX4 protein expression in HULEC-5a cell pre-treated with Yoda1 (2.5 µM) or GsMTx4 (5 µM) 30 min before IR. **(D)** Transmission electron microscopy images of HULEC-5a cells without radiation (control) or at 24 h after Yoda1 treatment. Yellow arrows show mitochondria. Data were plotted as means ± SEM. n = 3 independent repeats. **, *p* < 0.01 *vs* control; ^##^, *p* < 0.01 *vs* GsMTx4; ^$$^, *p* < 0.01 *vs* Yoda1; ^%%^, *p* < 0.01 *vs* IR.

The current study further explore the functional role of increased PIEZO1 protein expression following IR. Finding showed that Yoda1, a specific activator of PIEZO1, induced accumulation of lipid peroxides (Figure 2A) and ROS (Figure 2B) in HULEC-5a cells, which were consistent to the effects of IR. Expression of GPX4 and SLC7A11 proteins were significantly reduced, and expression of DMT1 was increased in Yoda1 group compared with those in control group, respectively (Figure 2C). In converse, GsMTx4, a specific inhibitor of PIEZO1, partially attenuates increase in ROS (Figure 2B), lipid peroxidation (Figure 2A) and the ferroptosis-related protein expression changes caused by IR exposure (Figure 2C). Transmission electron microscopy imaging revealed that HULEC-5a cells receiving Yoda1 (5 µM) treatment exhibited increased mitochondrial membrane density and decreased mitochondrial cristae density, which are typical morphologic features of ferroptosis (Figure 2D).

### PIEZO1 Mediates IR-Induced Ferroptosis by Increasing Intracellular Calcium Concentration and Calpain Activity

**FIGURE 3:**
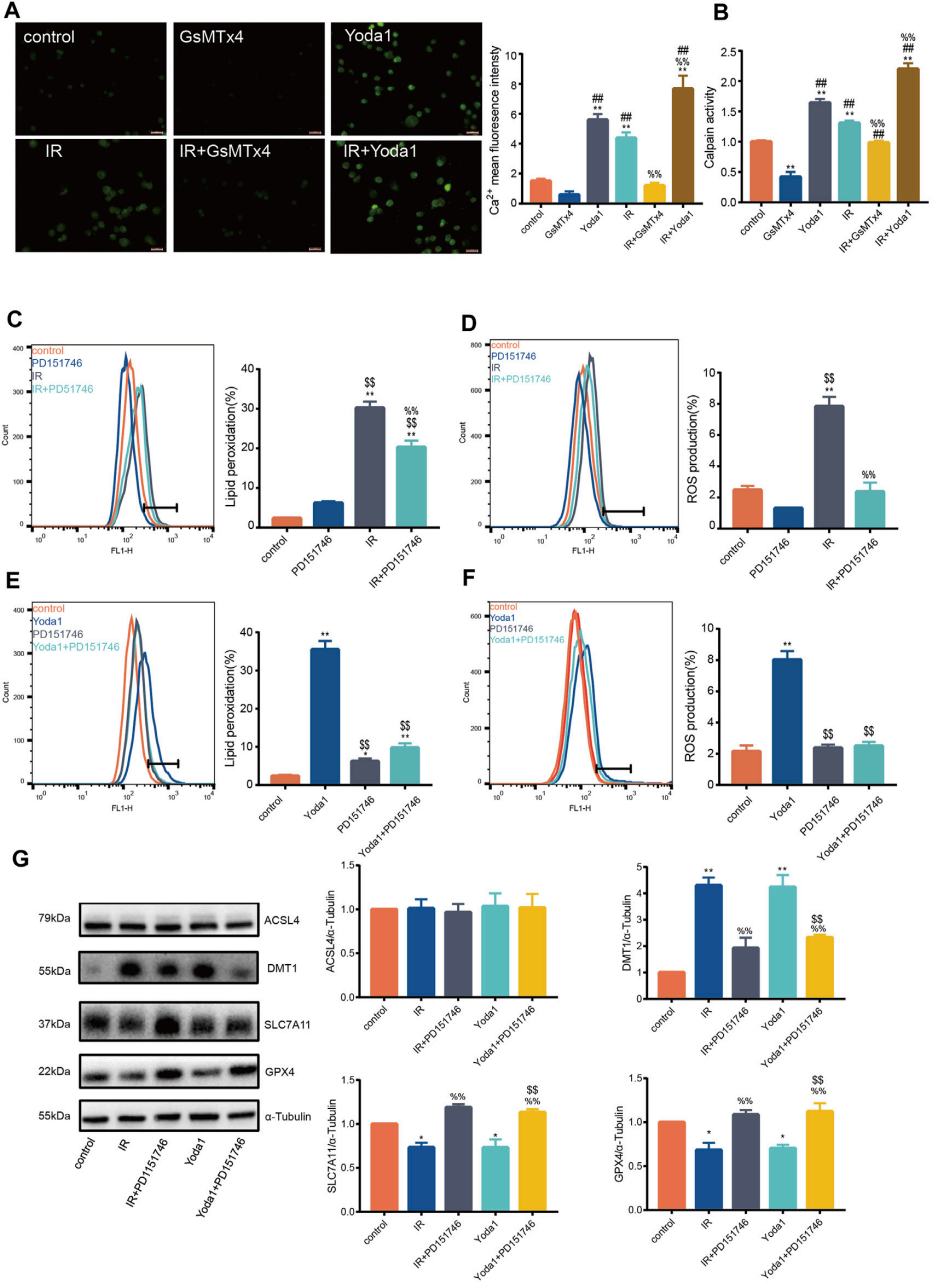
PIEZO1 mediates ionizing radiation-induced pulmonary endothelial cell ferroptosis by increasing intracellular calcium concentration and calpain activity. **(A)** Intracellular Ca^2+^ measured using Fluo4-AM (5 mM) across groups. Histograms showing differences in Ca^2+^ concentrations across groups. **(B)** Calpain activity assay in HULEC-5a cell pre-treated with Yoda1 (2.5 µM) or GsMTx4 (5 µM) for 24 h before ionizing radiation (IR). **(C)** Lipid peroxidation assessment in HULEC-5a cells pre-treated with PD151746 (20 µM) for 24 h before IR. **(D)** ROS measurement in HULEC-5a cell pre-treated with PD151746 (20 µM) for 24 h before IR or without radiation. **(E)** Lipid peroxidation assessment in HULEC-5a cells pre-treated with PD151746 (20 µM) and/or Yoda1 for 24 h. The Lipid peroxidation level of the control group is the same result shown in Figure 3C. The grouping experiment belongs to single batch of experiment. **(F)** ROS measurement in HULEC-5a cells pre-treated with PD151746 (20 µM) and/or Yoda1 for 24 h. **(G)** Western blotting analysis of ACSL4, SLC7A11, DMT1, and GPX4 expression in HULEC-5a cell pretreated with PD151746 (20 µM) for 24 h followed by IR and/or treatment with Yoda1. Data were plotted as means ± SEM. n = 3 independent repeats. **, *p* < 0.01 *vs* control. ^&&^, *p* < 0.01 *vs* PD151746. ^##^, *p* < 0.01 *vs* GsMTx4. ^$$^, *p* < 0.01 *vs* Yoda1; ^%%^, *p* < 0.01 *vs* IR.

Previous studies aver that PIEZO1 modulates influx of intracellular calcium, whose increase activates calpain signaling (Li et al., 2014). Findings of the current study established that calcium concentration after IR or Yoda1 treatment increased significantly compared with that of control (Figure 3A). Calcium concentration after concurrent IR and Yoda1 treatment was even higher compared with ionizing radiation or Yoda1 treatment alone. GsMTx4 partly blocked elevated calcium concentration caused by IR (Figure 3A). The current study also showed significantly increased calpain activity after IR in HULEC-5a cells, whereas this response was blocked when treated with GsMTx4, a selective PIEZO1 antagonist (Figure 3B).

Incubation of irradiated HULEC-5a cells with selective calpain inhibitor, PD151746, led to decreased calpain activity accompanied by decreased lipid peroxidation (Figure 3C) and ROS quantity (Figure 3D). Same effects of PD151746 on lipid peroxidation and ROS were observed when PD151746 was co-incubated with Yoda1 (Figures 3E, F). Furthermore, PD151746 partly reversed decrease in GPX4 and SLC7A11 protein expression and increase in DMT1 protein expression caused by IR or PIEZO1 activation (Figure 3G).

### Increased VE-Cadherin Degradation Contributed to the Ferroptosis-Inducing Effect of Ionizing Radiation and PIEZO1 Activation

**FIGURE 4:**
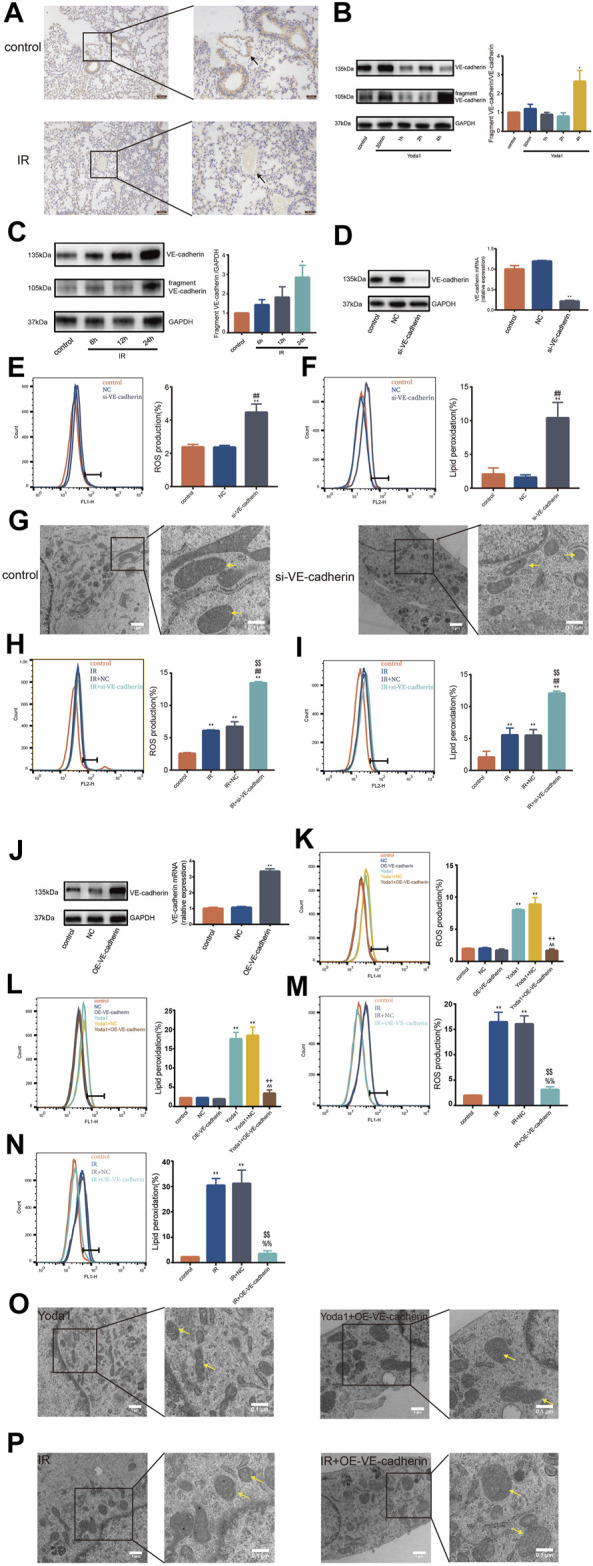
VE-cadherin contributes to the ferroptosis-inducing effect of PIEZO1 **(A)** Expression of VE-cadherin detected by immunohistochemistry assay in lungs of mice 24 h after exposure to 15-Gy of ionizing radiation (IR) or no radiation. Scale bars: 100 µm (left) and 50 µm (right) **(B)** Western blotting analysis of VE-cadherin and VE-cadherin fragment in HULEC-5a cell at 30 min, 1, 2 and 4 h after treatment with Yoda1. **(C)** Western blotting analysis of VE-cadherin and VE-cadherin fragment expression in HULEC-5a cell at 6, 12, and 24 h after exposure to IR or no radiation. **(D)** Western blot and RT-PCR analyses showing successful knockdown of VE-cadherin in HULEC-5a cells. (**E**) ROS measurement in HULEC-5a cells with knockdown of VE-cadherin. **(F)** Lipid peroxidation assessment in HULEC-5a cells with knockdown of VE-cadherin. **(G)** Transmission electron microscopy images of HULEC-5a cells with or without knockdown of VE-cadherin. **(H)** ROS measurement in HULEC-5a cells with knockdown of VE-cadherin subjected to IR or no radiation. The results are from the same batch of experiment shown in Figure 4E **(I)** Lipid peroxidation assessment in HULEC-5a cells with knockdown of VE-cadherin subjected to IR or no radiation. The results are from the same batch of experiment shown in Figure 4F **(J)** Western blot and RT-PCR analyses showing successful overexpression of VE-cadherin in HULEC-5a cells. **(K)** ROS measurement in HULEC-5a cells with overexpression of VE-cadherin with Yoda1 treatment. **(L)** Lipid peroxidation assessment in HULEC-5a cells with overexpression of VE-cadherin with Yoda1 treatment. **(M)** ROS measurement in HULEC-5a cells with overexpression of VE-cadherin with IR. The results are from the same batch of experiment shown in Figure 4K **(N)** Lipid peroxidation assessment in HULEC-5a cells with overexpression of VE-cadherin with IR. The results are from the same batch of experiment shown in Figure 4L **(O)** Transmission electron microscopy images of HULEC-5a cells with overexpression of VE-cadherin with Yoda1 treatment. **(P)** Transmission electron microscopy images of HULEC-5a cells with overexpression of VE-cadherin before IR. Data were plotted as means ± SEM. n = 3 independent repeats. **, *p* < 0.01 *vs* control; ^##^, *p* < 0.01 *vs* NC; ^%%^, *p* < 0.01 *vs* IR; ^$$^, *p* < 0.01 *vs* IR + NC (negative control; ^^^^, *p* < 0.01 *vs* Yoda1; ^++^, *p* < 0.01 *vs* Yoda1+NC).

We observed significantly lower VE-cadherin expression in pulmonary vascular endothelium in mice 24 h after irradiation compared with VE-cadherin expression in control (Figure 4A). In addition, degradation of VE-cadherin was increased in HULEC-5a cells after IR or after Yoda1 treatment compared with that in control group (Figures 4B, C). Specific siRNA was used to knockdown VE-cadherin expression in HULEC-5a cells (Figure 4D). Findings showed that silencing of VE-cadherin in HULEC-5a cells mimicked effects of PIEZO1 activation, which were confirmed by lipid peroxidation and accumulation of ROS (Figures 4E, F). Findings of the current study also showed ferroptosis-like alterations of mitochondria in HULEC-5a cells with si-VE-cadherin (Figure 4G). In addition, both lipid peroxidation and ROS accumulation were aggravated after si-VE-cadherin knockdown compared with those in the IR group alone, respectively (Figures 4H, I). Overexpression of VE-cadherin in HULEC-5a cells was confirmed by PCR and Western blot (Figure 4J). The experiments show that overexpression of VE-cadherin in HULEC-5a cells attenuated lipid peroxidation and ROS accumulation caused by Yoda1 and IR (Figure 4K–N). In terms of cell morphology, Yoda1 and IR-induced lung endothelial cells had increased mitochondrial density while overexpression of VE-cadherin in lung endothelial cells partly rescued this phenomenon (Figure 4O, P).

## Discussion

**FIGURE 5:**
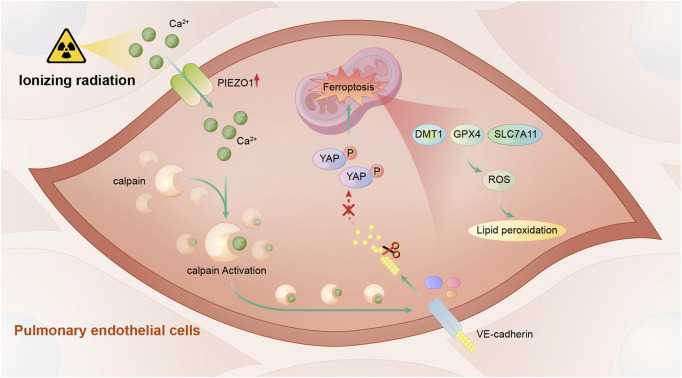
A schematic diagram summarizing the mechanism of PIEZO1 in regulating ionizing radiation-induced ferroptosis in lung endothelial cells. Ionizing radiation leads to increased PIEZO1 expression in lung endothelial cells. Increased expression of PIEZO1 increases intracellular Ca^2+^ concentration, which further increases calpain activity. Calpain increases degradation of VE-cadherin and promotes the development of ferroptosis possibly by YAP signaling.

In this study, we found that PIEZO1 expression in pulmonary endothelial cells was increased after ionizing radiation (IR). Increased PIEZO1 expression or activation of PIEZO1 could activate Ca^2+^/calpain signaling, which promoted the cleavage VE-cadherin, thereby promoting ferroptosis Inhibition of PIEZO1 activation, inhibition of calpain, or overexpression of VE-cadherin could mitigate IR-induced ferroptosis. The main findings of current study were summarized in Figure 5.

Ferroptosis plays crucial role in radiation-induced lung injury (RILI). Therefore, blocking ferroptosis could mitigate lung damage (Li X. et al., 2019). Different irradiation doses have distinct effects on cell fates (Lei et al., 2020). Irradiation of tumor cells at dose of 8-Gy induced ferroptosis in tumor cells (Lang et al., 2019). Guan et al. successfully constructed RILI model in mice by subjecting them to total radiation dose of 15-Gy of X-rays (Guan et al., 2018). The current study treated mice with the same dose of x-ray irradiation to demonstrate that ferroptosis is an important target for RILI. Findings showed significant alterations in ferroptosis-associated proteins in lung endothelial cells 24 h after 15-Gy radiation. In addition, transmission electron microscopy examination showed typical ferroptosis in endothelial cells subjected to radiation, indicating successful establishment of cell model of radiation-induced ferroptosis.

Solute carrier family 7, member 11 (SLC7A11), also known as xCT and glutathione peroxidase 4 (GPX4) are two key regulators of ferroptosis. GPX4 utilizes glutathione (GSH) to detoxify lipid peroxidation (Zhang et al., 2021). SLC7A11 imports cystine for GSH synthesis and protect cells against ferroptosis (Lei et al., 2021). In addition, regulation of ferritin and steady state of iron metabolism are important regulatory elements for ferroptosis (Torti and Torti, 2020). Plasmalemmal divalent metal ion transporter 1 (DMT1) is responsible for iron uptake and is an important transmembrane iron transporter that regulates iron metabolism (Torti and Torti, 2020). Previous studies have shown that up-regulation of DMT1 induces ferroptosis and knockout of DMT1 significantly reduces ferroptosis in cardiomyocytes (Song et al., 2021). Overexpression of DMT1 in hippocampal neurons causes iron overload and further leads to ferroptosis (Li et al., 2021). Findings of the current study showed changes in pulmonary microvascular endothelial cells which were consistent with changes in lung tissues. Ferroptosis induction by irradiation or PIEZO1 activation is accompanied by decreased expression of GPX4 and SLC7A11, and increased expression of DMT1. However, initiating factor of ferroptosis among the three proteins remains unknown.

Previous studies aver that PIEZO1 is a highly expressed mechanosensitive ion channel in lung endothelium (Iring et al., 2019). Some previous studies support role of PIEZO1 signaling in iron overload. Two gain-of-function mutants were found to be associated with increased intracellular Ca^2+^, which further inhibited the BMP-SMADs pathway, resulting in iron overload (Andolfo et al., 2020). E756del, a mild gain-of-function PIEZO1 mutant found in one-third of individuals of African descent, is a strong indicator of increased plasma iron (Ma et al., 2021). The current study explored the role of PIEZO1 in radiation-induced ferroptosis, and established that PIEZO1 activation induced ferroptosis in pulmonary microvascular endothelial cells (Figure 2), whereas PIEZO1 inhibition partly attenuated radiation-induced ferroptosis. These findings indicate that PIEZO1 was a potent regulator of ferroptosis.

Previous studies demonstrated that PIEZO1 mediates neuronal oxygen-glucose deprivation injury through Ca^2+^/calpain signaling pathway (Wang et al., 2019). As intracellular Ca^2+^-dependent cysteine protease, calpain is also involved in PIEZO1-induced pulmonary endothelial barrier disruption in acute respiratory distress syndrome (Zhong et al., 2020; Jiang L. et al., 2021). Calpain activation also mediates effects of PIEZO1 on apoptosis of prostate cells (Hope et al., 2019). Oxidative stress is closely related with ferroptosis. Specific calpain-1 inhibitor rescues cell damage caused by oxidative stress in brain endothelial cells (Knopp et al., 2021). Previous studies established that inhibition of calpain activity efficiently prevents high glucose-induced ROS production in human umbilical vein endothelial cells (Chen et al., 2014). The current study further determined whether PIEZO1 mediates ferroptosis in pulmonary vascular endothelial cells *via* the Ca^2+^/calpain signaling pathway. Firstly, the current study demonstrated that Yoda1 activation of PIEZO1 and radiation caused increase in calcium concentration and up-regulation of calpain activity. The current study then tested calpain inhibitors (PD151746) for their anti-ferroptosis effects. PD151746 reversed lipid peroxidation induced by IR and Yoda1 and reduced ROS production. In addition, PD151746 ameliorated decreased expression of GPX4 and SLC7A11 after IR. Findings of the current study demonstrated important role of PIEZO1/Ca^2+^/calpain signaling in modulating ferroptosis. A shortcoming of the current study is that it did not interfere with intracellular Ca^2+^ concentration in lung microvascular endothelial cells. Moreover, the current study did not clarify the respective roles of calpain 1 or calpain 2 in ferroptosis in lung endothelial cells.

VE-cadherin acts as downstream of mechanical ion channel, PIEZO1, and regulates response of endothelial cells to fluid shear stress (Siragusa et al., 2021). Findings of the current study indicated that degradation of VE-cadherin is enhanced when Yoda1 activates PIEZO1 or when cells are exposed to radiation. Calpain inhibitor (PD151746) blocked these effects. VE-cadherin participates in ferroptosis-like mechanism by increasing endothelial cell junction gaps (Lopes-Coelho et al., 2021). Furthermore, E-cadherin, analog of VE-cadherin, has been shown to regulate ferroptosis through activating intracellular NF2-YAP signaling pathway (Wu et al., 2019b). The current study further established that knockdown of VE-cadherin in endothelial cells caused increased ROS and lipid peroxidation while overexpression of VE-cadherin partly rescued these ferroptosis-related changes caused by IR, supporting that PIEZO1 promotes ferroptosis via calpain-dependent cleavage of VE-cadherin in endothelial cells. Jiao et al. demonstrated involvement of E-cadherin in ferroptosis in epithelial cells through ACSL4-related mechanisms (Wu et al., 2019a). However, these findings are inconsistent with results of the current study, where expression of ACSL4 did not change significantly in HULEC-5a cells of silenced VE-cadherin. There is a need to establish whether there exist different mechanisms of ferroptosis between VE-cadherin and E-cadherin. Moreover, it is unclear as to whether IR can also induce pulmonary endothelial cell ferroptosis in endothelium-specific PIEZO1 knockout mice.

## Conclusion

In conclusion, the current study demonstrated that lung endothelial cell ferroptosis modulated by PIEZO1/Ca^2+^/calpain signaling is a potential therapeutic mechanism for the treatment of radiation-induced lung injury.

## Data Availability

The original contributions presented in the study are included in the article/Supplementary Material, further inquiries can be directed to the corresponding authors.
