# Supplementary material for: PIEZO1 Ion Channel Mediates Ionizing Radiation-Induced Pulmonary Endothelial Cell Ferroptosis via Ca2+/Calpain/VE-Cadherin Signaling
Source: Front Mol Biosci. 2021 Sep 9;8:725274. doi: 10.3389/fmolb.2021.725274 (PMC8458942; doi:10.3389/fmolb.2021.725274)
Supplement: Supplementary file 1 [file DataSheet2.PDF]

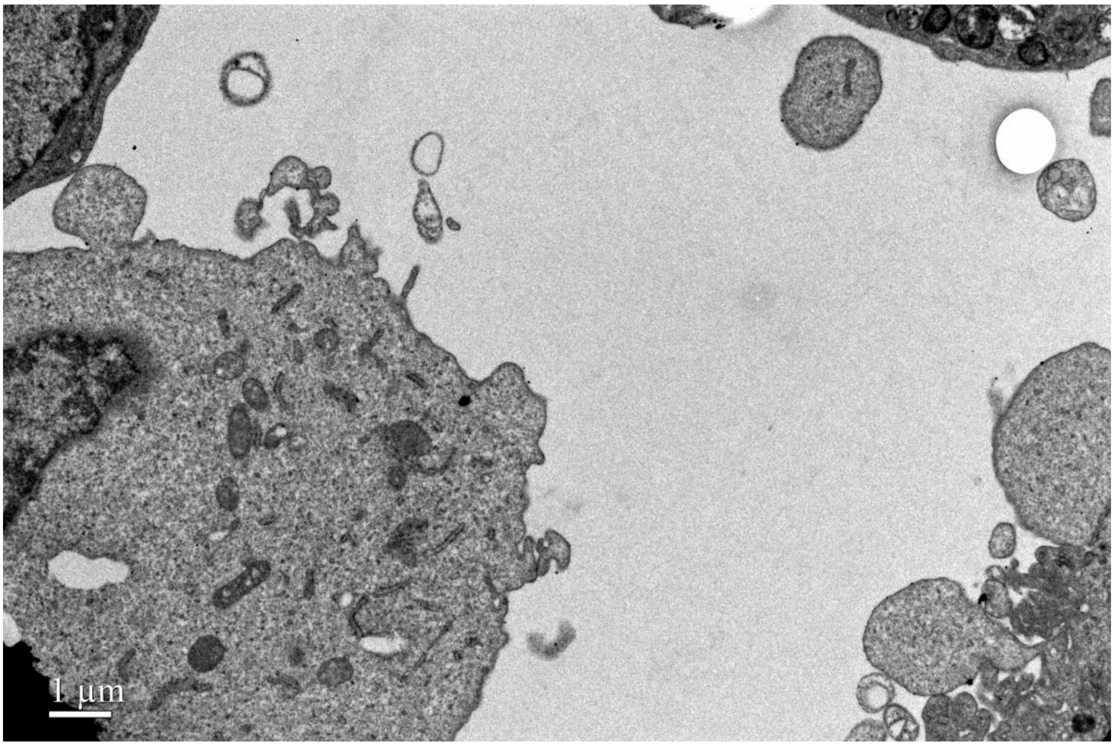
1 µm

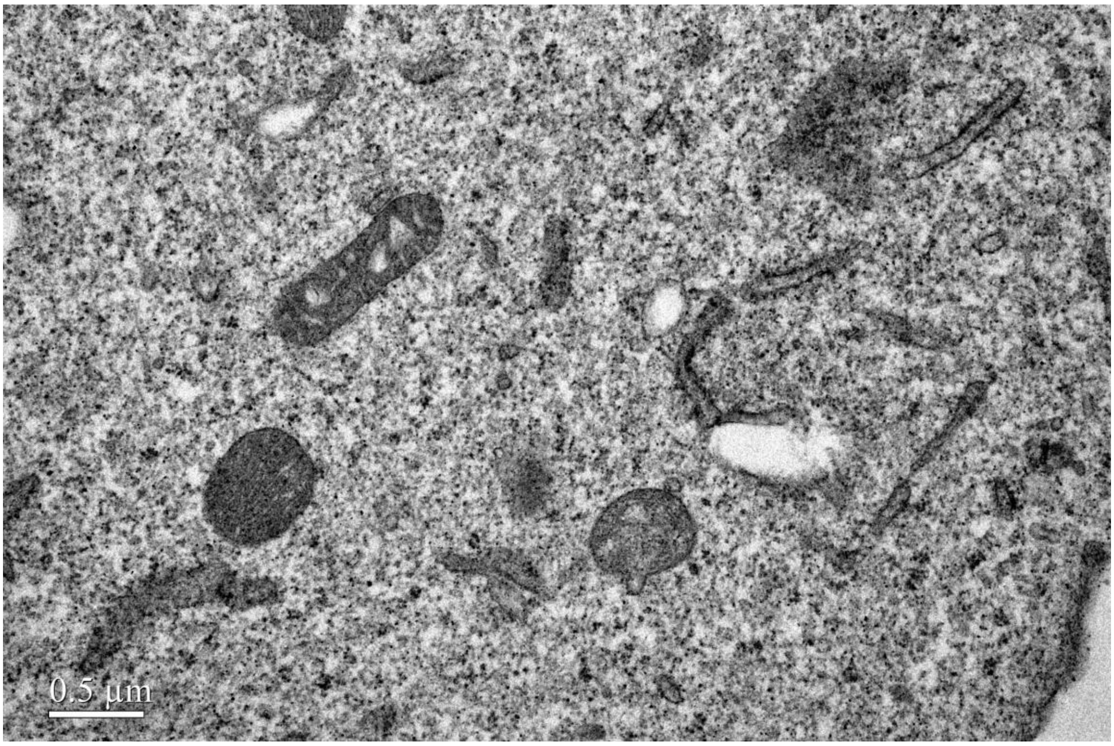
0.5 µm

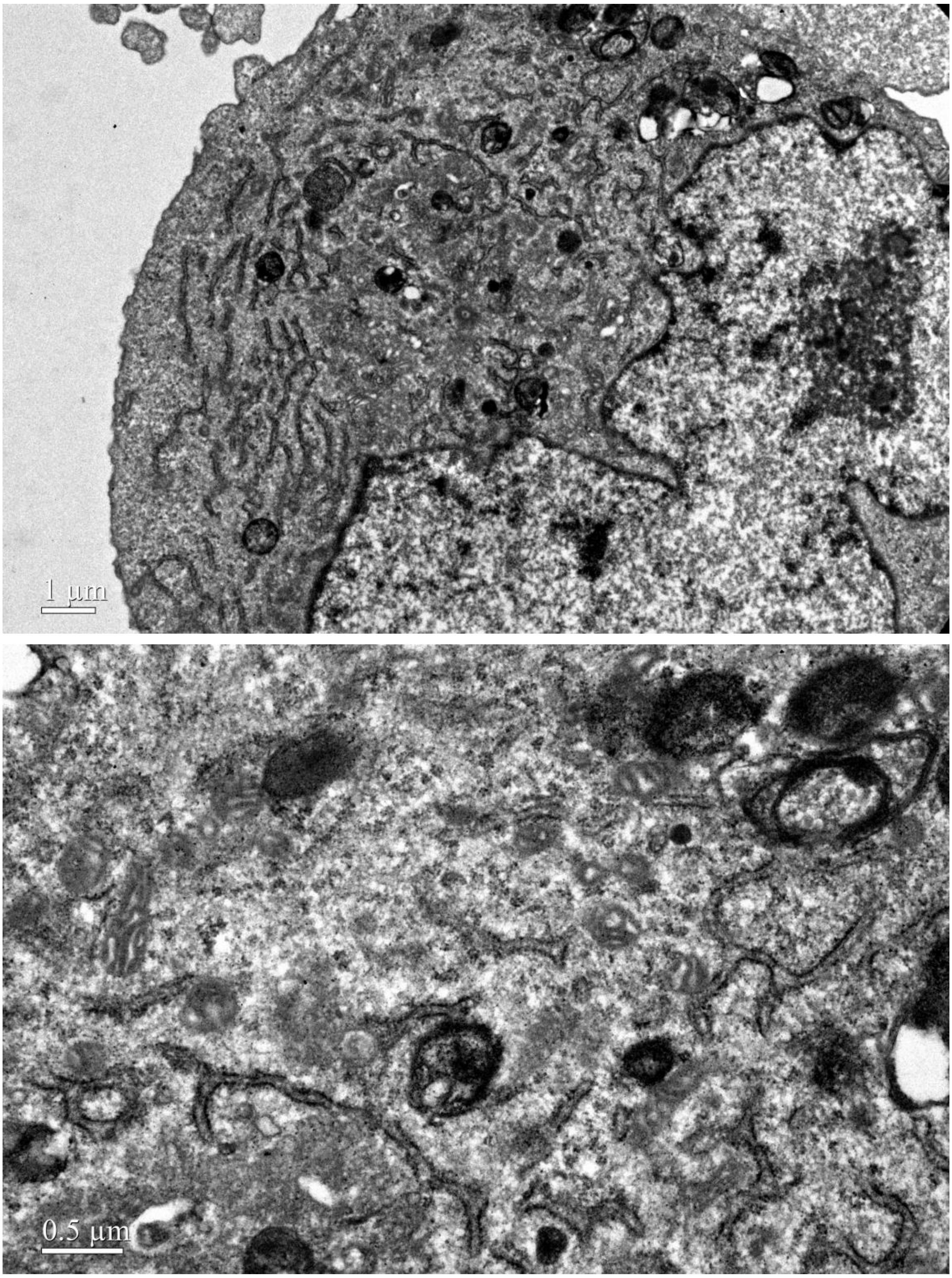

Figure S1 A the original microscopy images displayed in **Figure1 D**

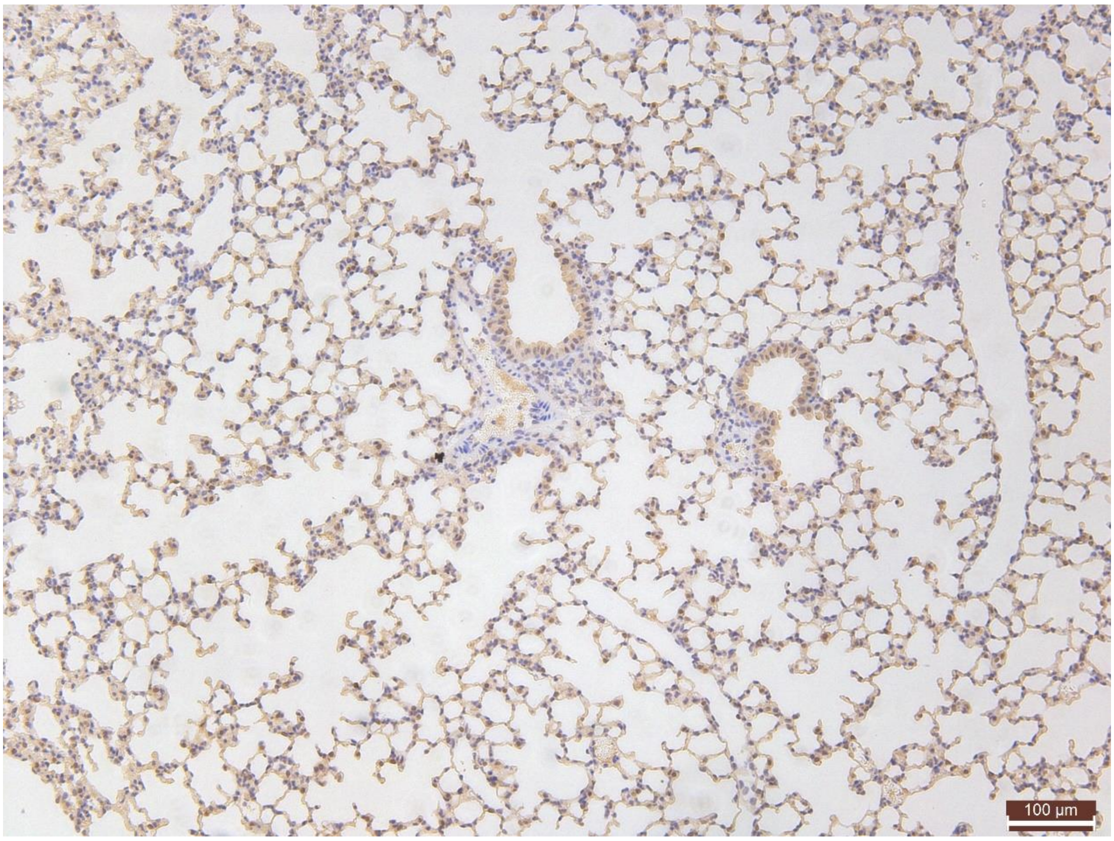
100 µm

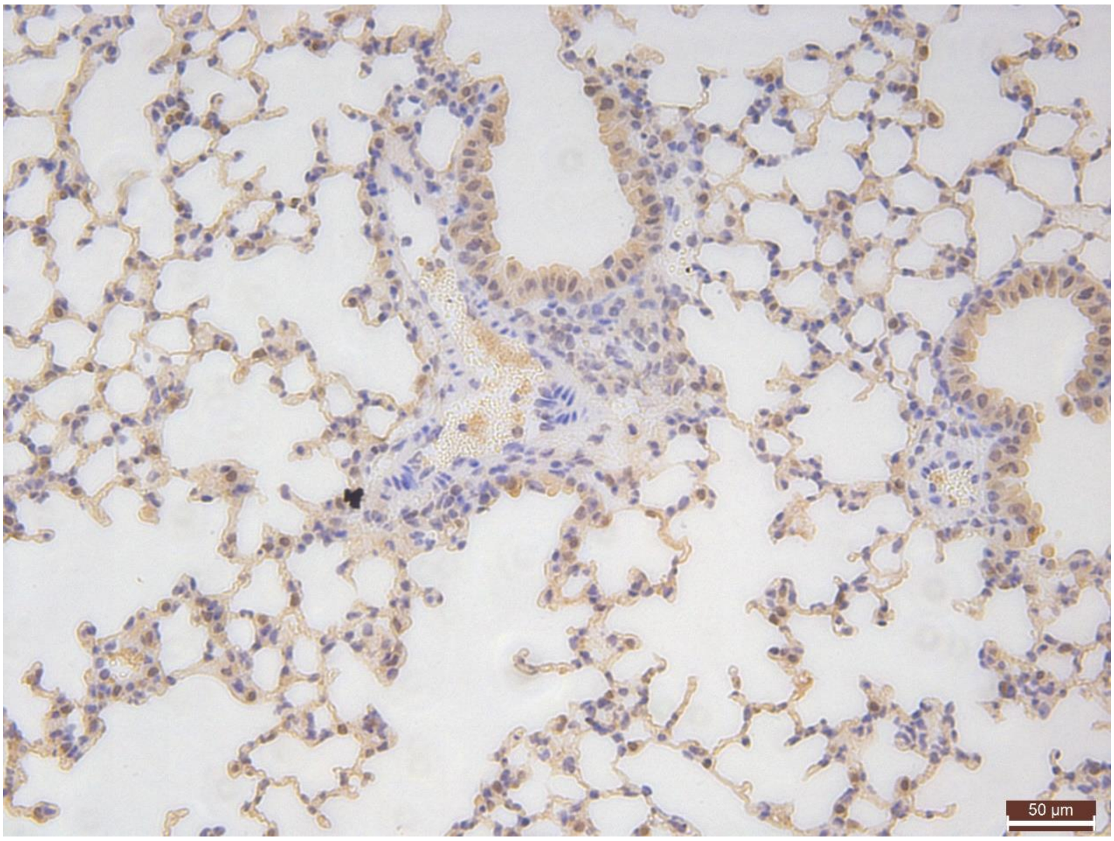
50 µm

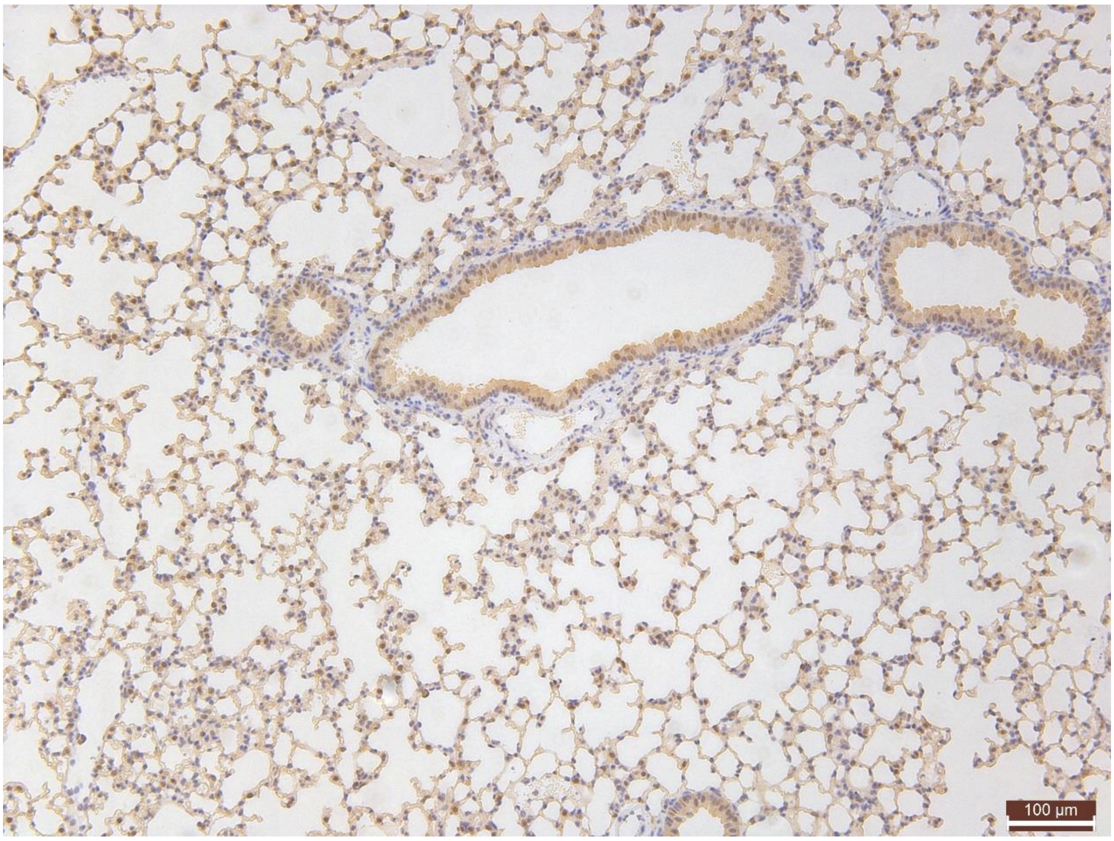
100 µm

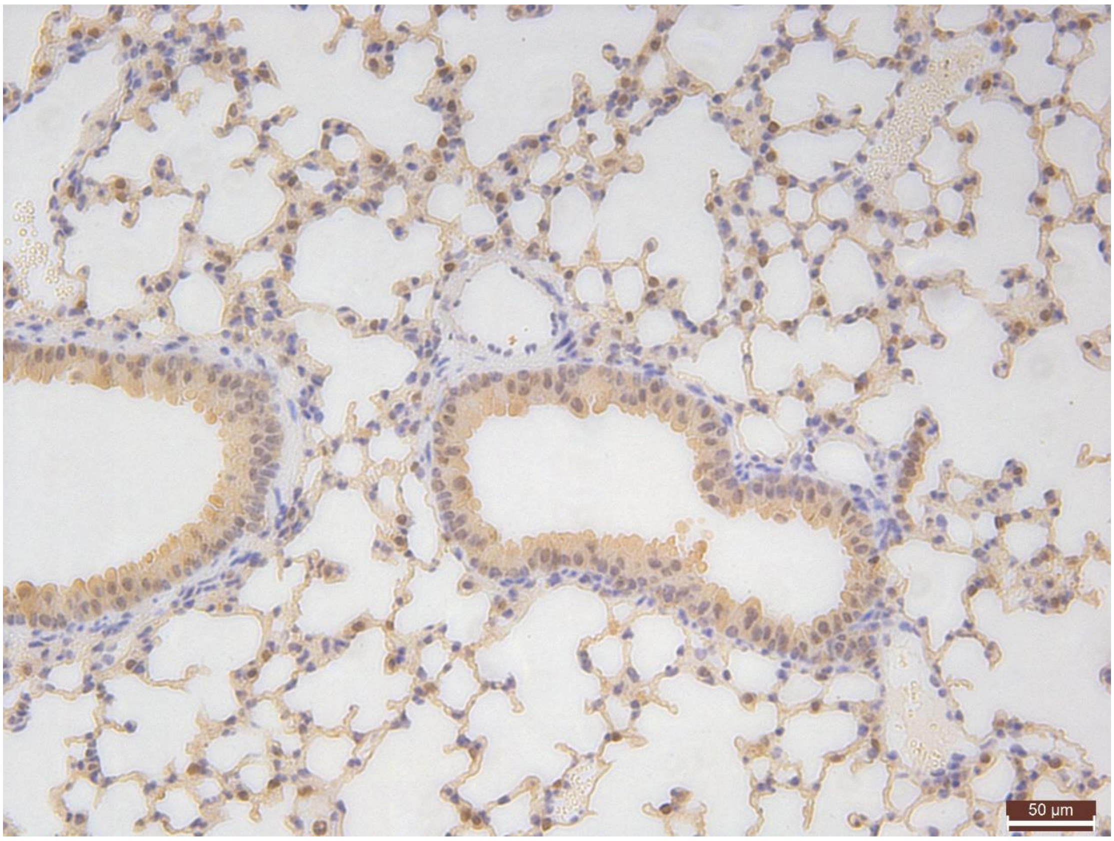
50 µm

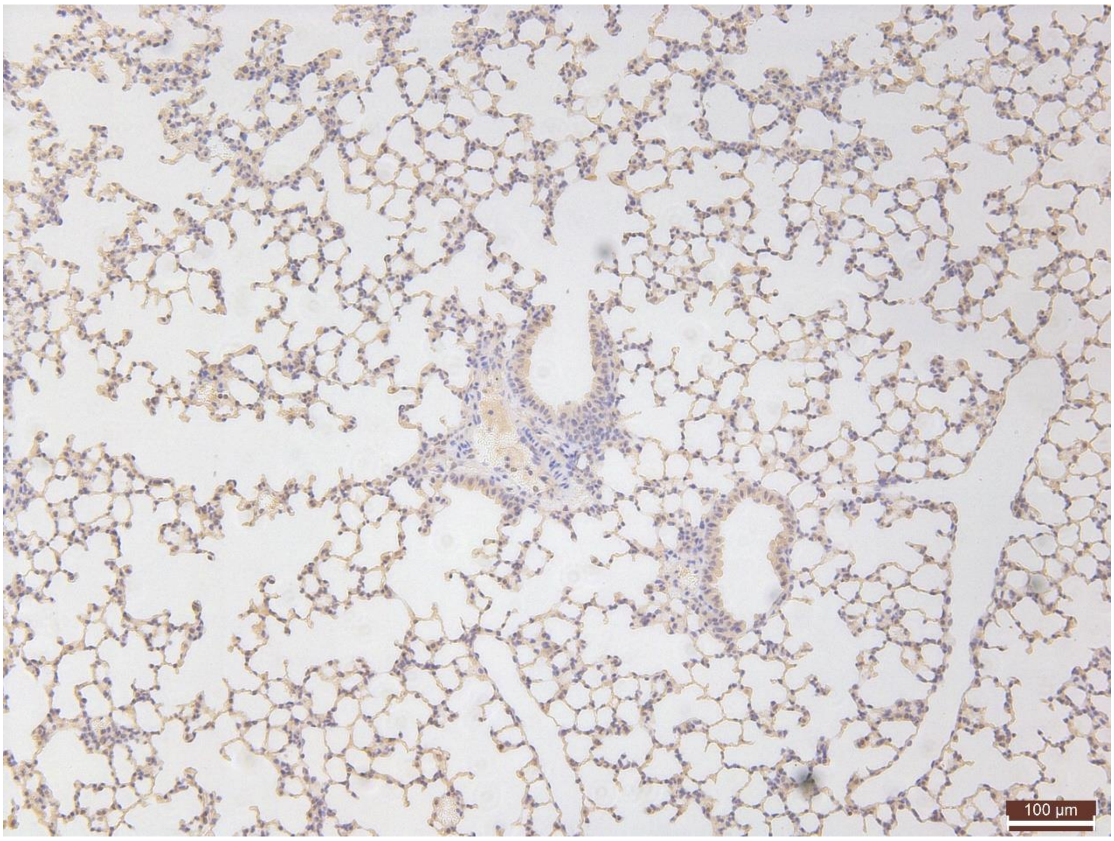
100 μm

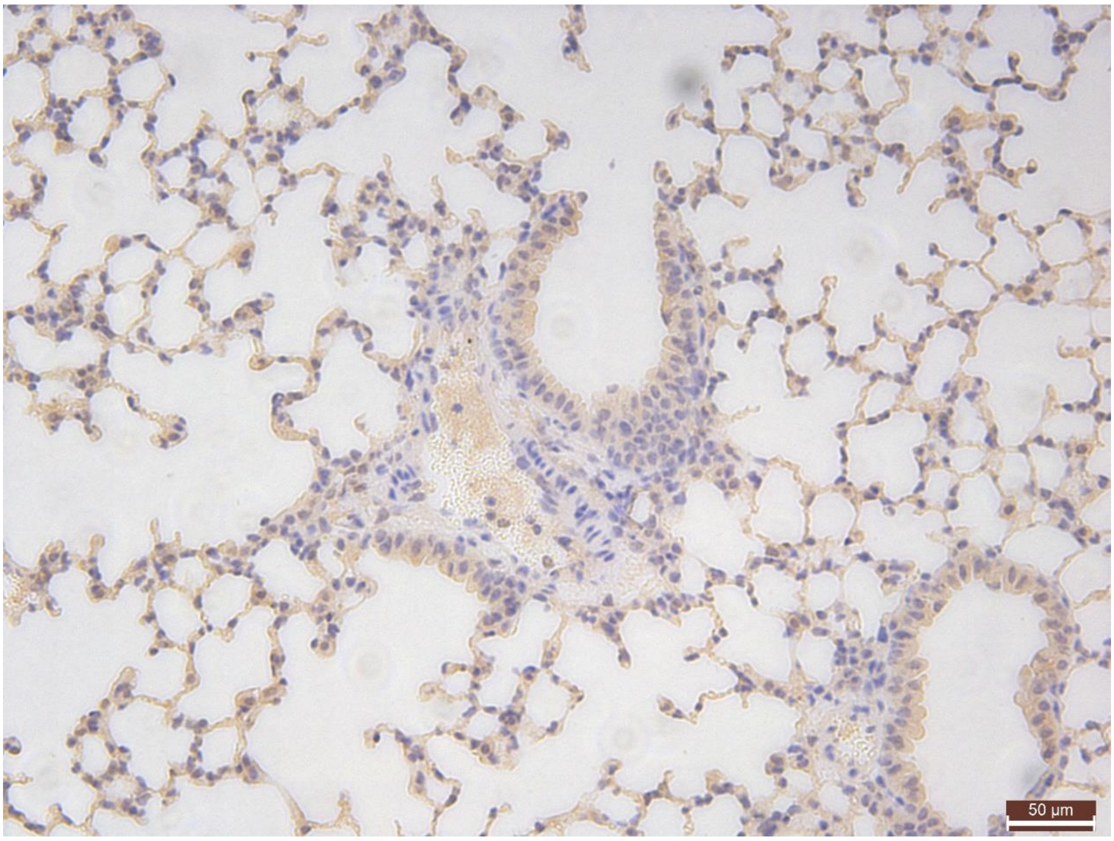
50 μm

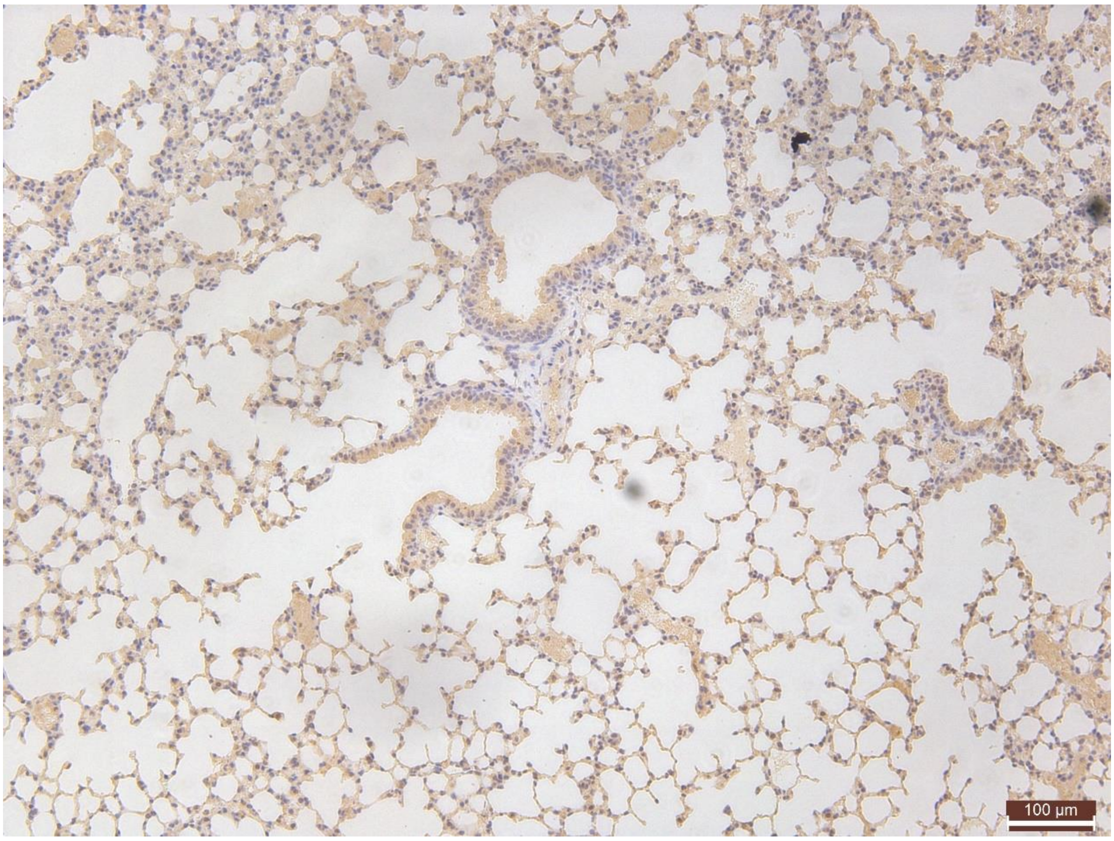

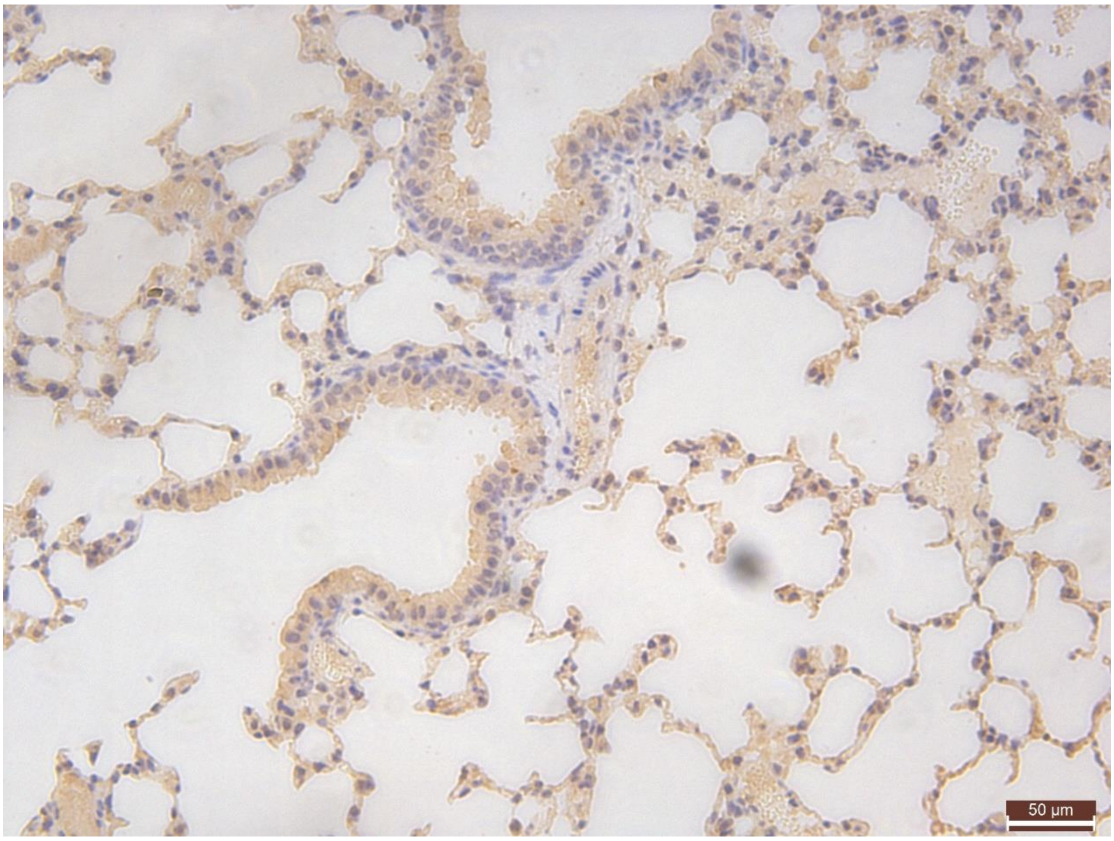

Figure S1 B the original microscopy images displayed in **Figure1 E**

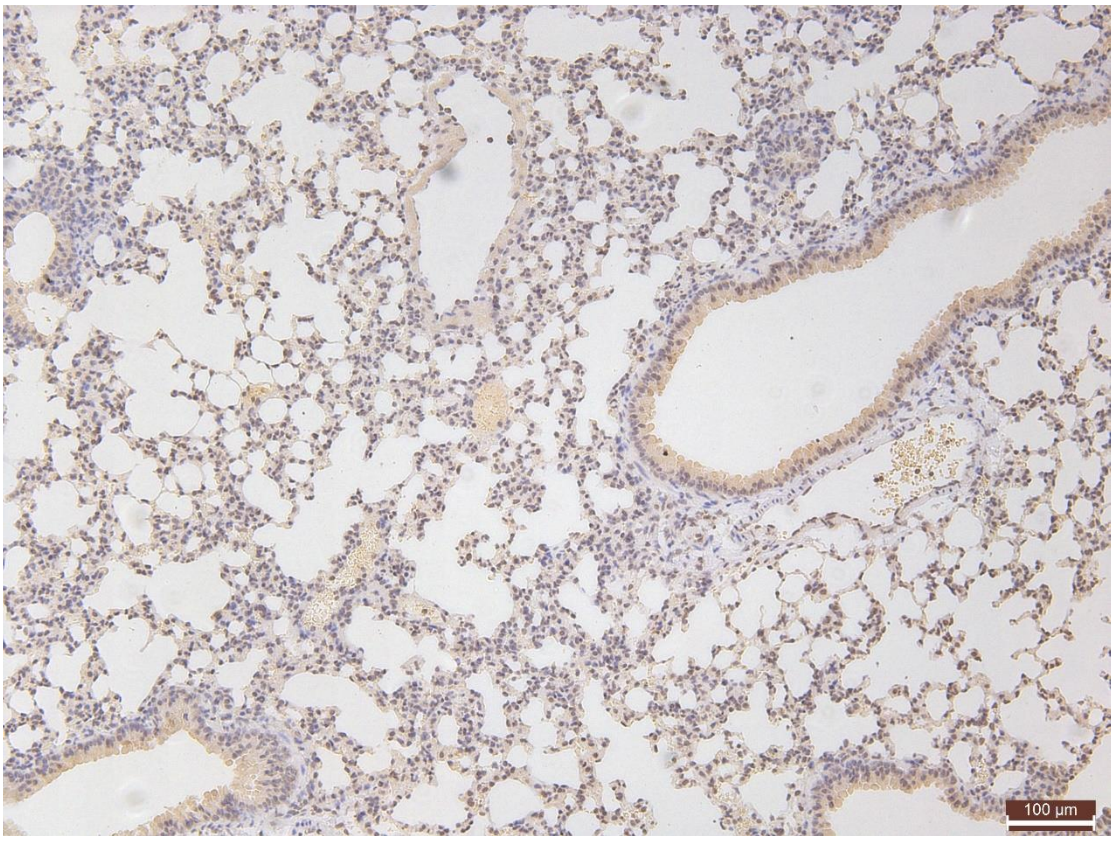
100 µm

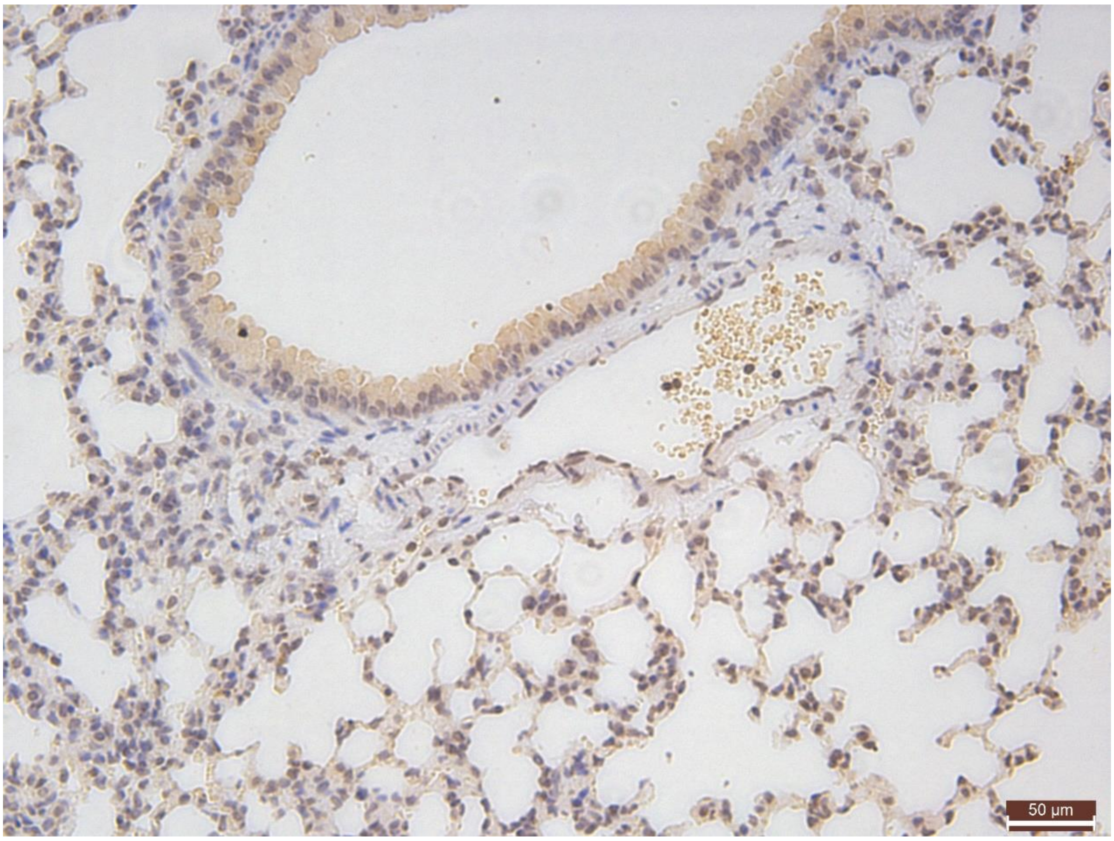
50 µm

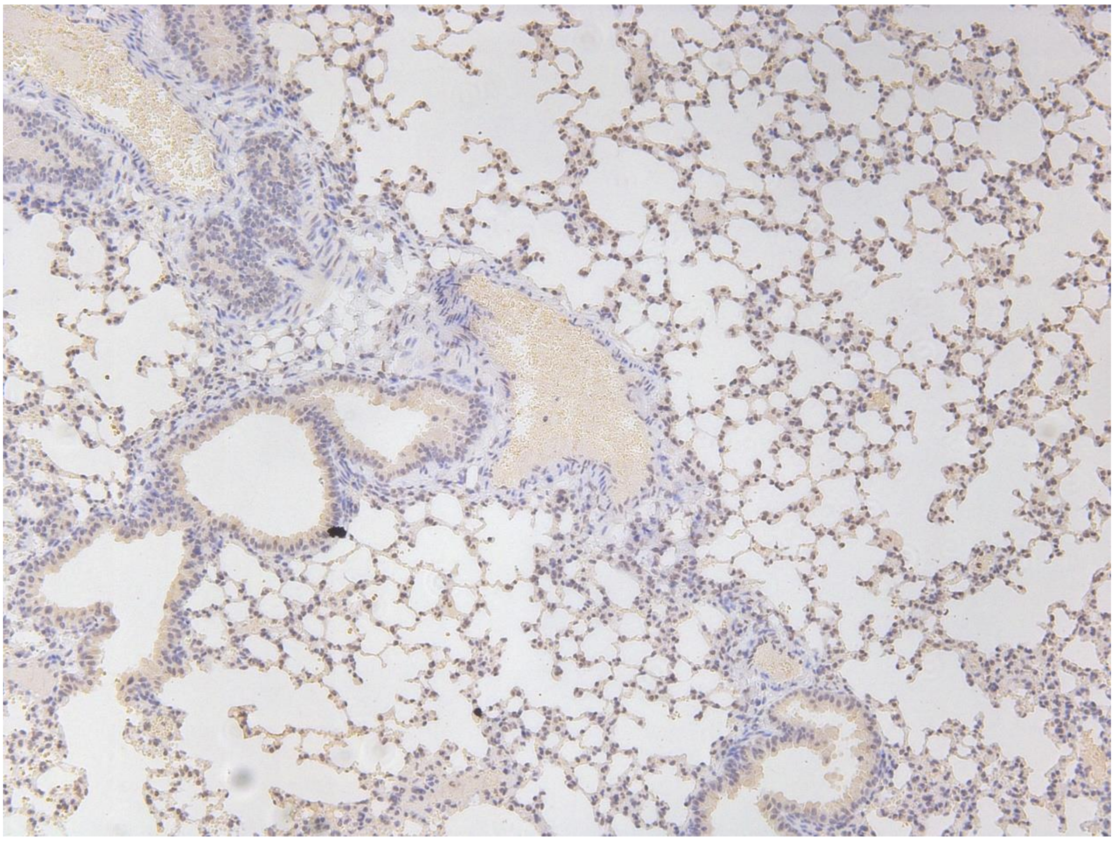

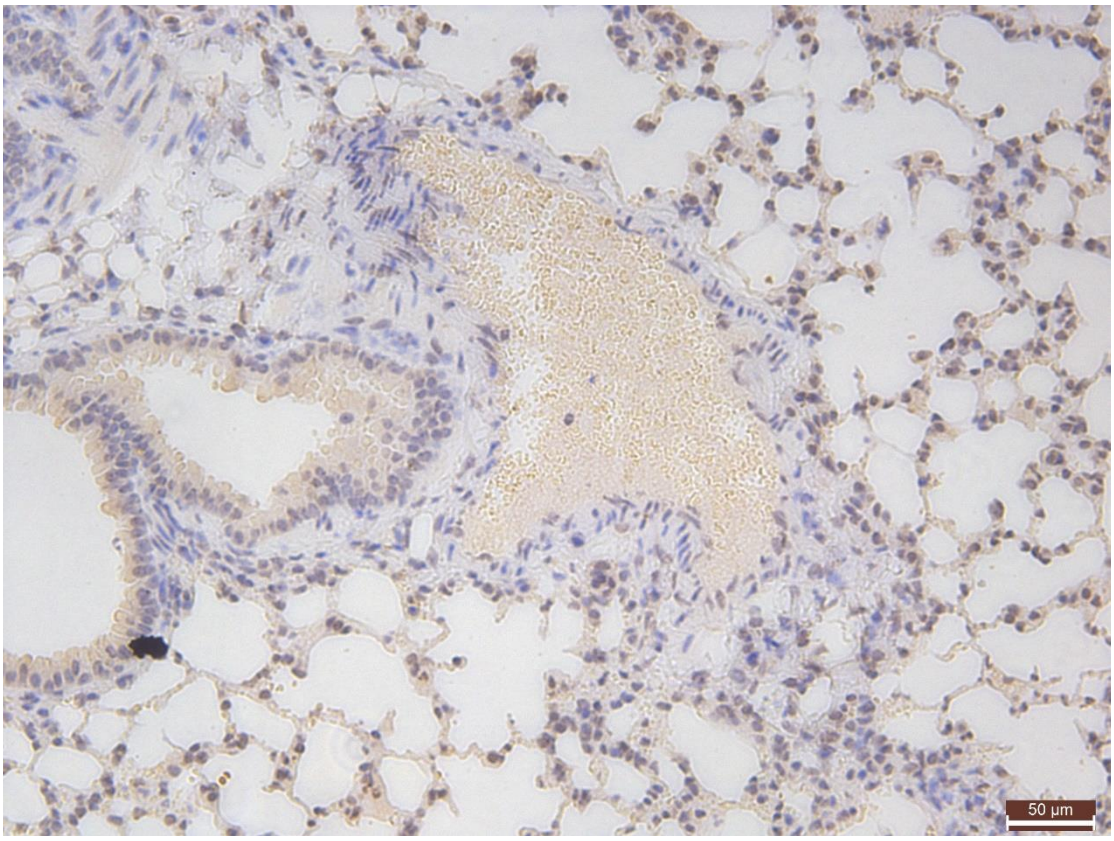

Figure S1 C the original microscopy images displayed in **Figure1 G**

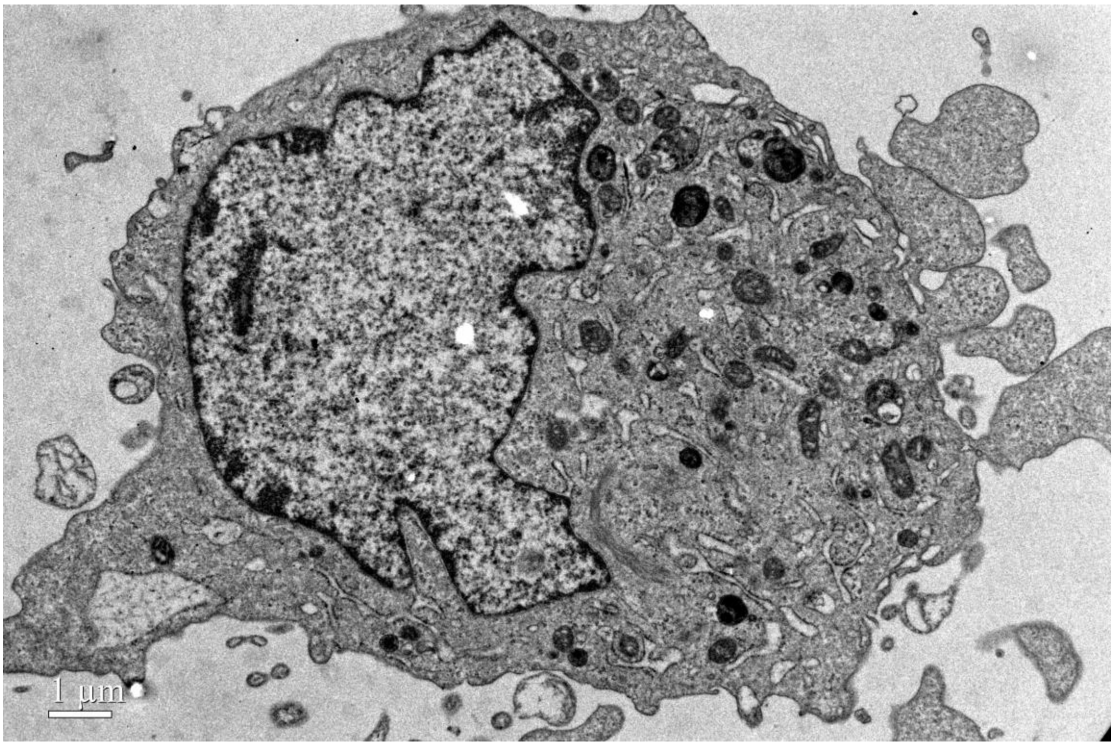
1 µm

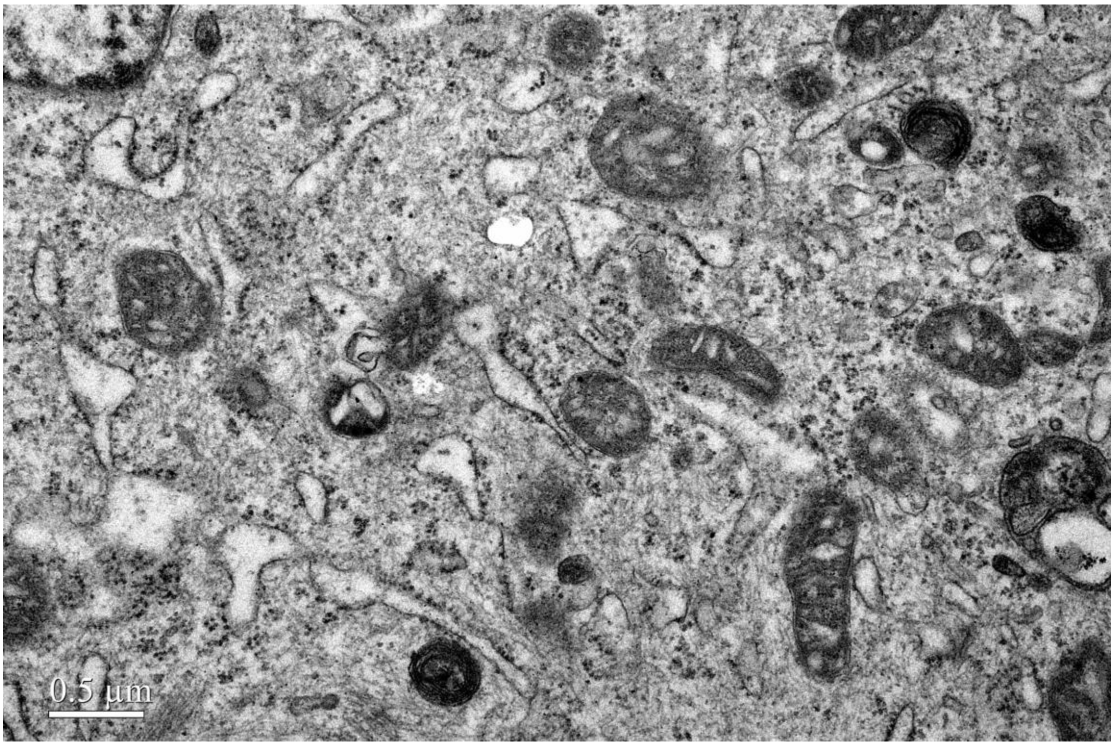
0.5 µm

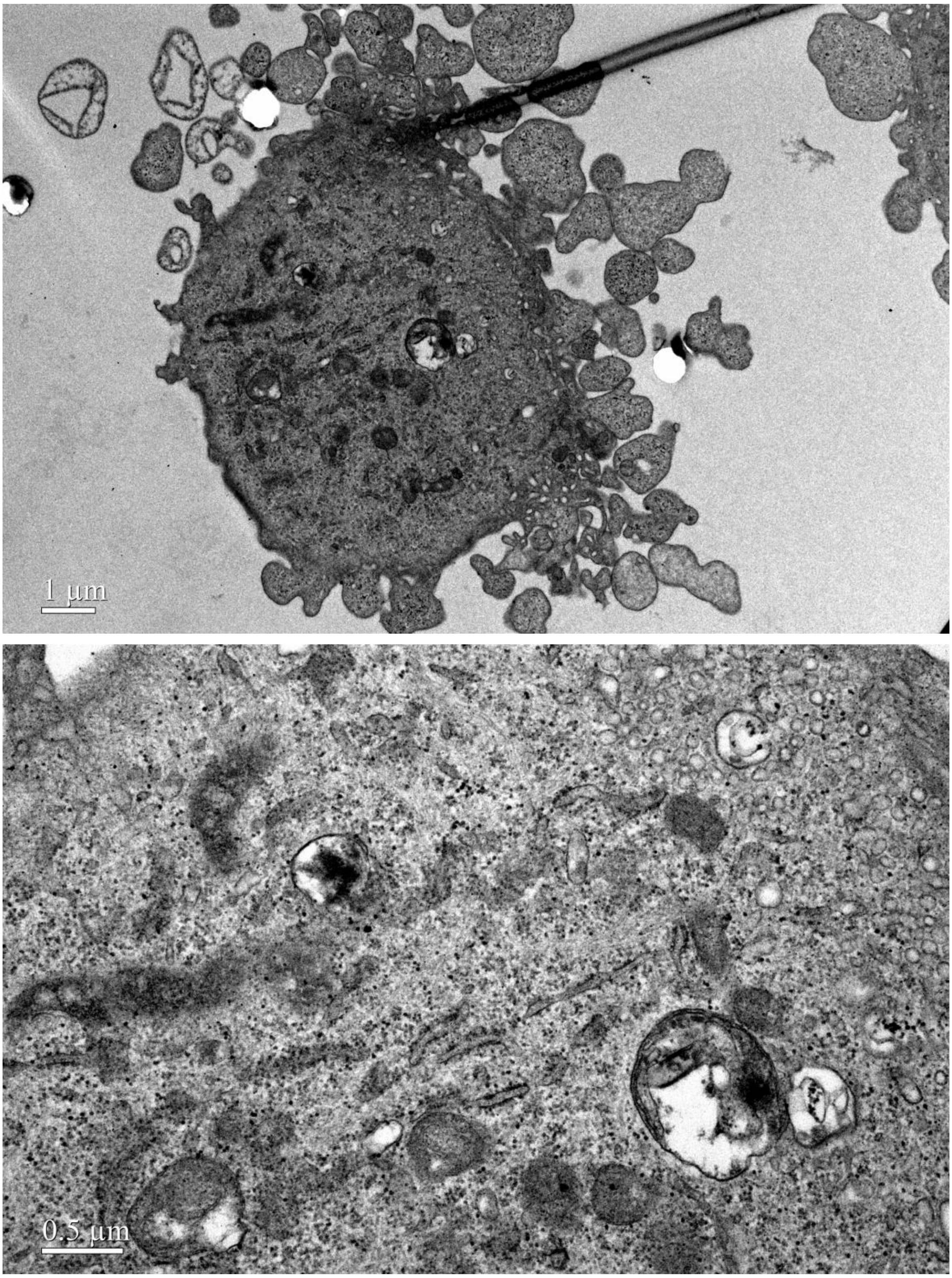

Figure S2 A the original microscopy images displayed in **Figure2 D**

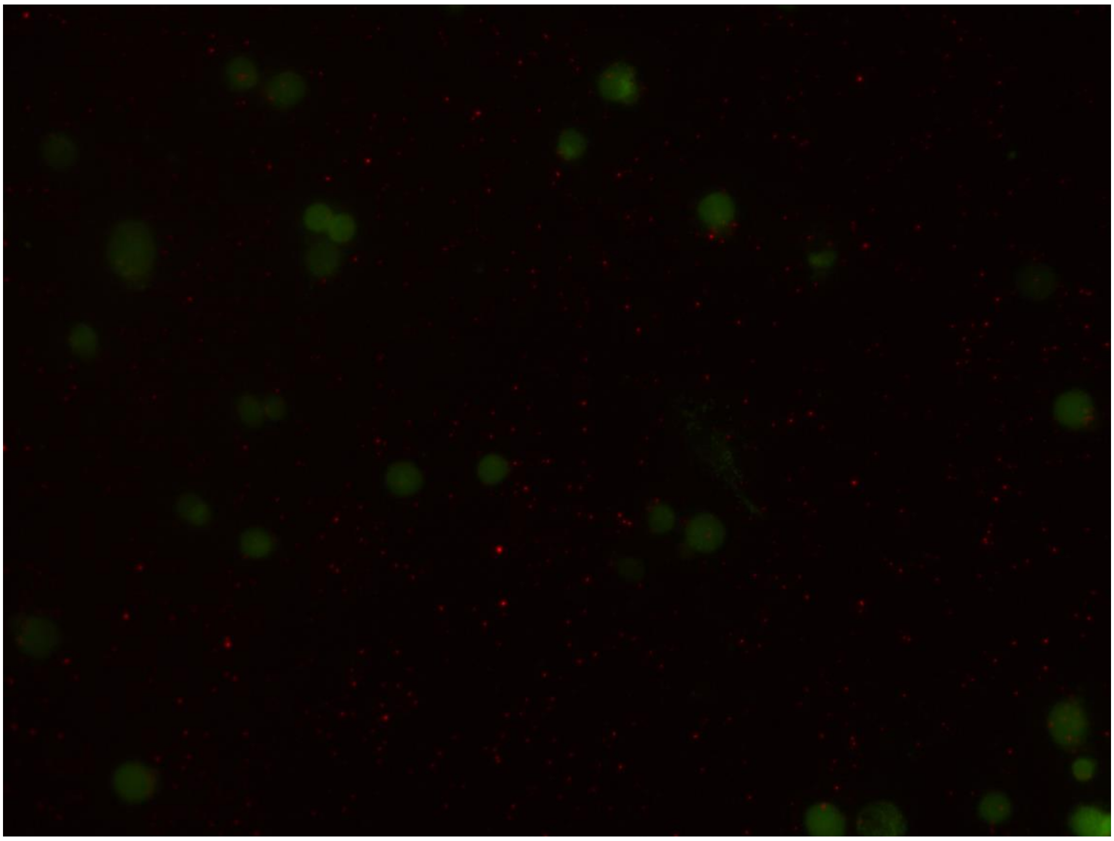

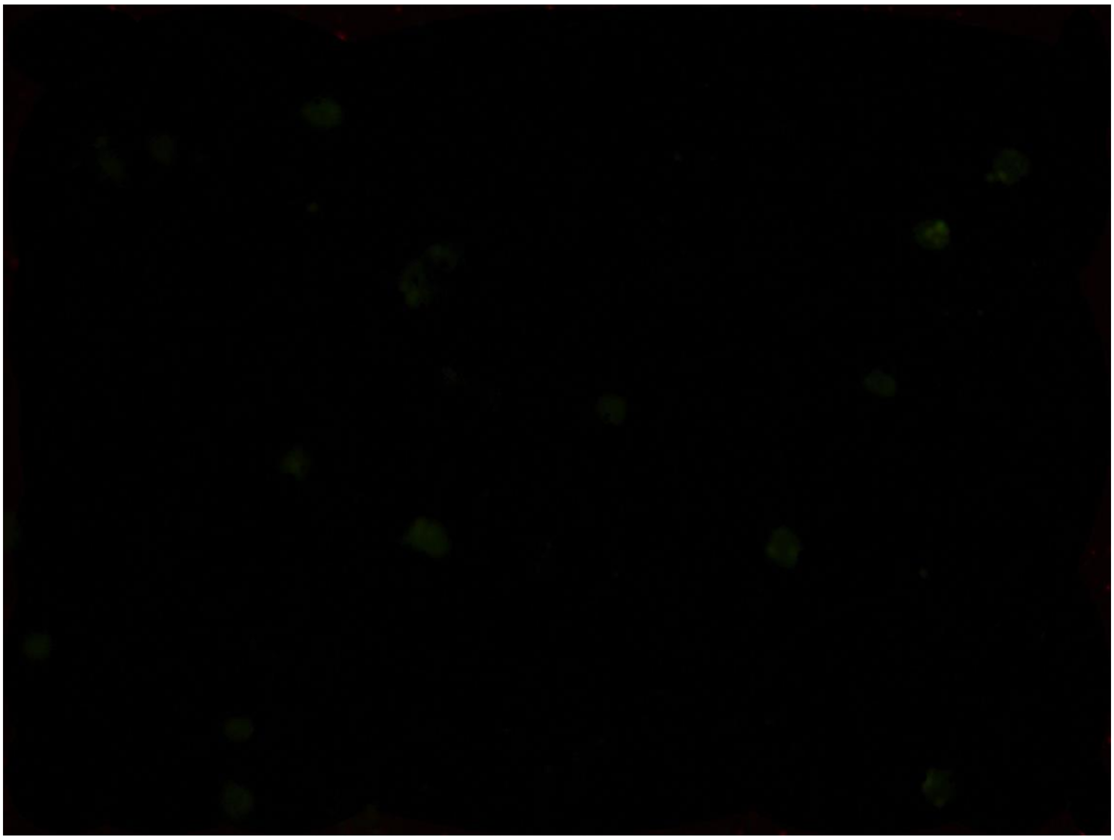

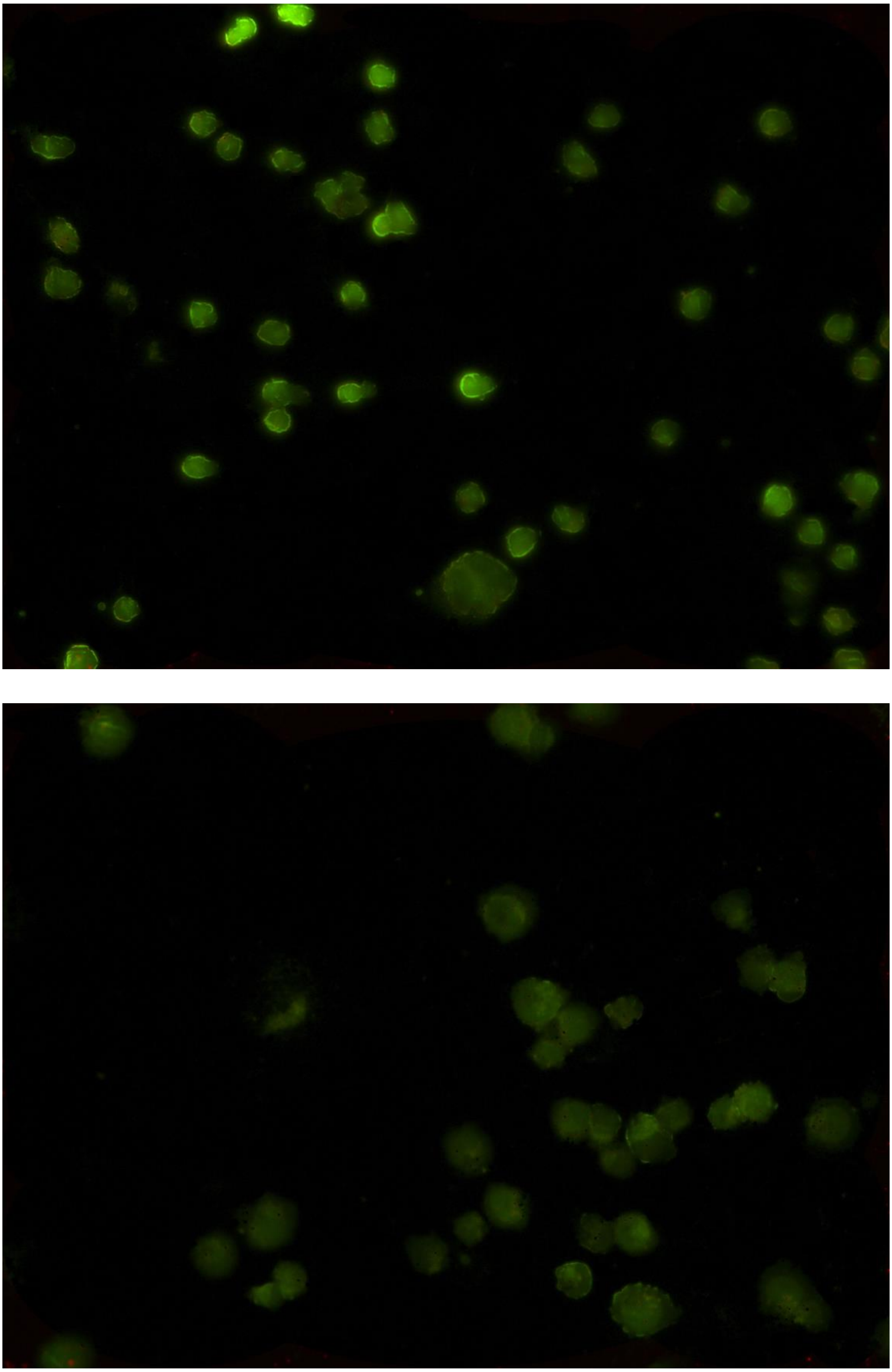

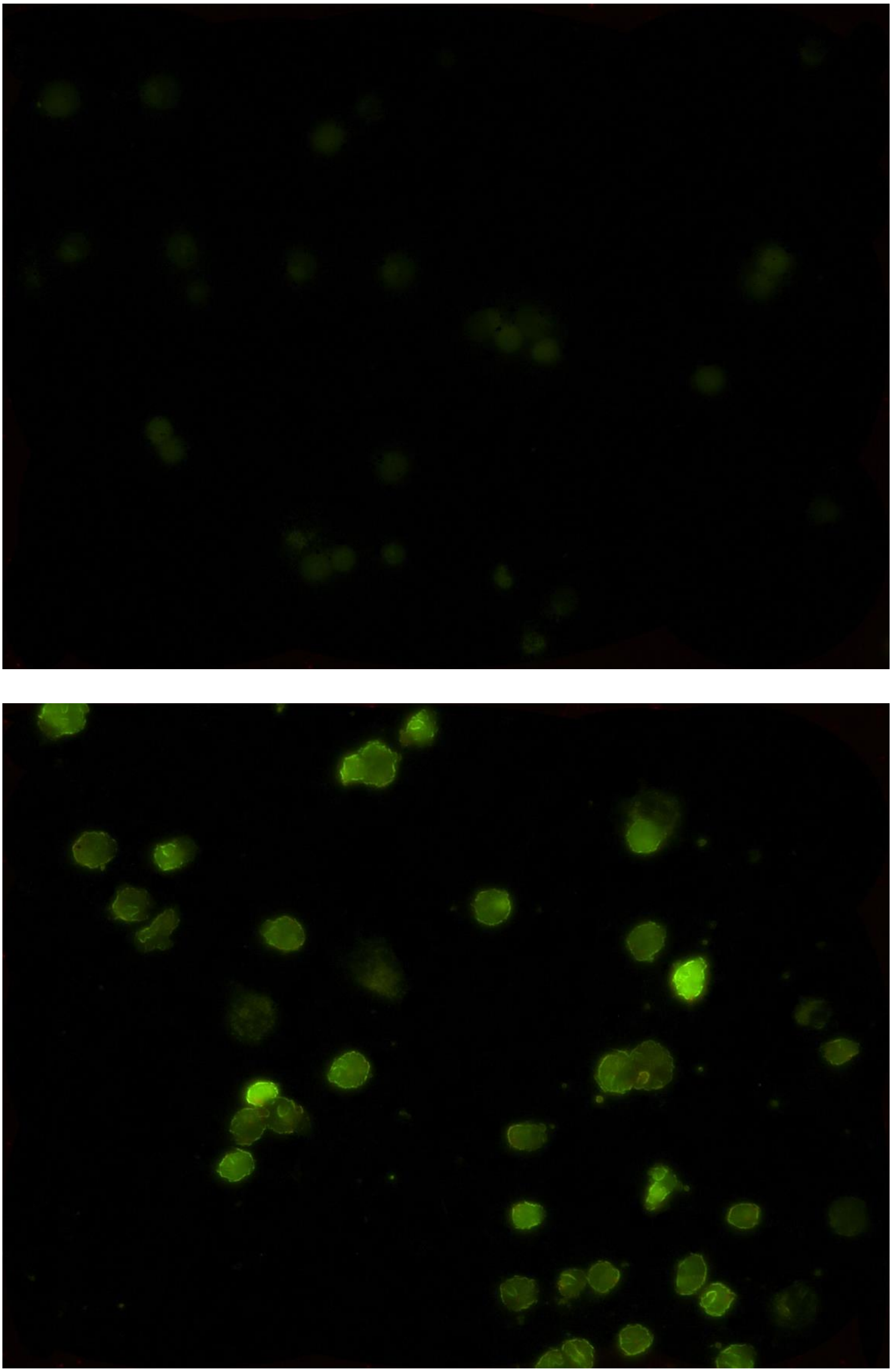

Figure S3 A the original microscopy images displayed in **Figure3 A**

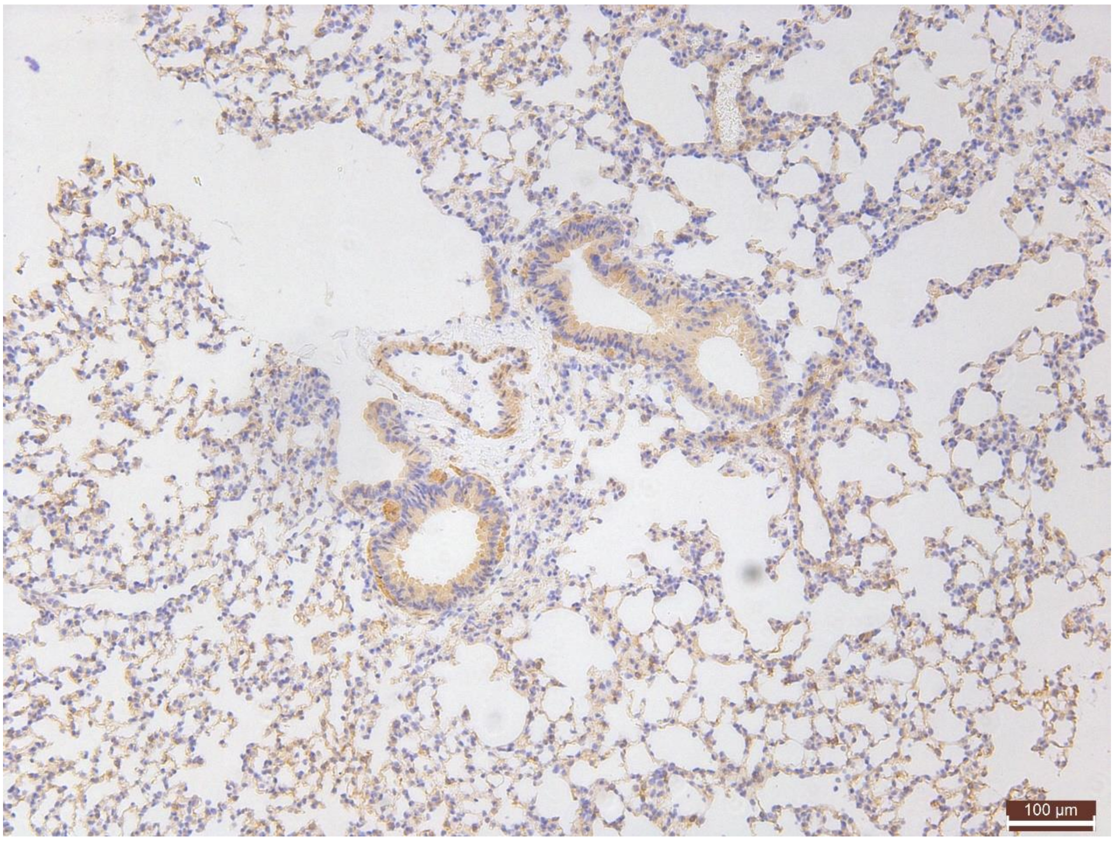
100 µm

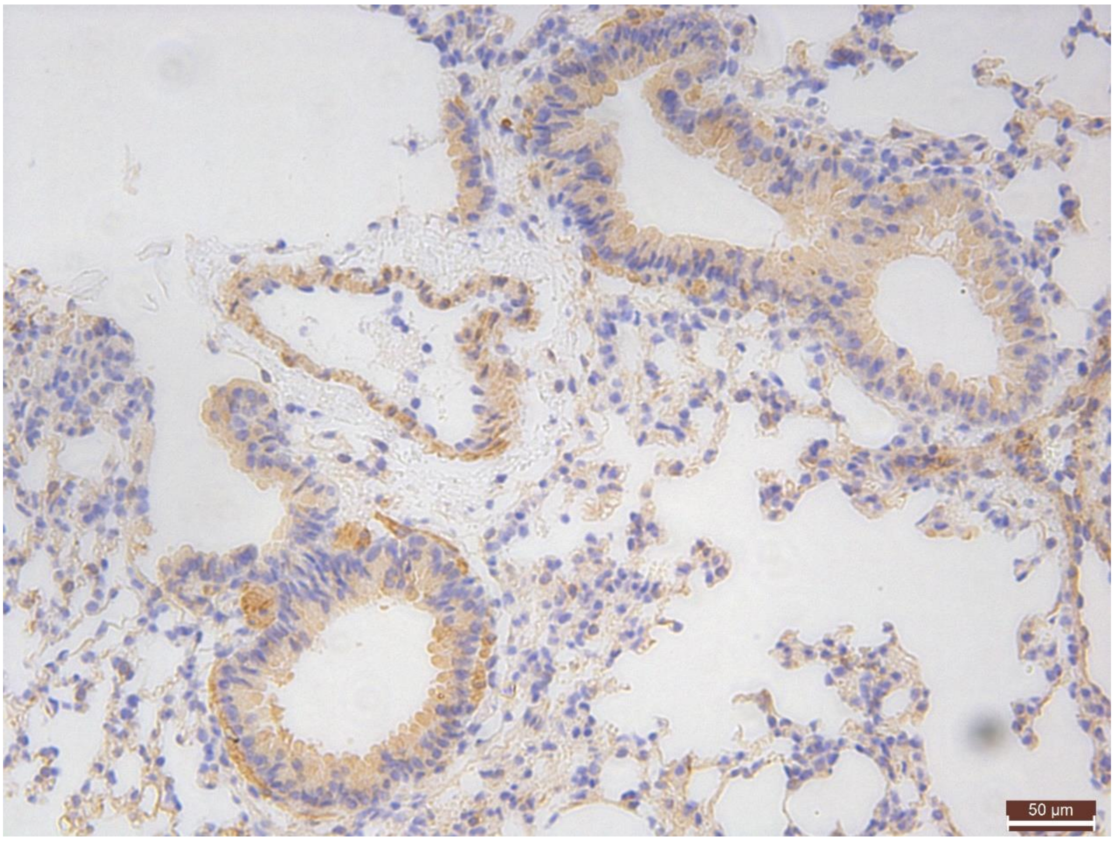
50 µm

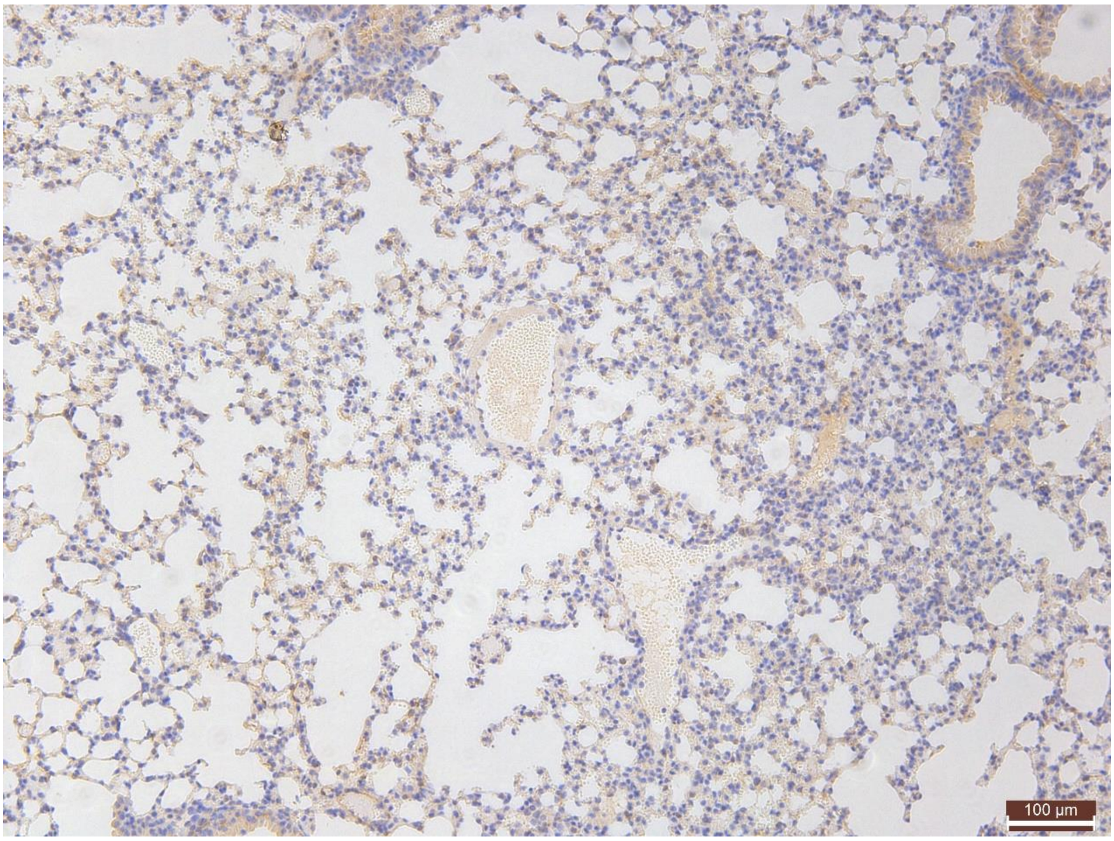

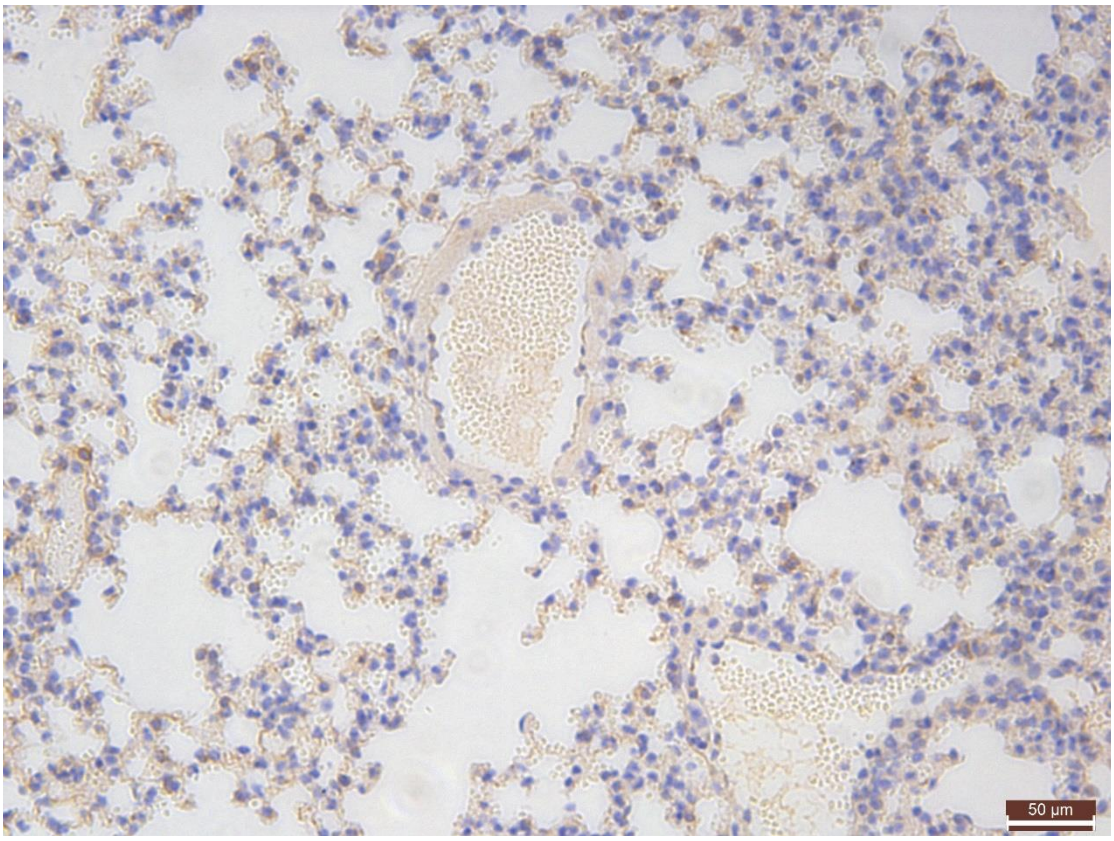

Figure S4 A the original microscopy images displayed in **Figure4 A**

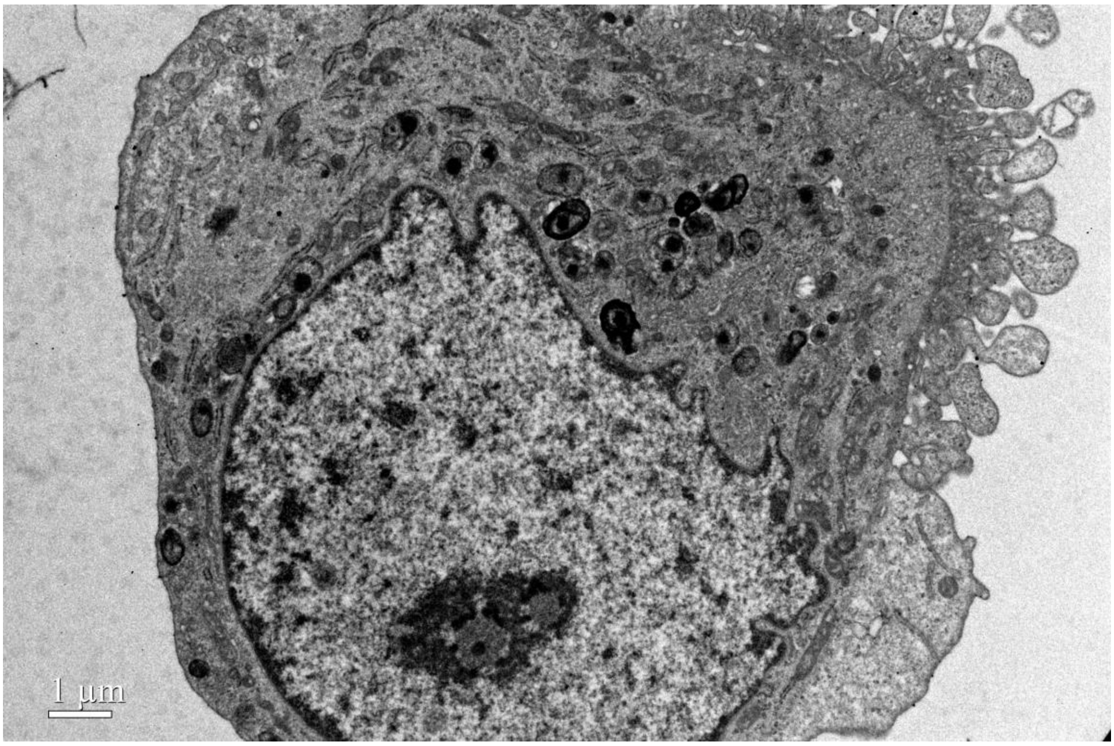
1 µm

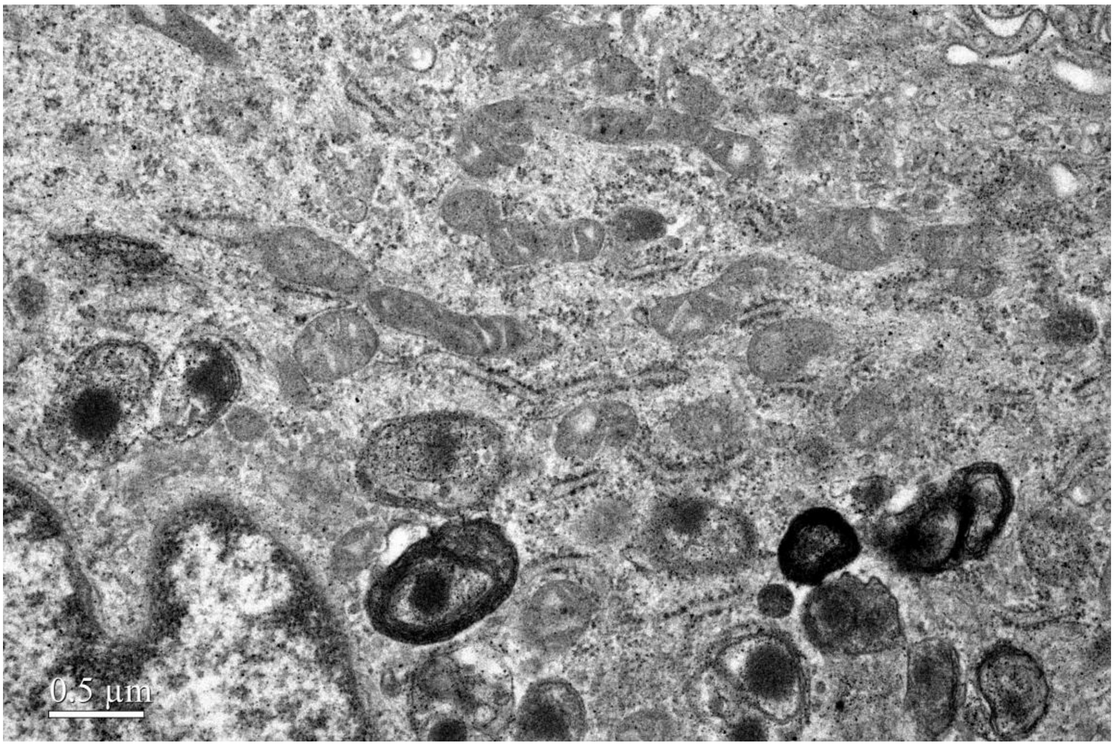
0.5 µm

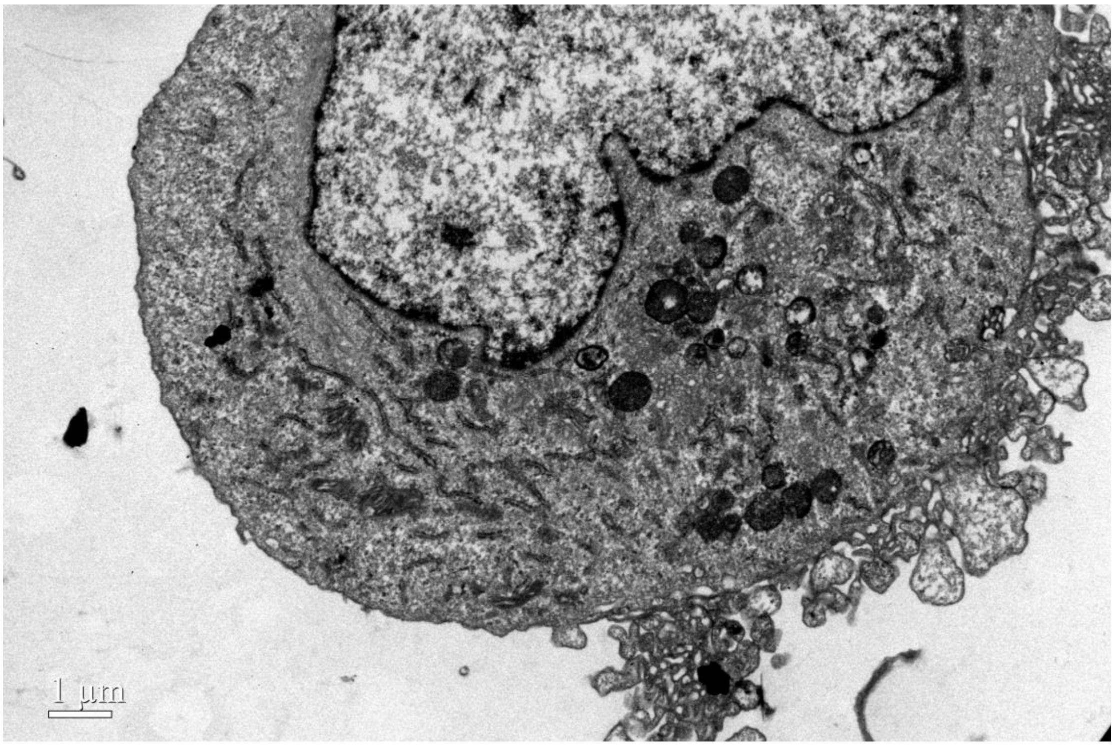

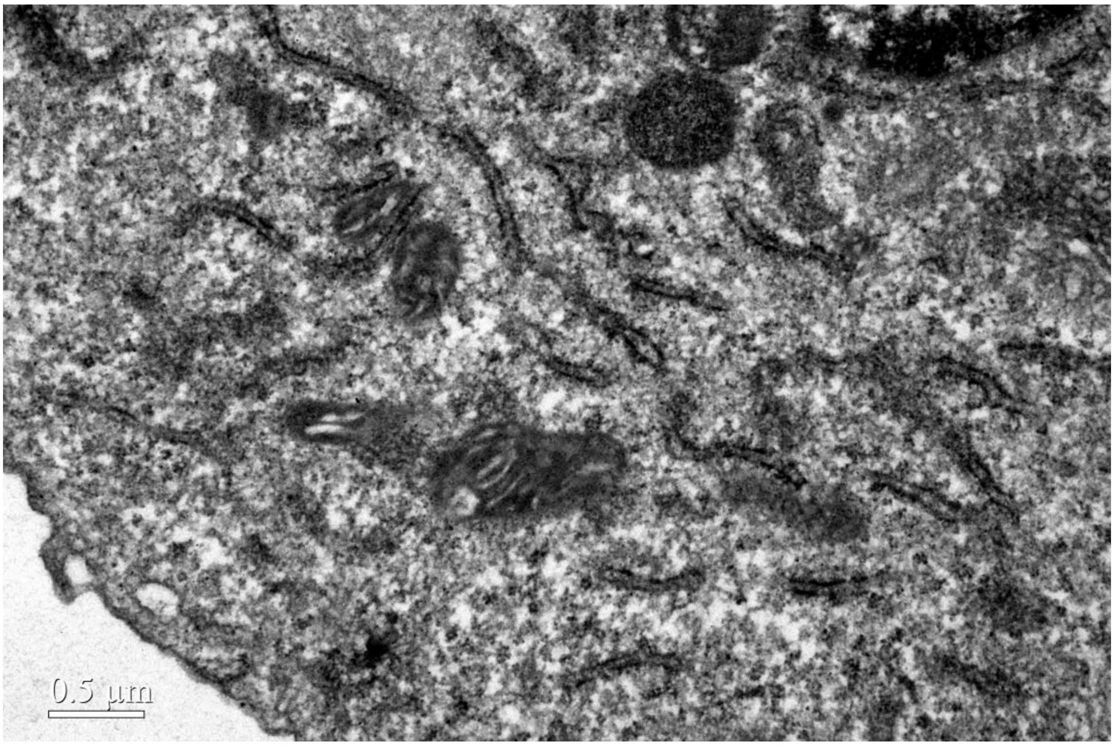

Figure S4 A the original microscopy images displayed in **Figure4 G**
