# Supplementary material for: PIEZO1 Ion Channel Mediates Ionizing Radiation-Induced Pulmonary Endothelial Cell Ferroptosis via Ca2+/Calpain/VE-Cadherin Signaling
Source: Front Mol Biosci. 2021 Sep 9;8:725274. doi: 10.3389/fmolb.2021.725274 (PMC8458942; doi:10.3389/fmolb.2021.725274)
Supplement: Supplementary file 3 [file Table1.DOCX]

**Figure1 A**

**Figure1 B**

**Figure2 A**

**Figure2 B**

**Figure3 D**

**Figure3 E**

**Figure3 F**

**Figure3 G**

**Figure4 E**

**Figure4 F**

**Figure4 H**

**Figure4 I**
