# Supplementary material for: PIEZO1 Ion Channel Mediates Ionizing Radiation-Induced Pulmonary Endothelial Cell Ferroptosis via Ca2+/Calpain/VE-Cadherin Signaling
Source: Front Mol Biosci. 2021 Sep 9;8:725274. doi: 10.3389/fmolb.2021.725274 (PMC8458942; doi:10.3389/fmolb.2021.725274)

Supplementary Information includes:source data (excel)
flow cytometry data (fcs)
microscopy images (pdf)
fscans of the entire original gels(pdf)

Figure S1 A

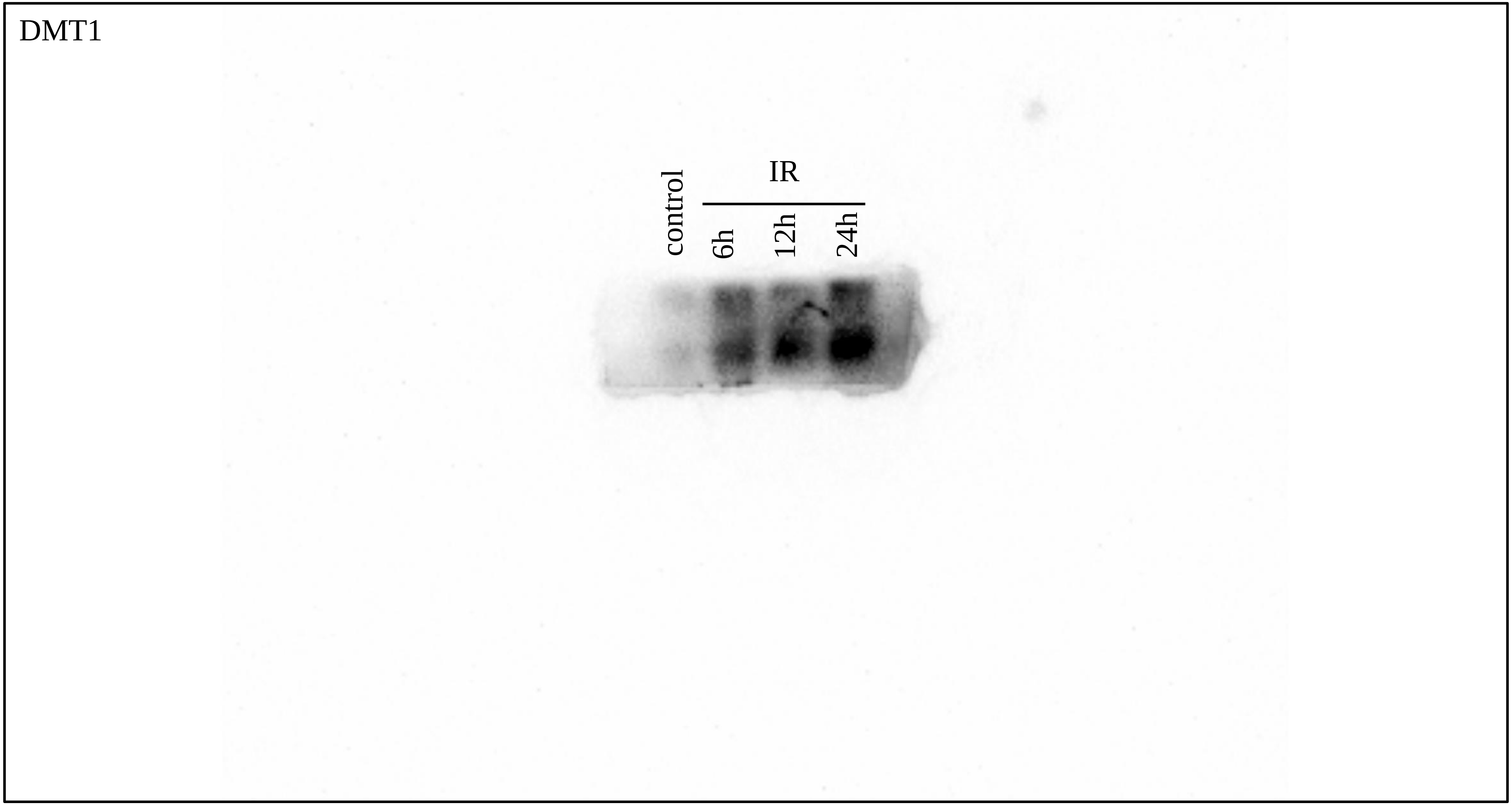

Figure S1 B

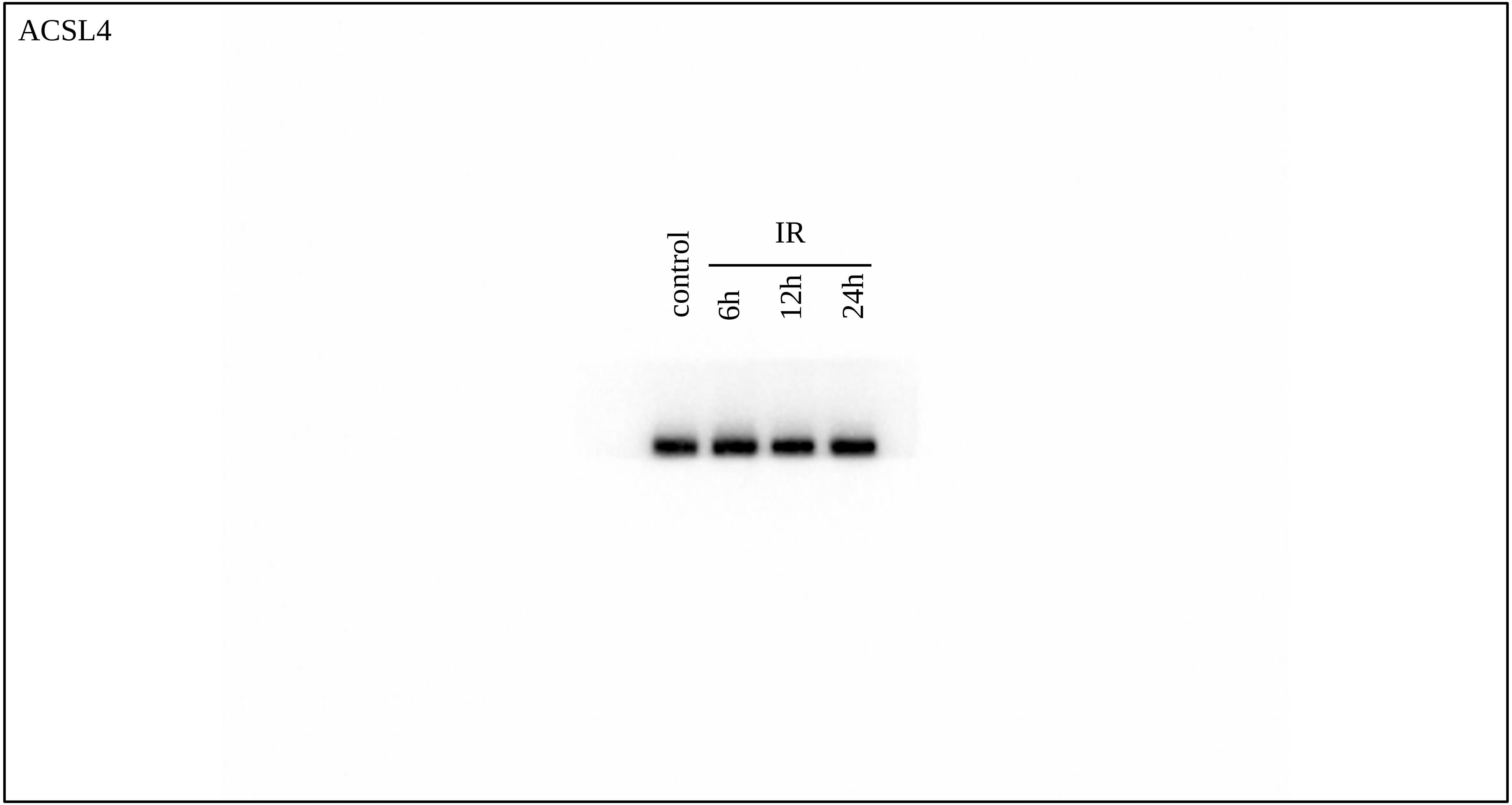

Figure S1 C

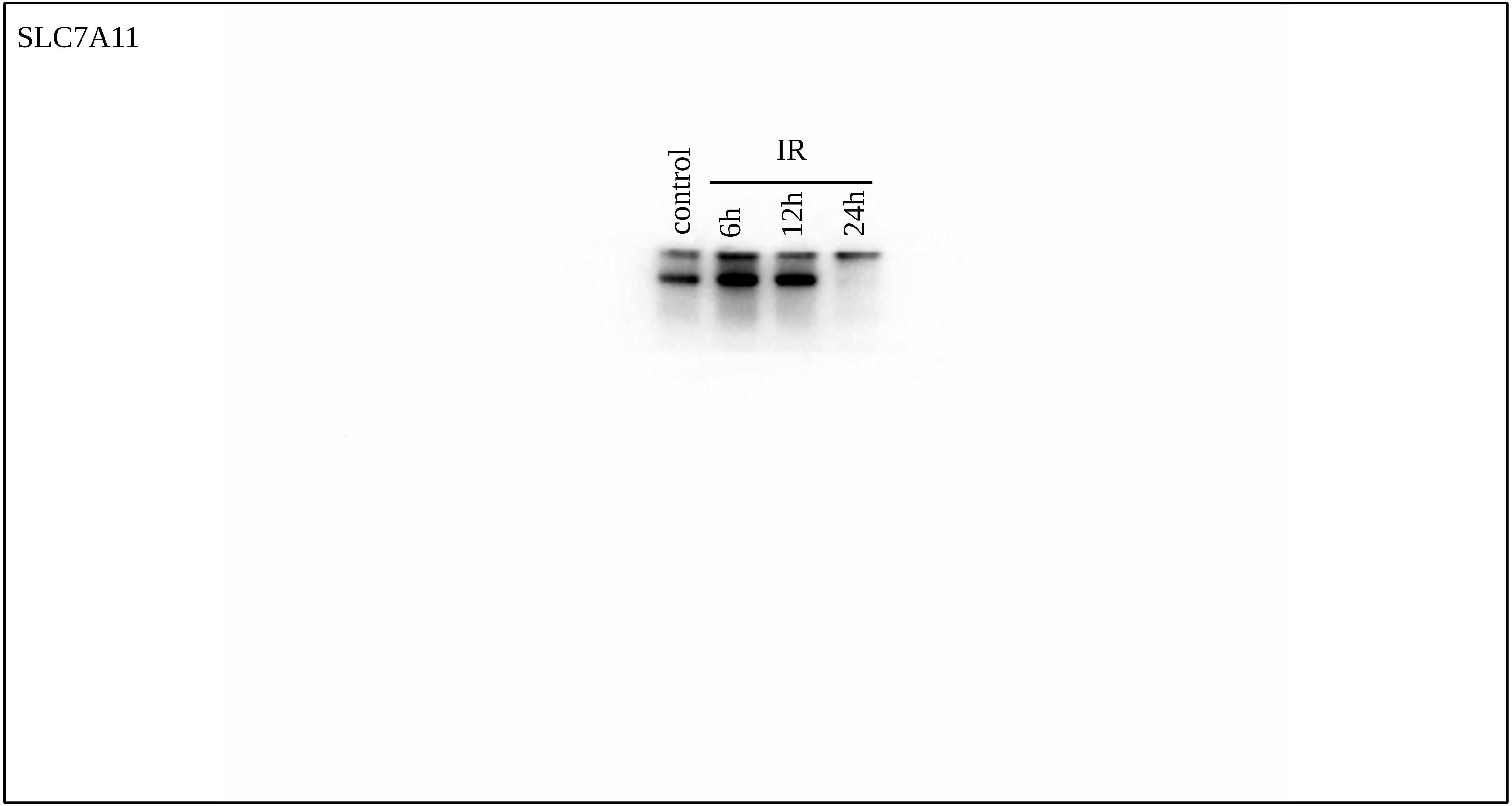

Figure S1 D

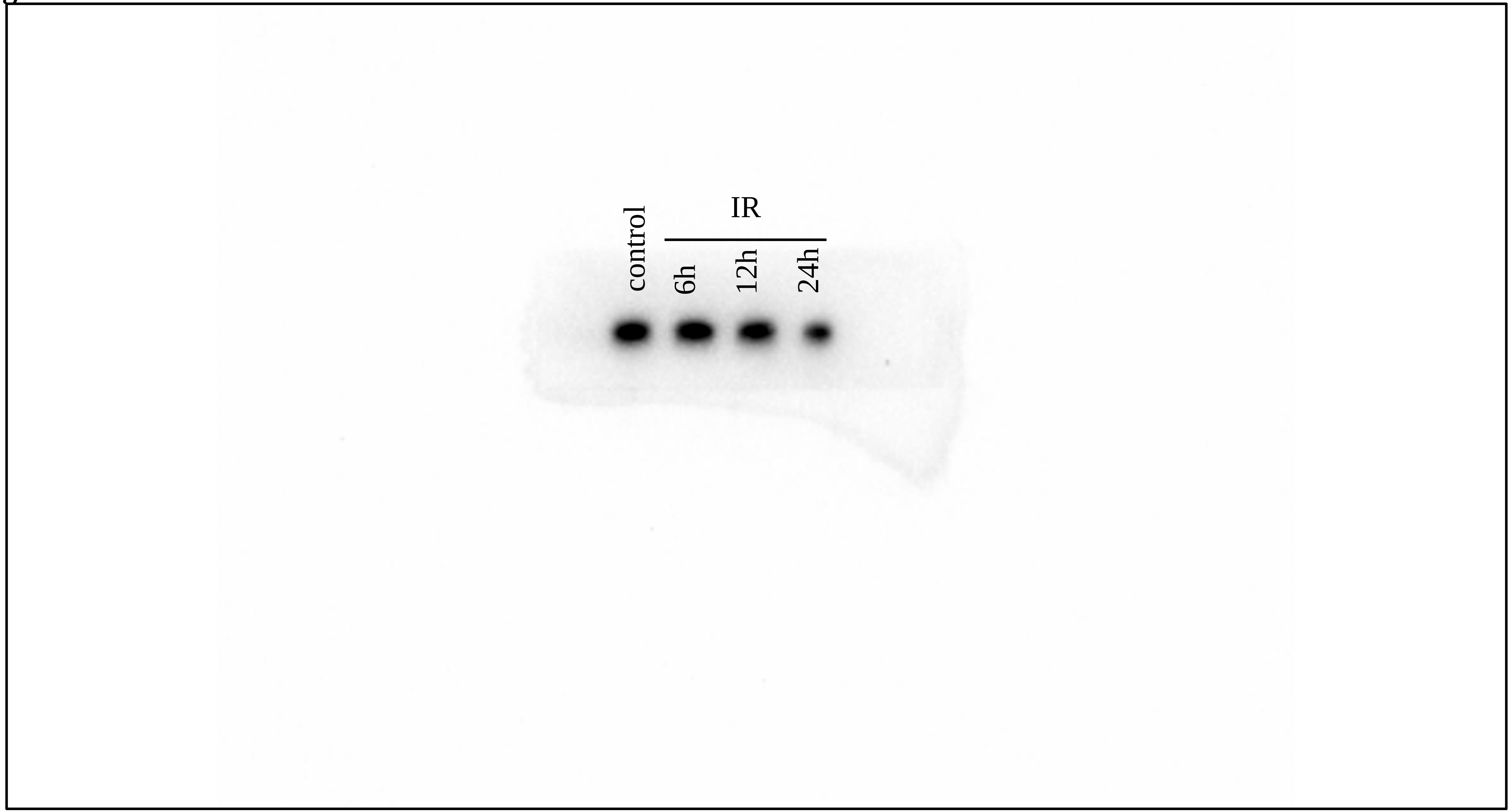

Figure S1 E

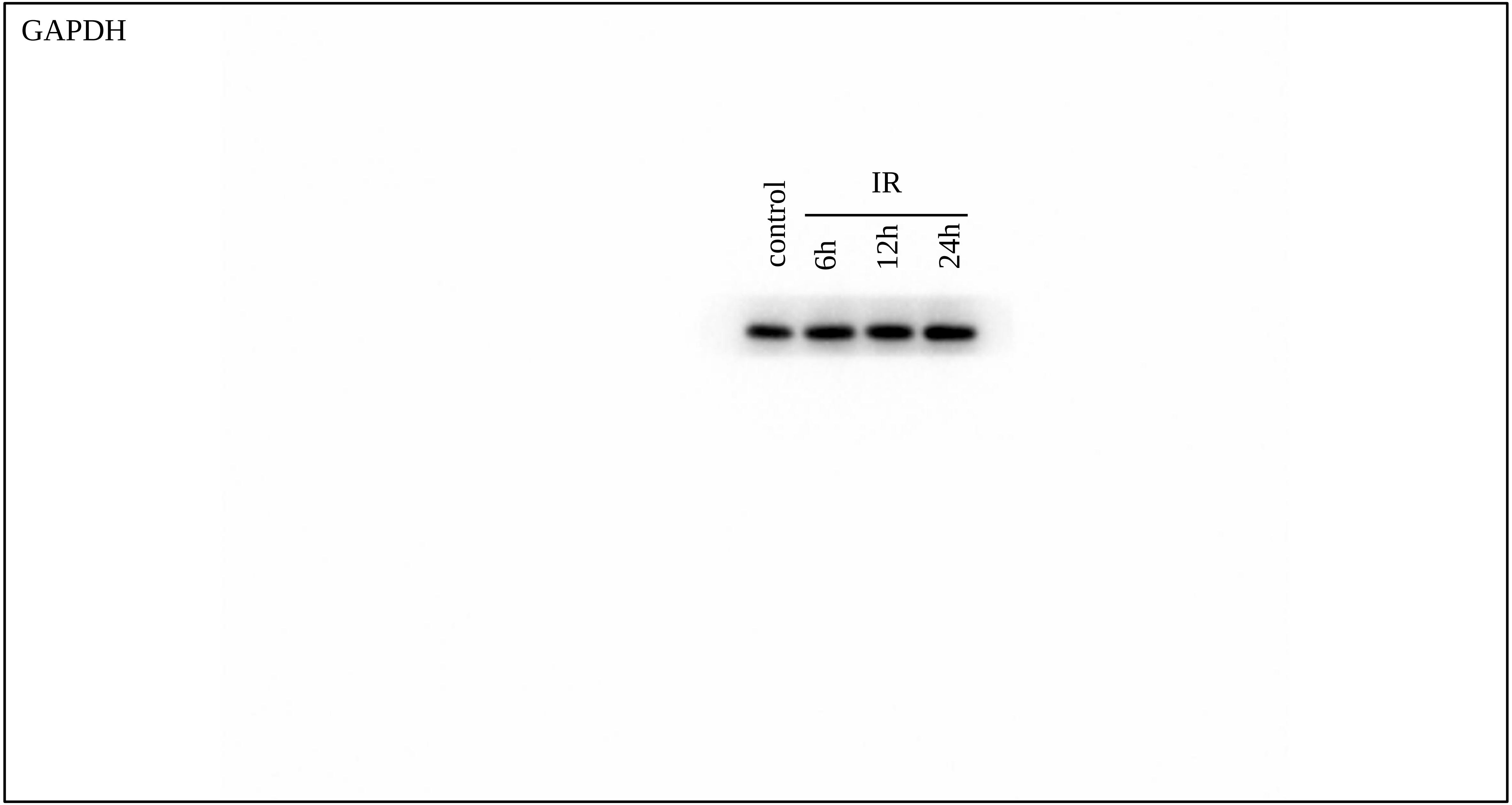

Figure S1 A-E Full scans of the entire original gels displayed in **Figure1 C**

Figure S2 A

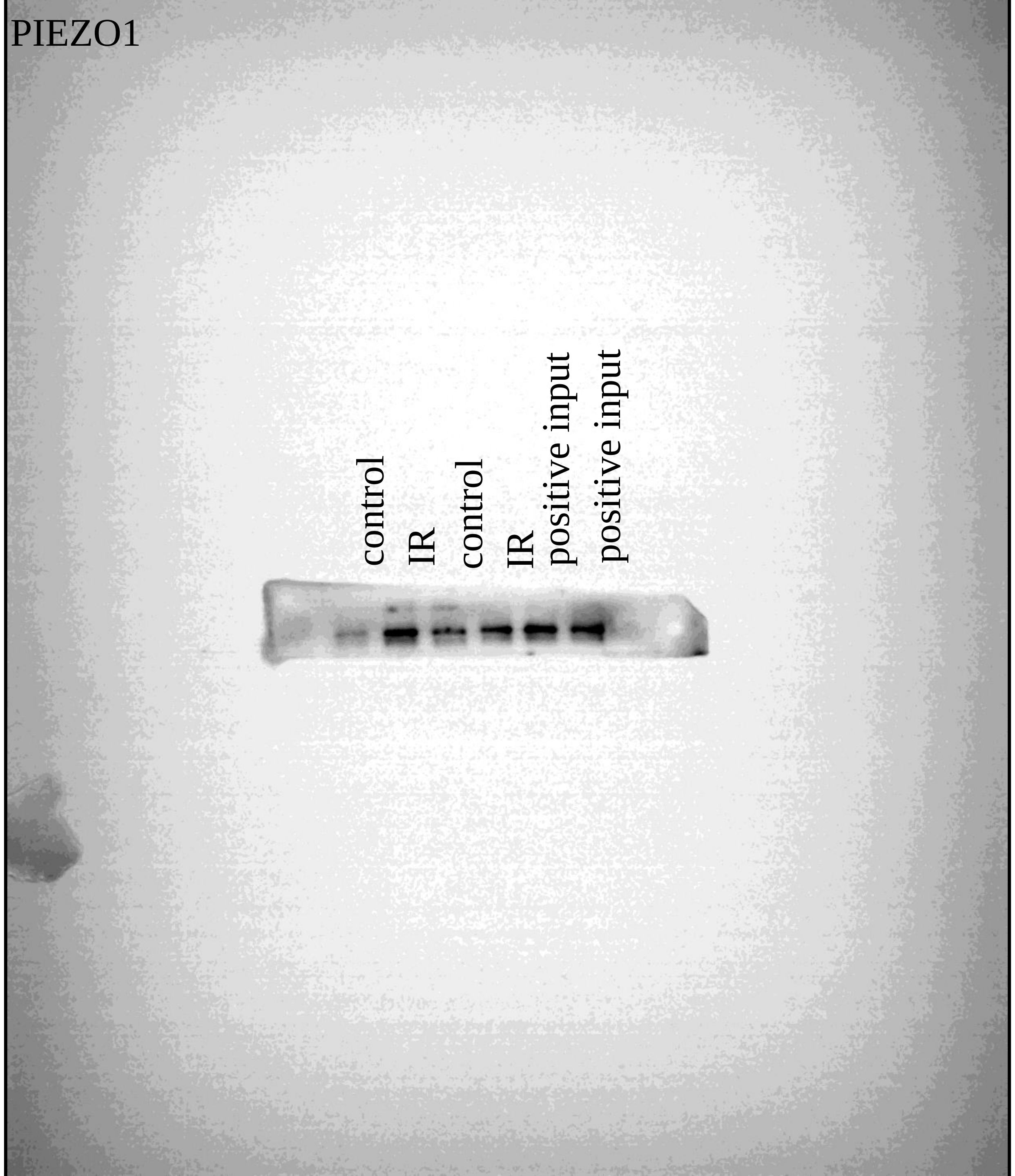
PIEZO1
control
IR
control
IR
positive input
positive input

Figure S2 b

α-Tubulin

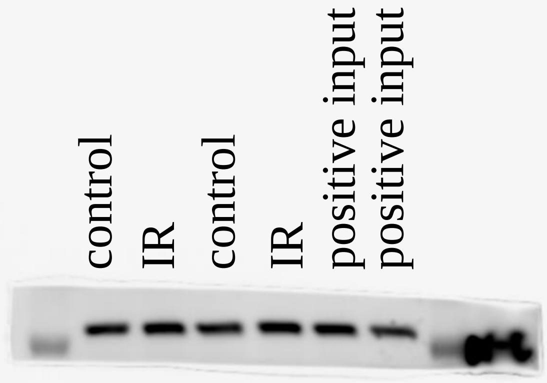

Figure S2 A-B Full scans of the entire original gels displayed in **Figure1 F**

Figure S3 A

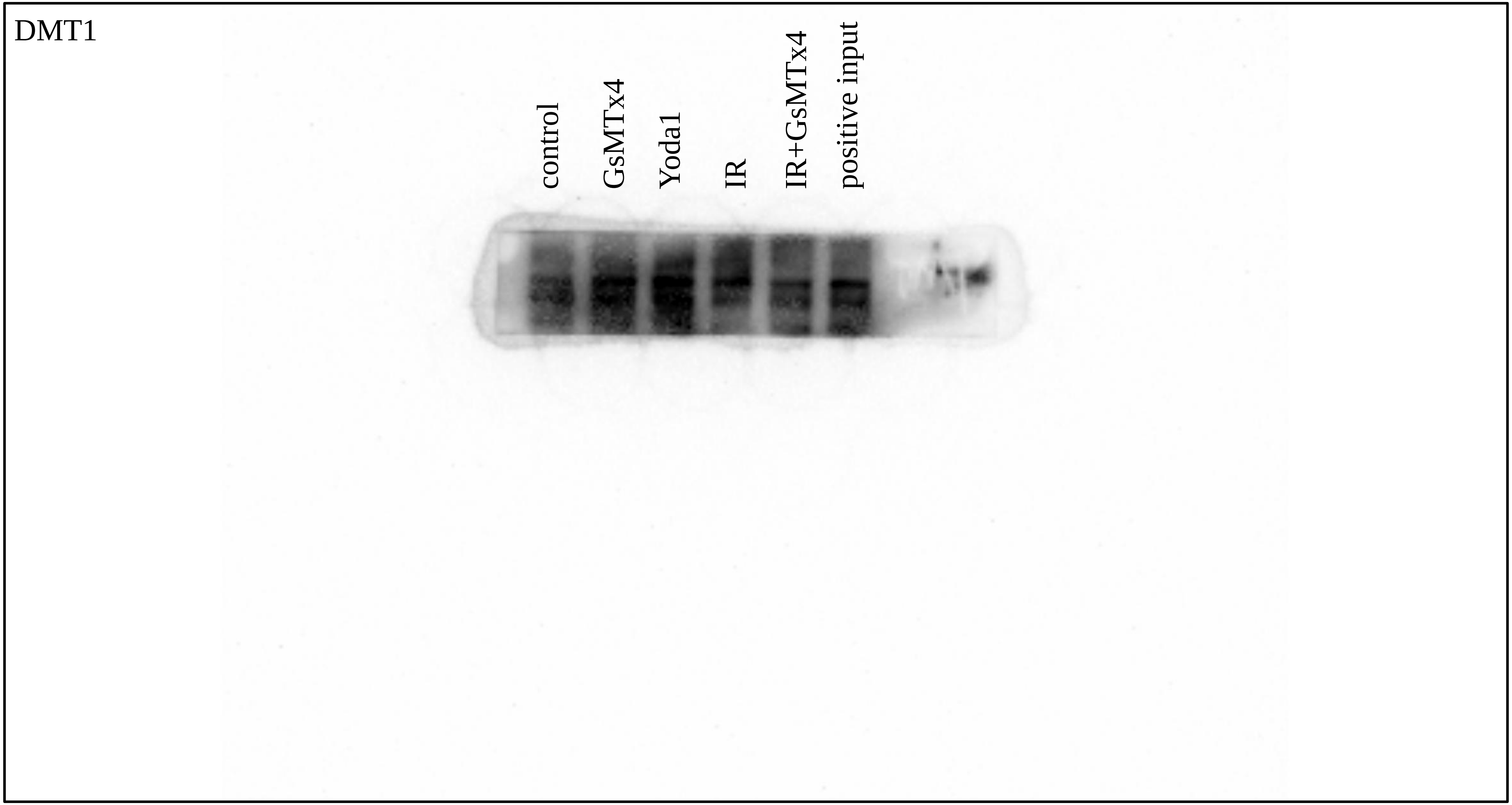

Figure S3 B

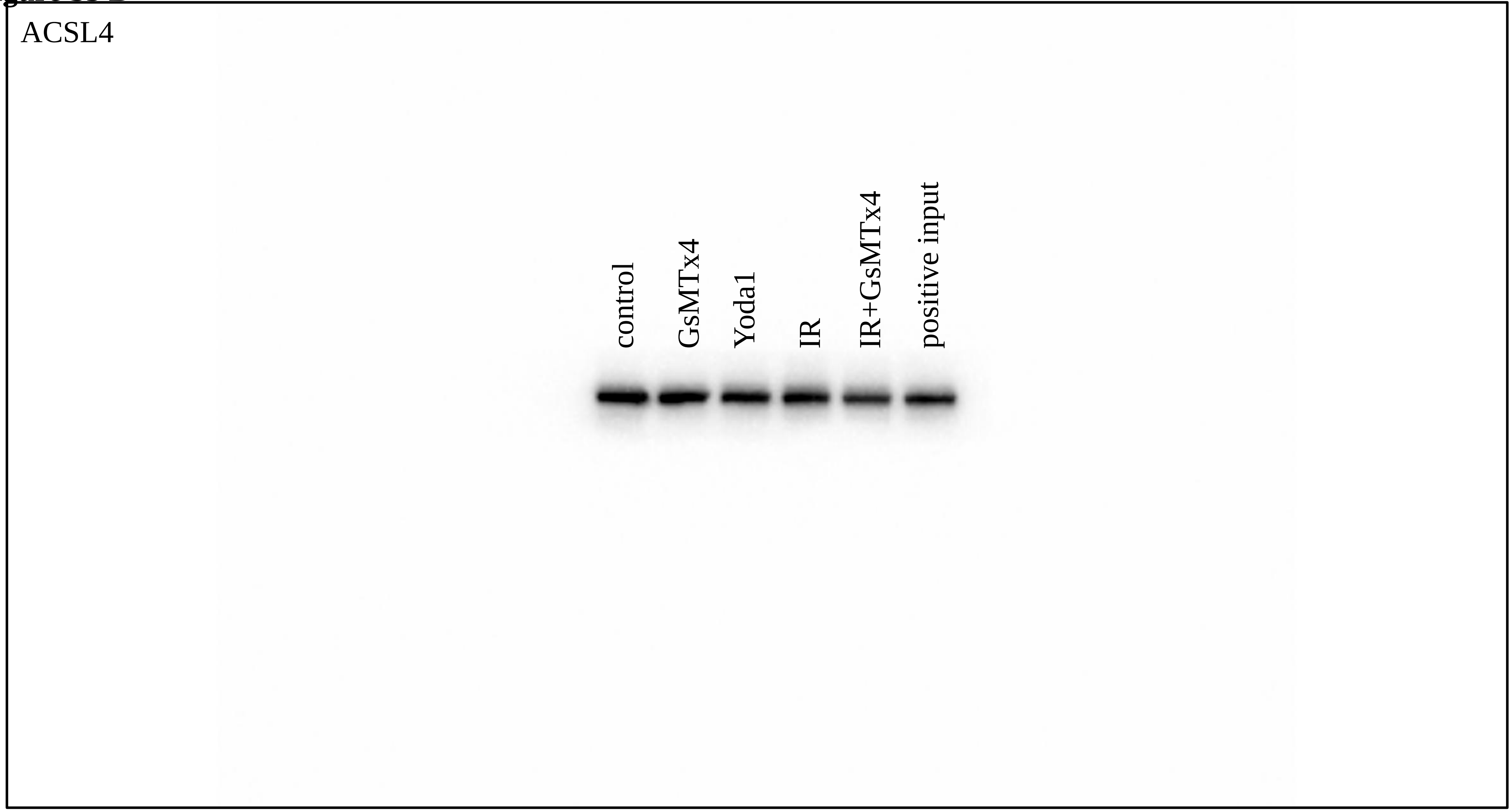

Figure S3 C

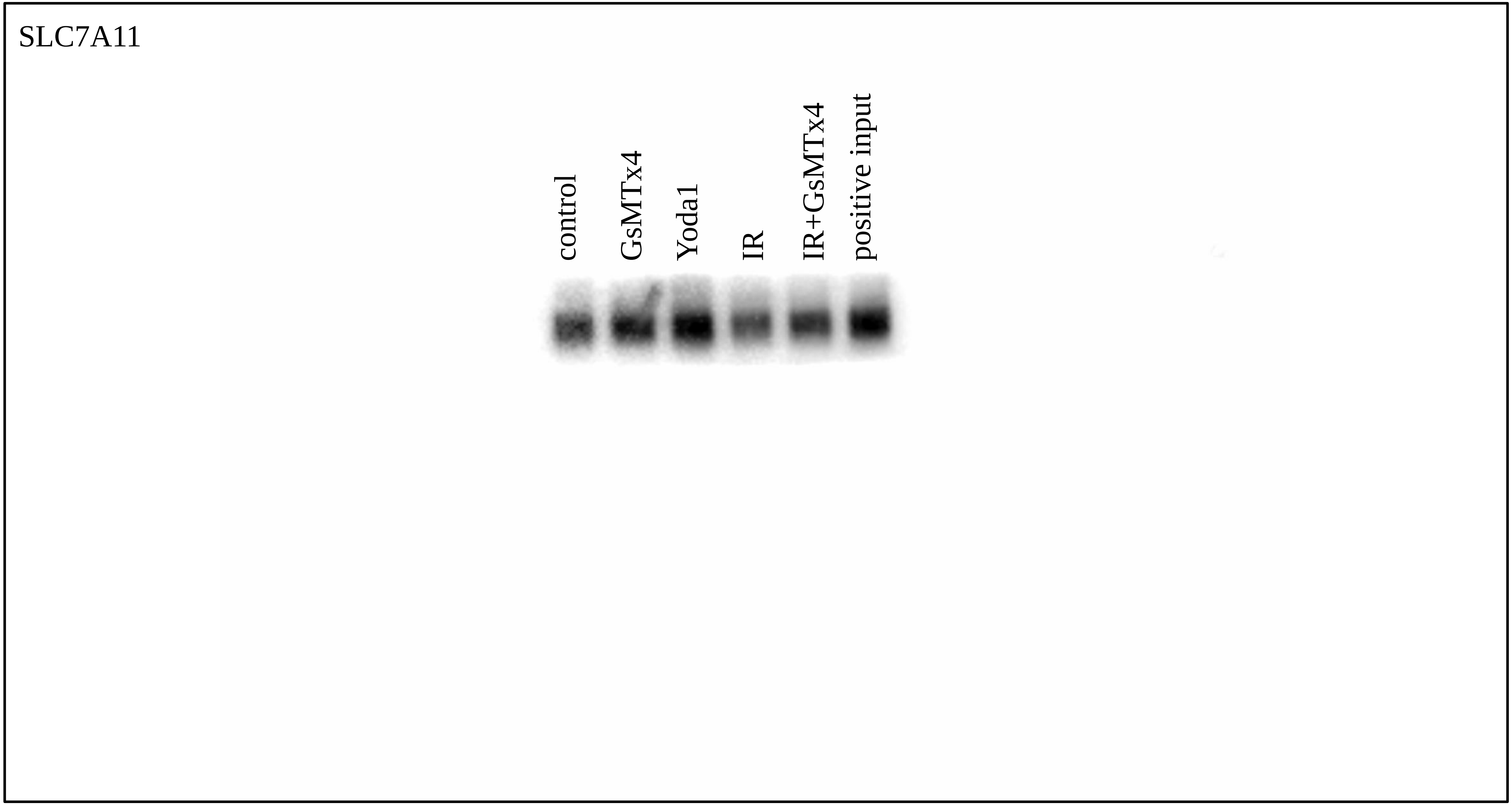

Figure S3 D

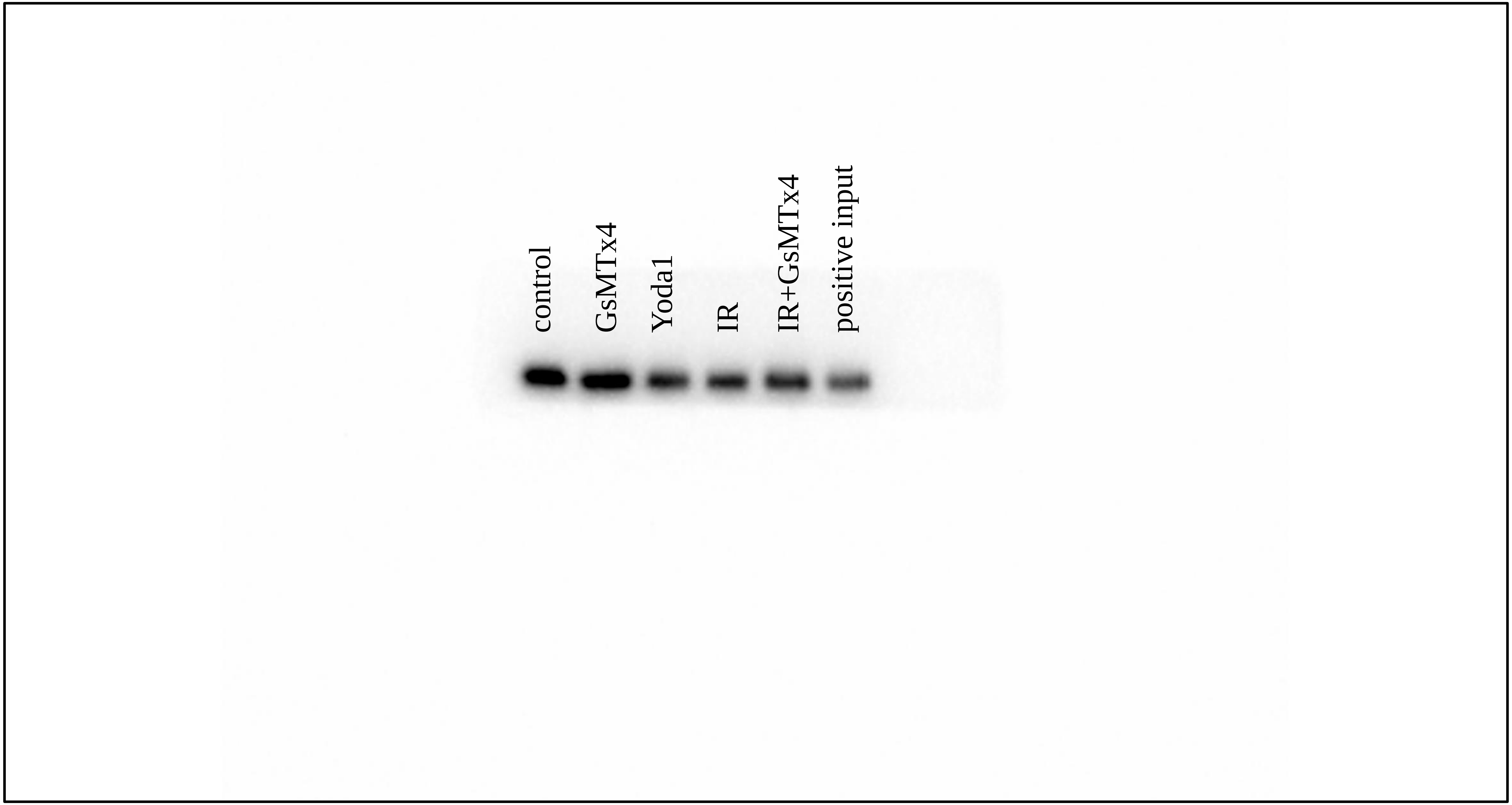

Figure S3 E

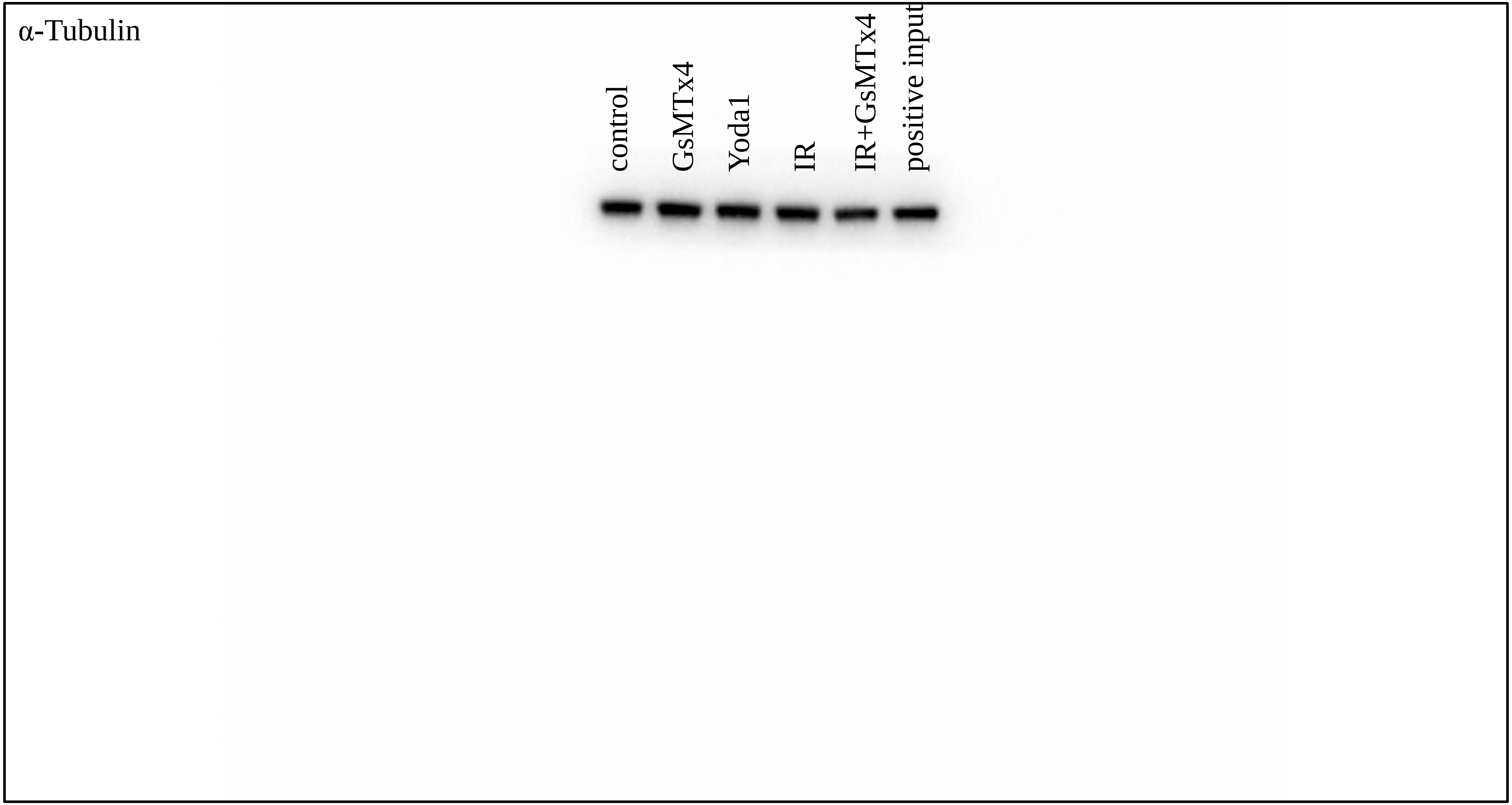

Figure S3 A-E Full scans of the entire original gels displayed in **Figure2 C**

Figure S4 A

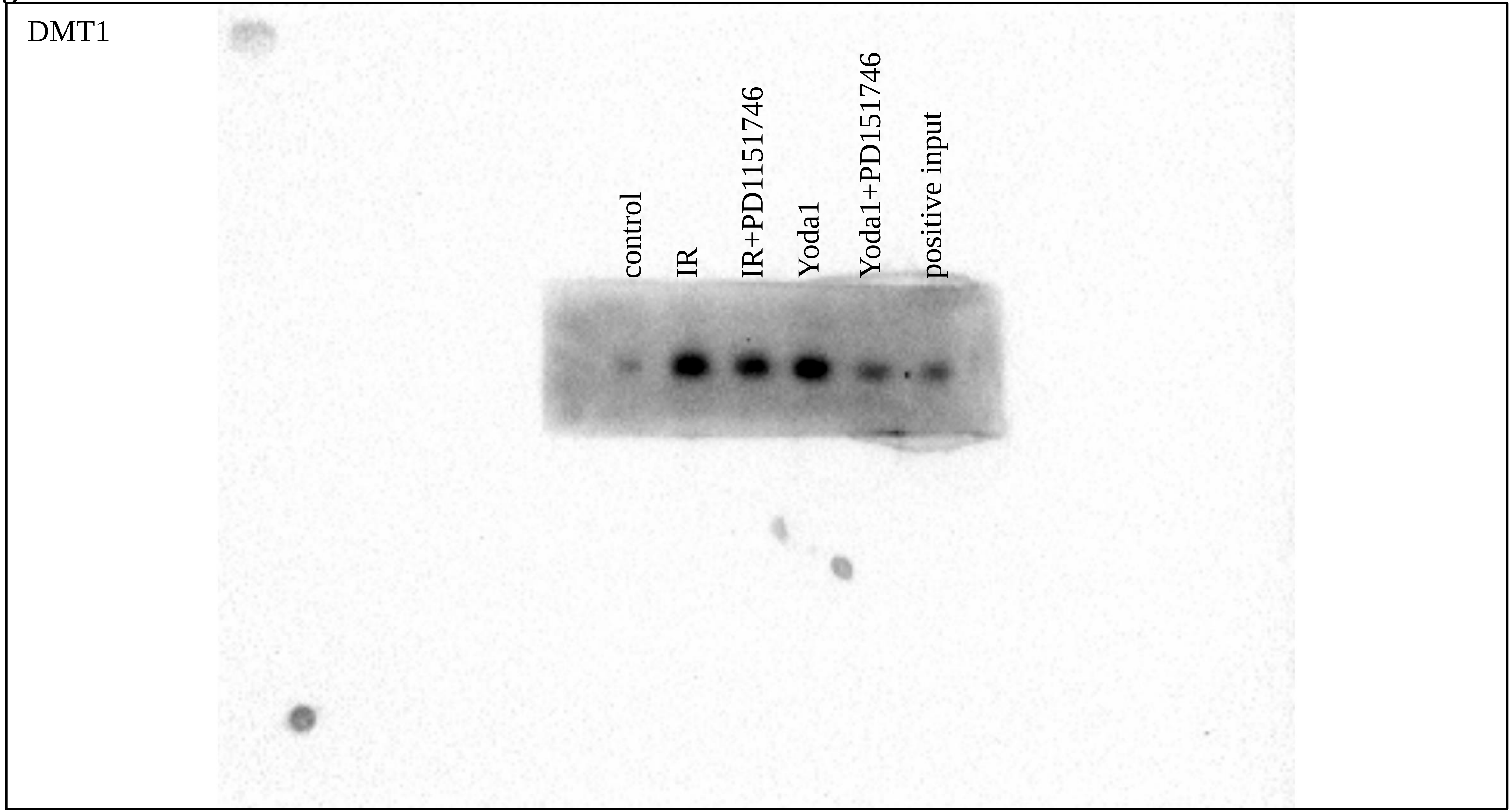
DMT1
control
IR
IR+PD1151746
Yoda1
Yoda1+PD151746
positive input

Figure S4 B

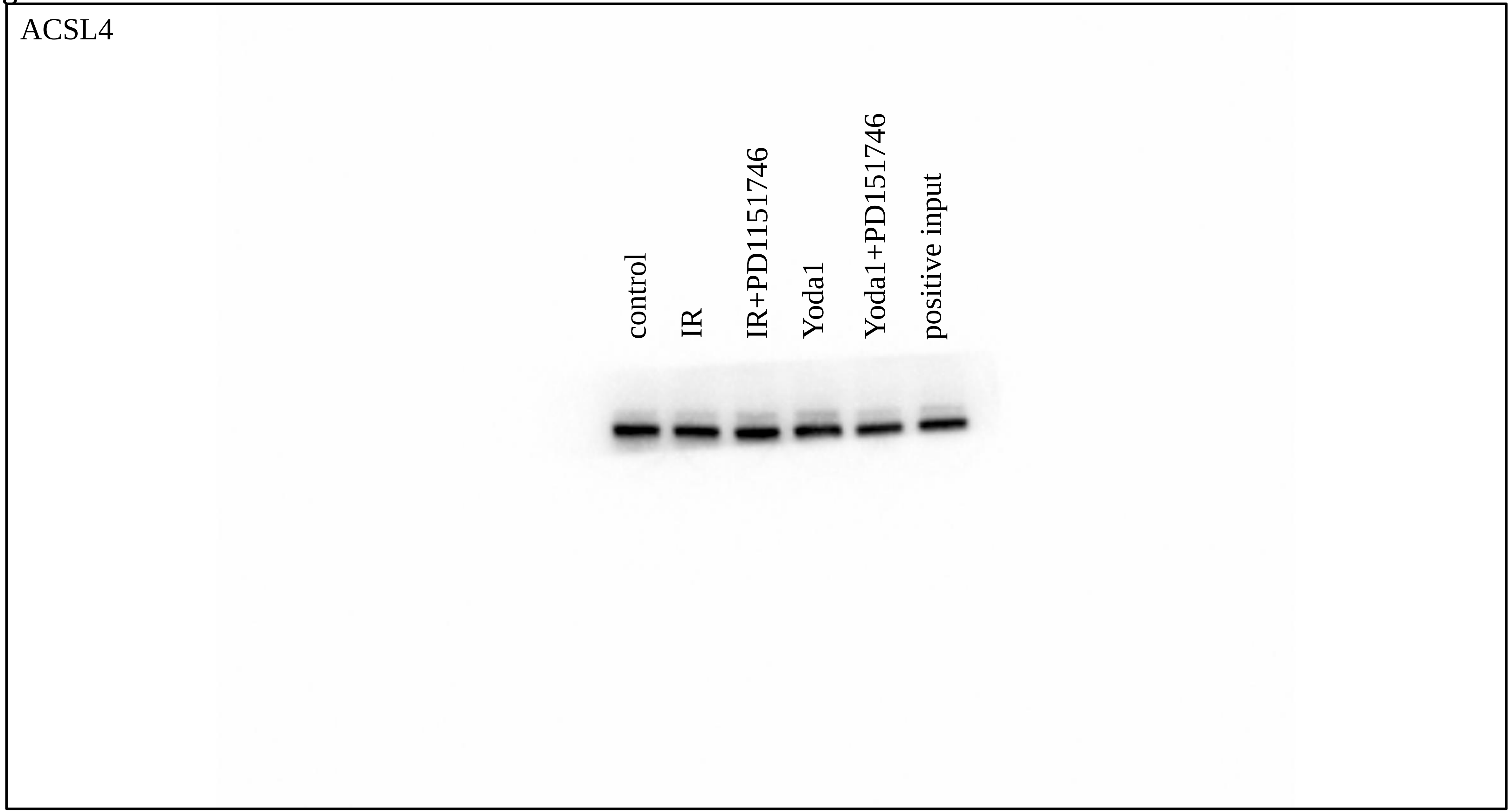
ACSL4
control
IR
IR+PD1151746
Yoda1
Yoda1+PD151746
positive input

Figure S4 C

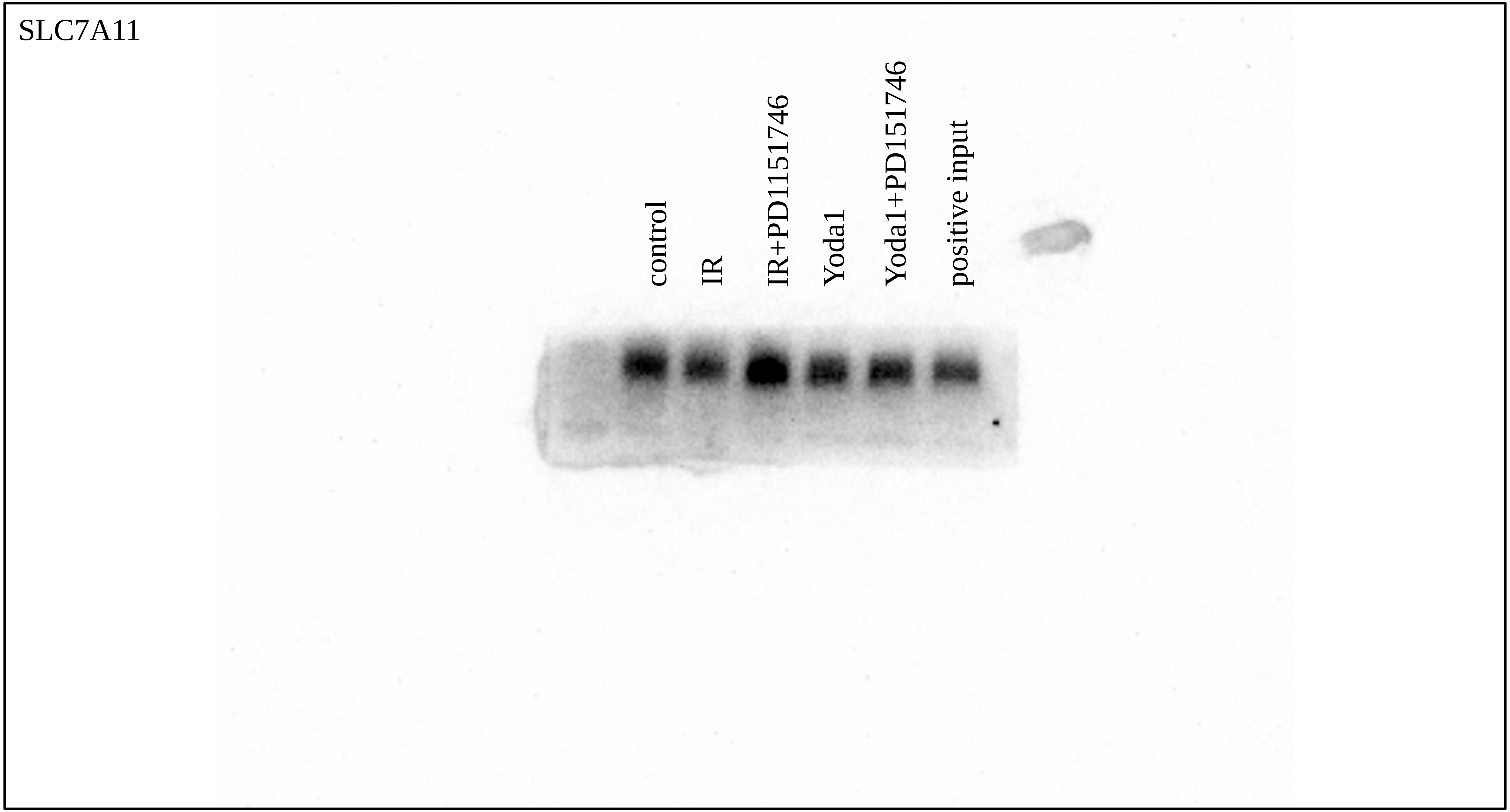
SLC7A11
control
IR
IR+PD1151746
Yoda1
Yoda1+PD151746
positive input

Figure S4 D

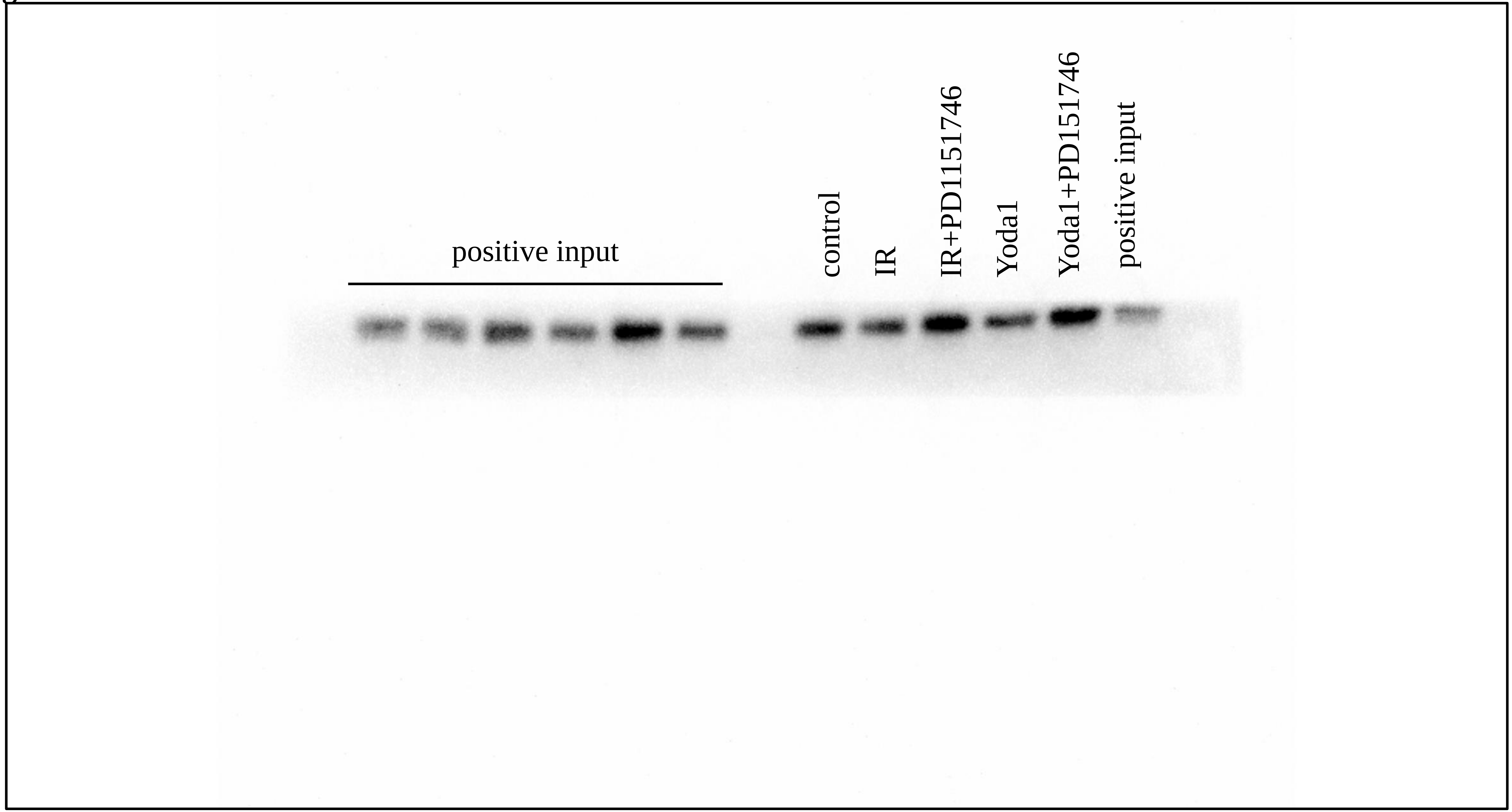
positive input
control
IR
IR+PD1151746
Yoda1
Yoda1+PD151746
positive input

Figure S4 E

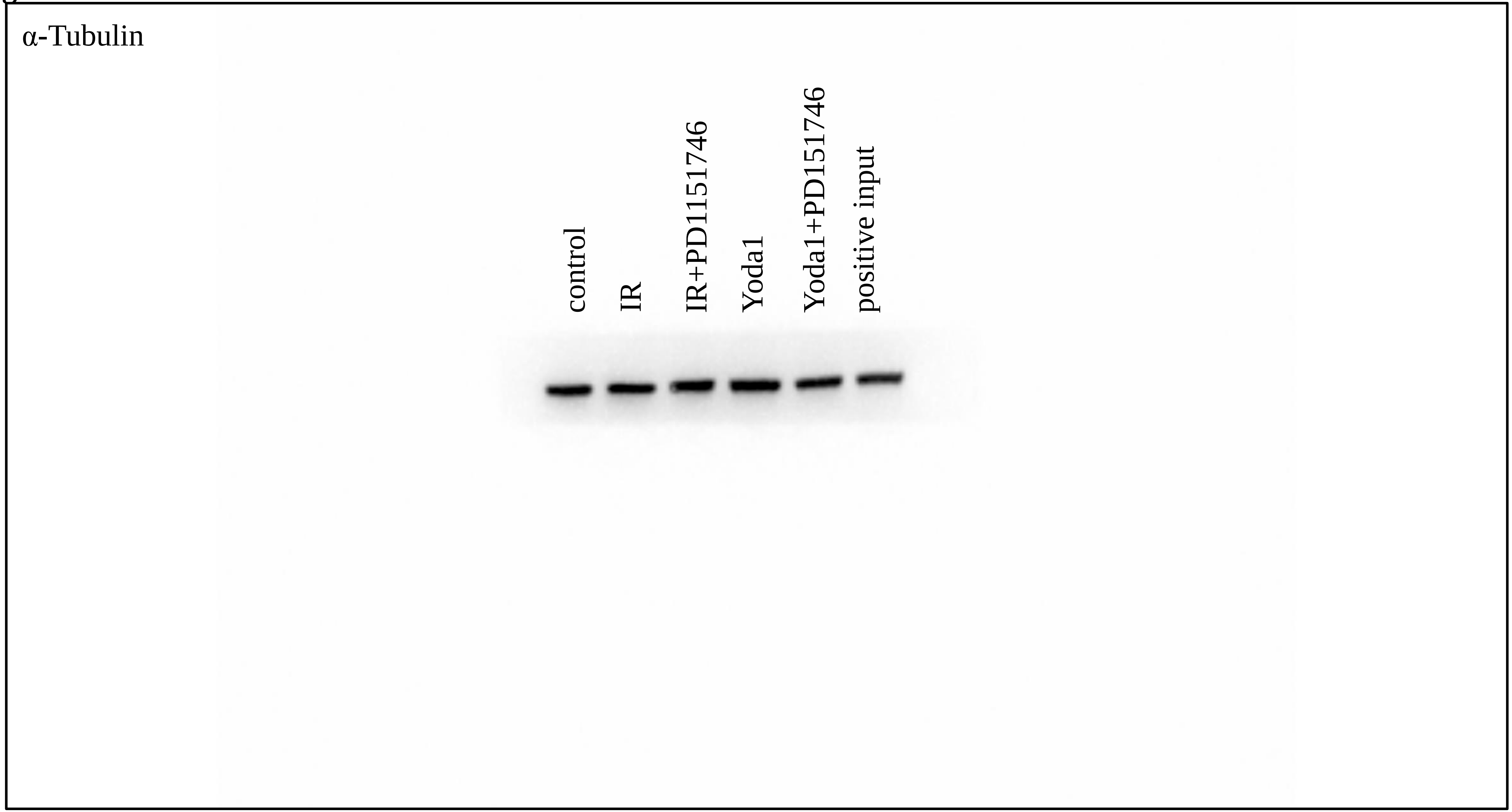

α-Tubulin
control
IR
IR+PD1151746
Yoda1
Yoda1+PD151746
positive input

Figure S4 A-E Full scans of the entire original gels displayed in **Figure3 H**

Figure S5 A

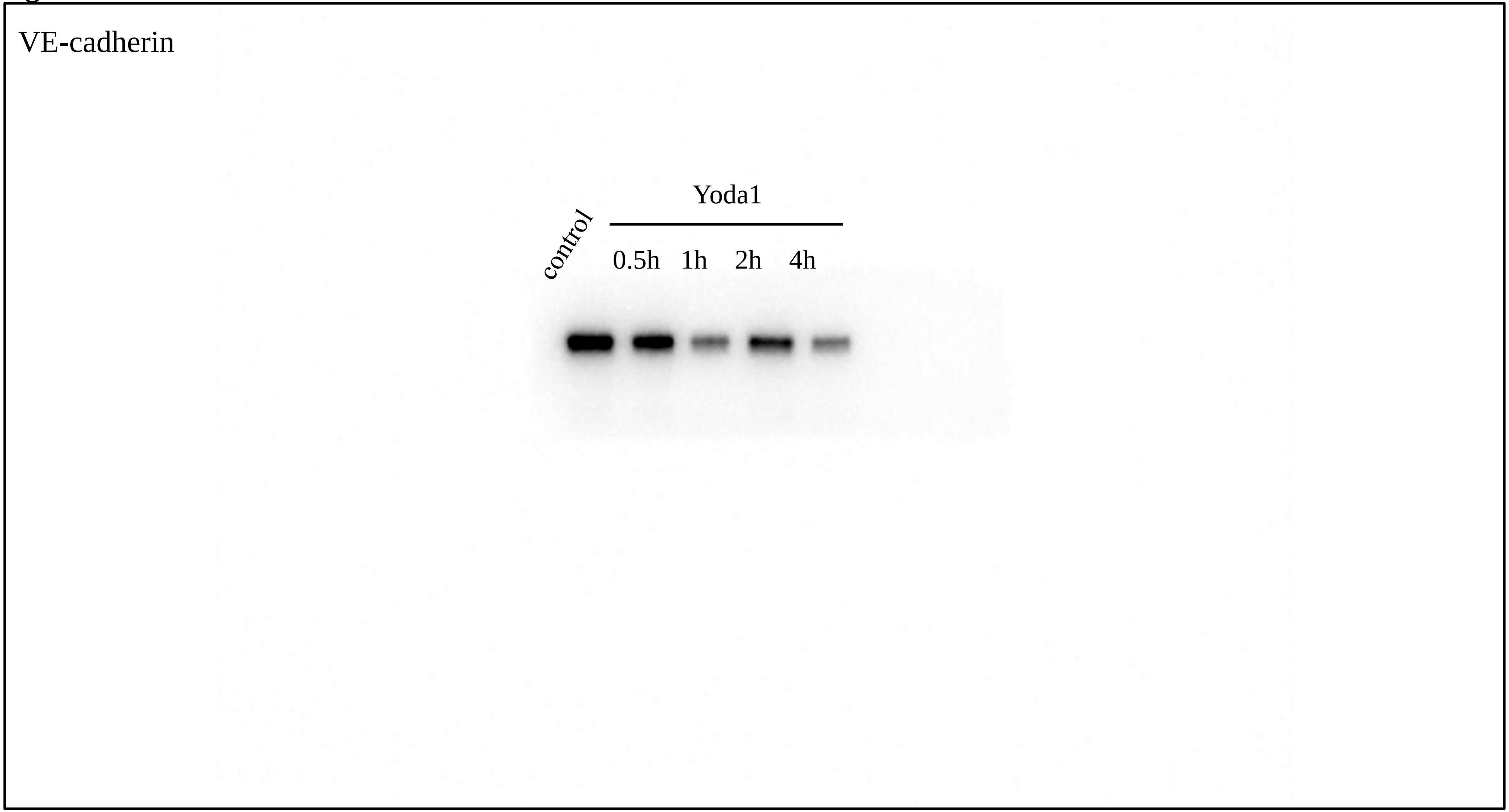

Figure S5 B

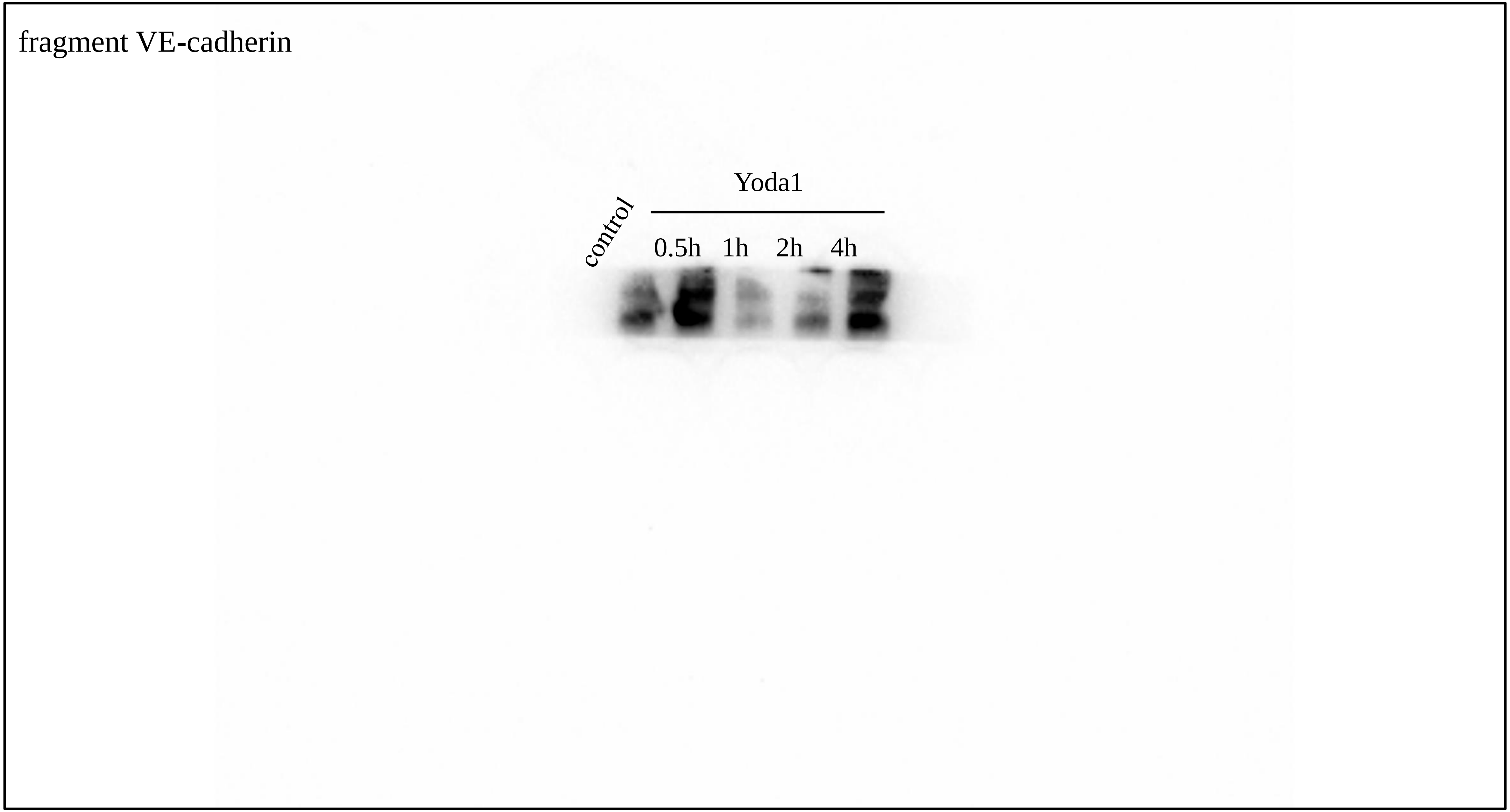

Figure S5 C

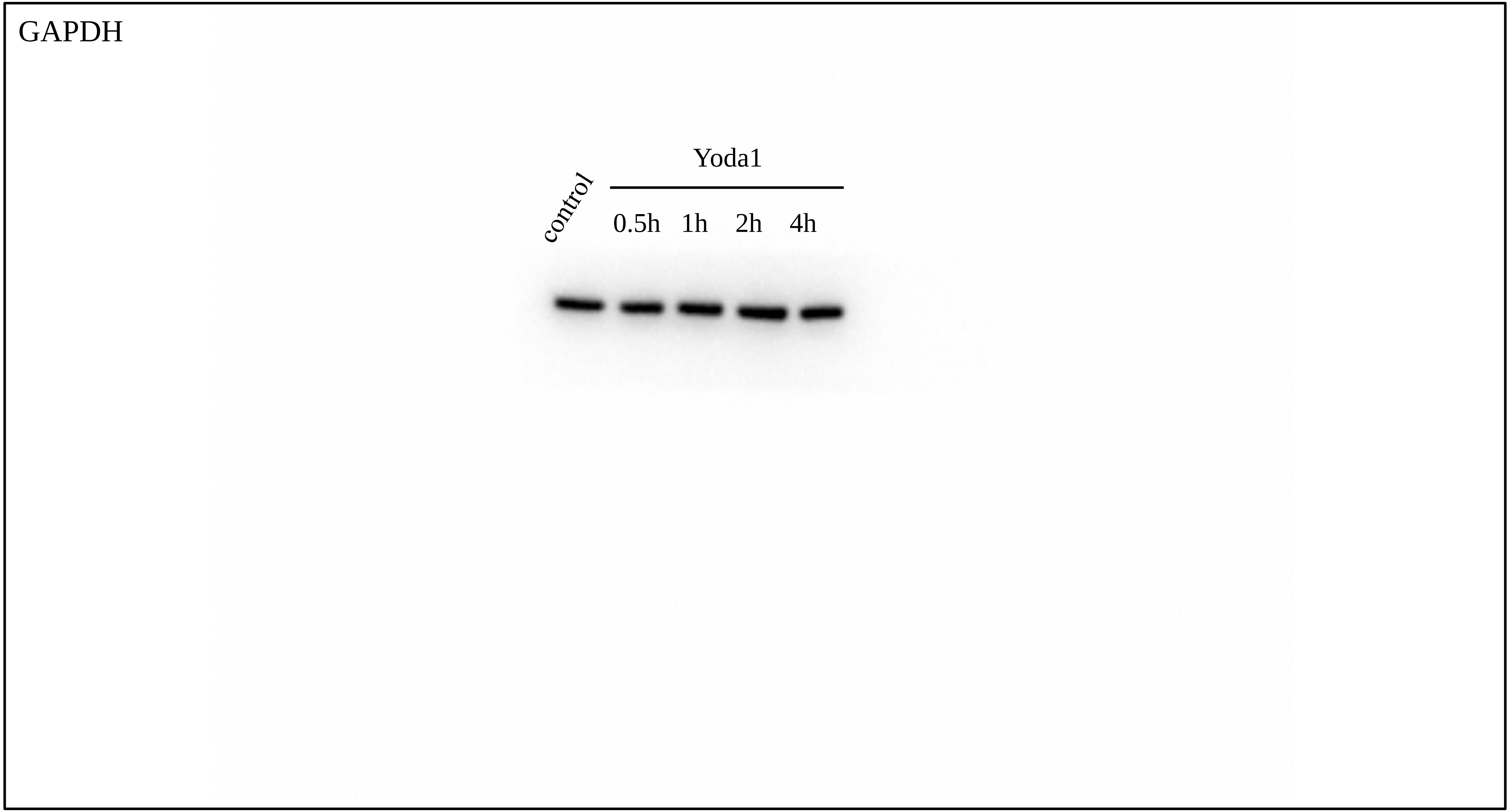

Figure S5 A-C Full scans of the entire original gels displayed in **Figure4 B**

Figure S6 A

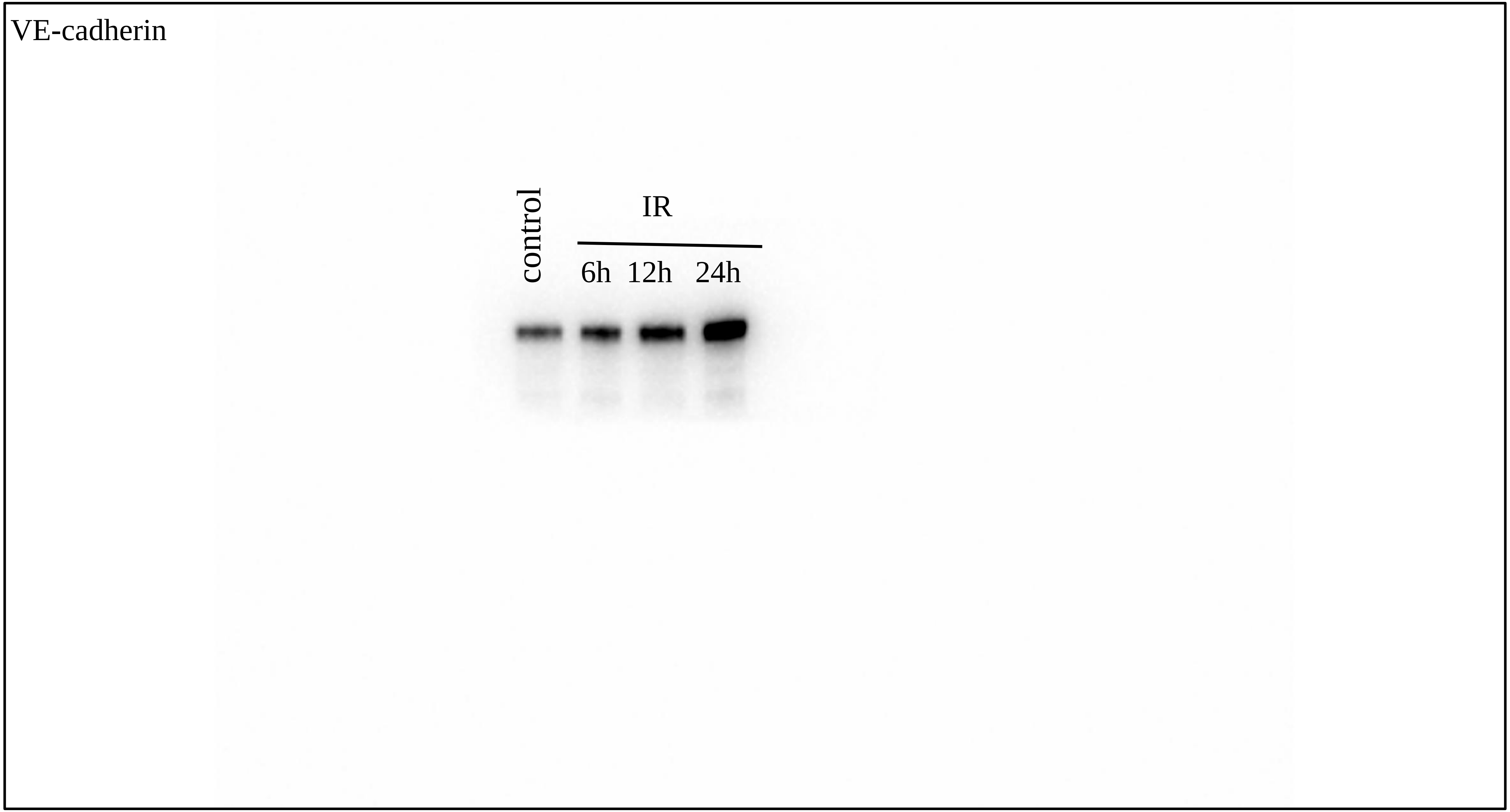

Figure S6 B

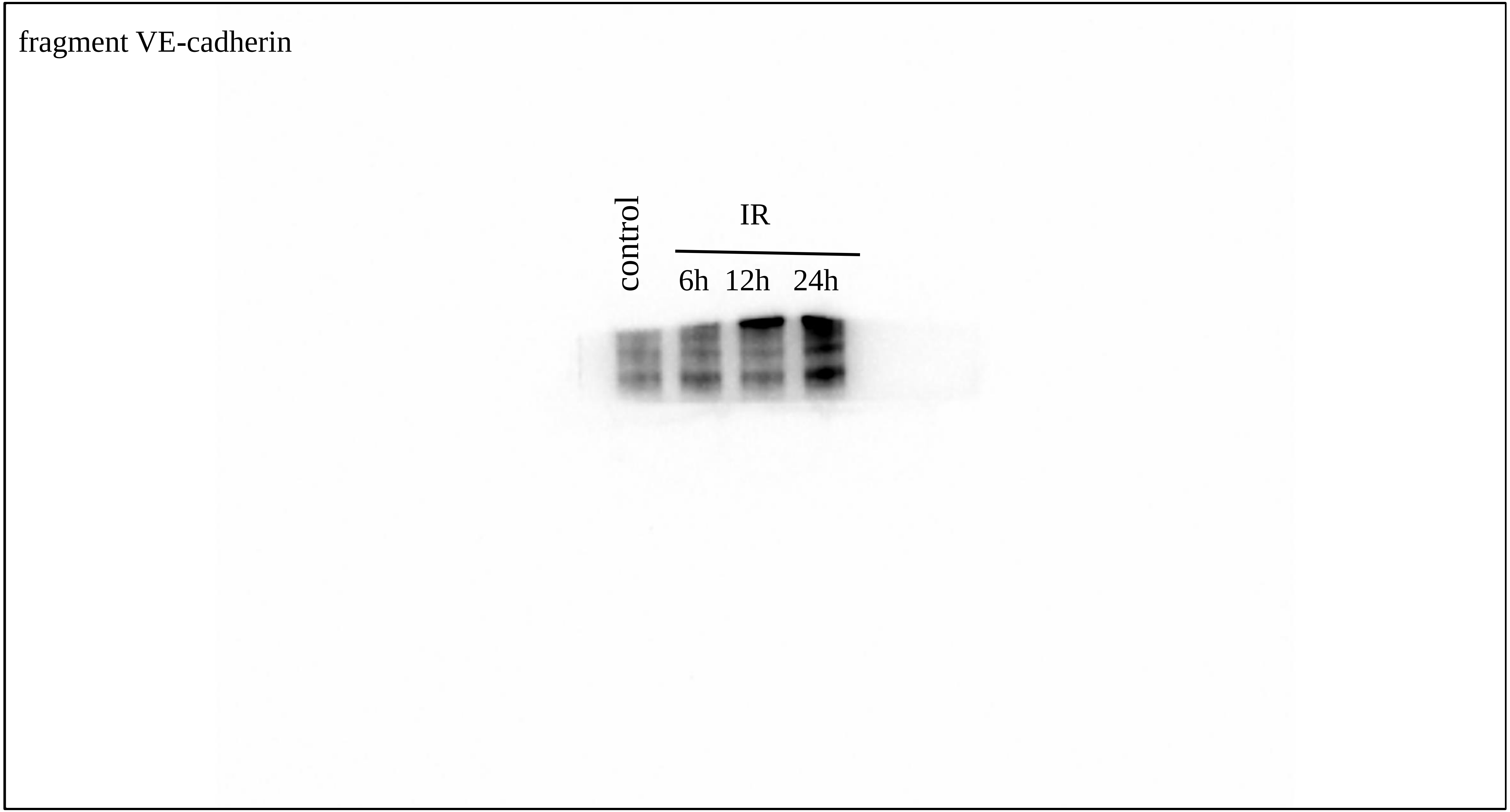

Figure S6 C

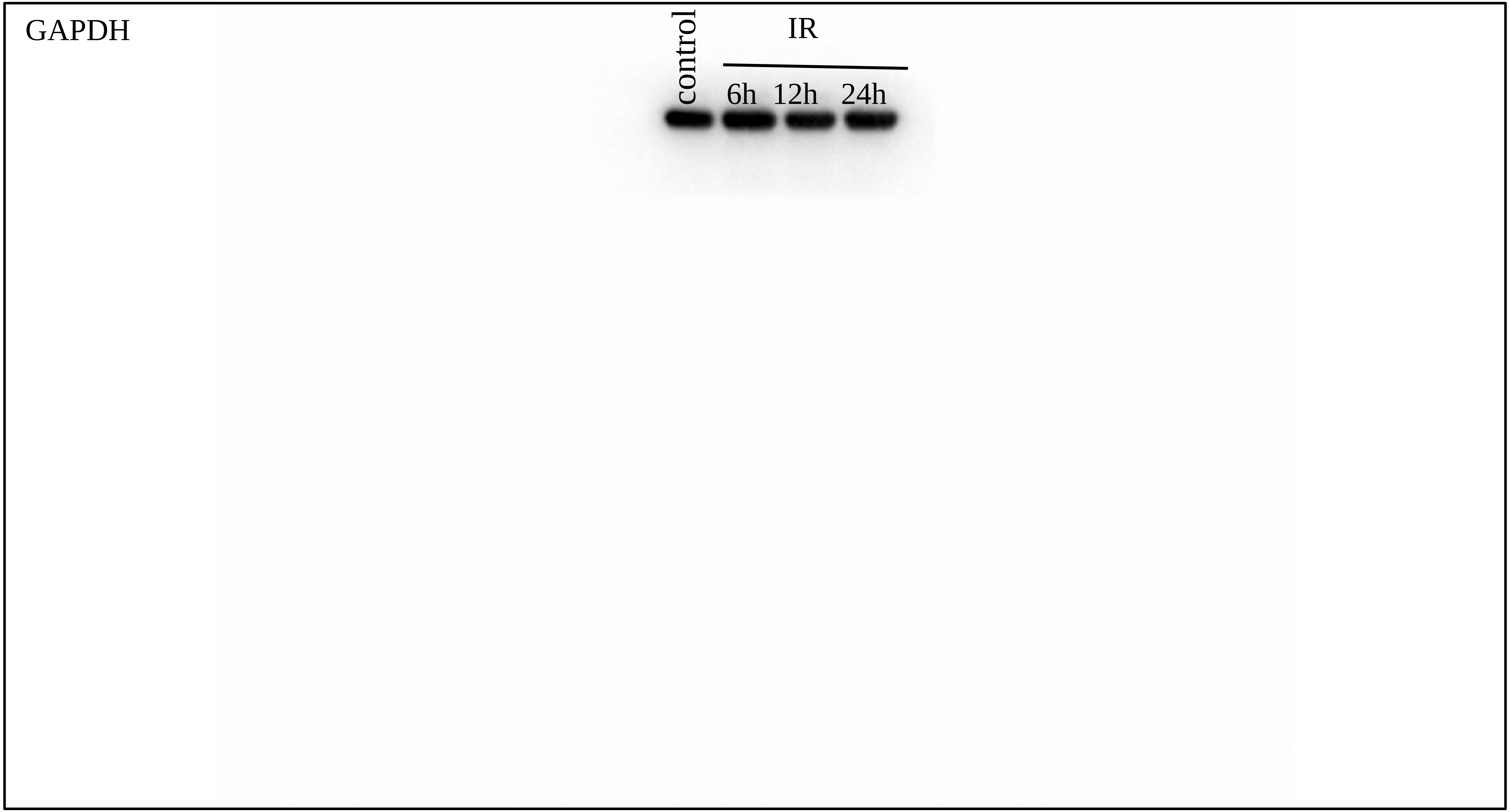

Figure S6 A-C Full scans of the entire original gels displayed in **Figure4 C**

Figure S7 A

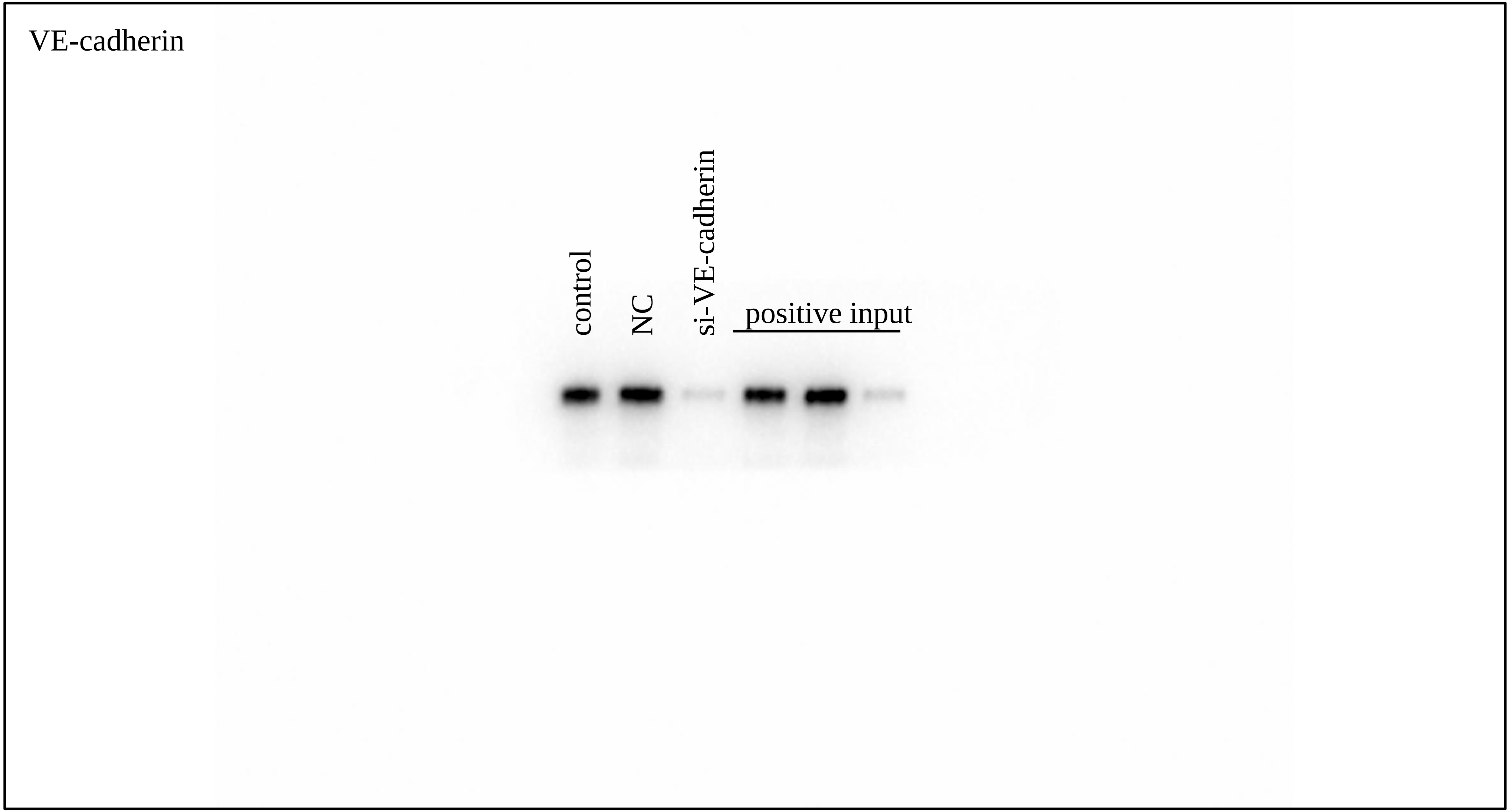

Figure S7 B

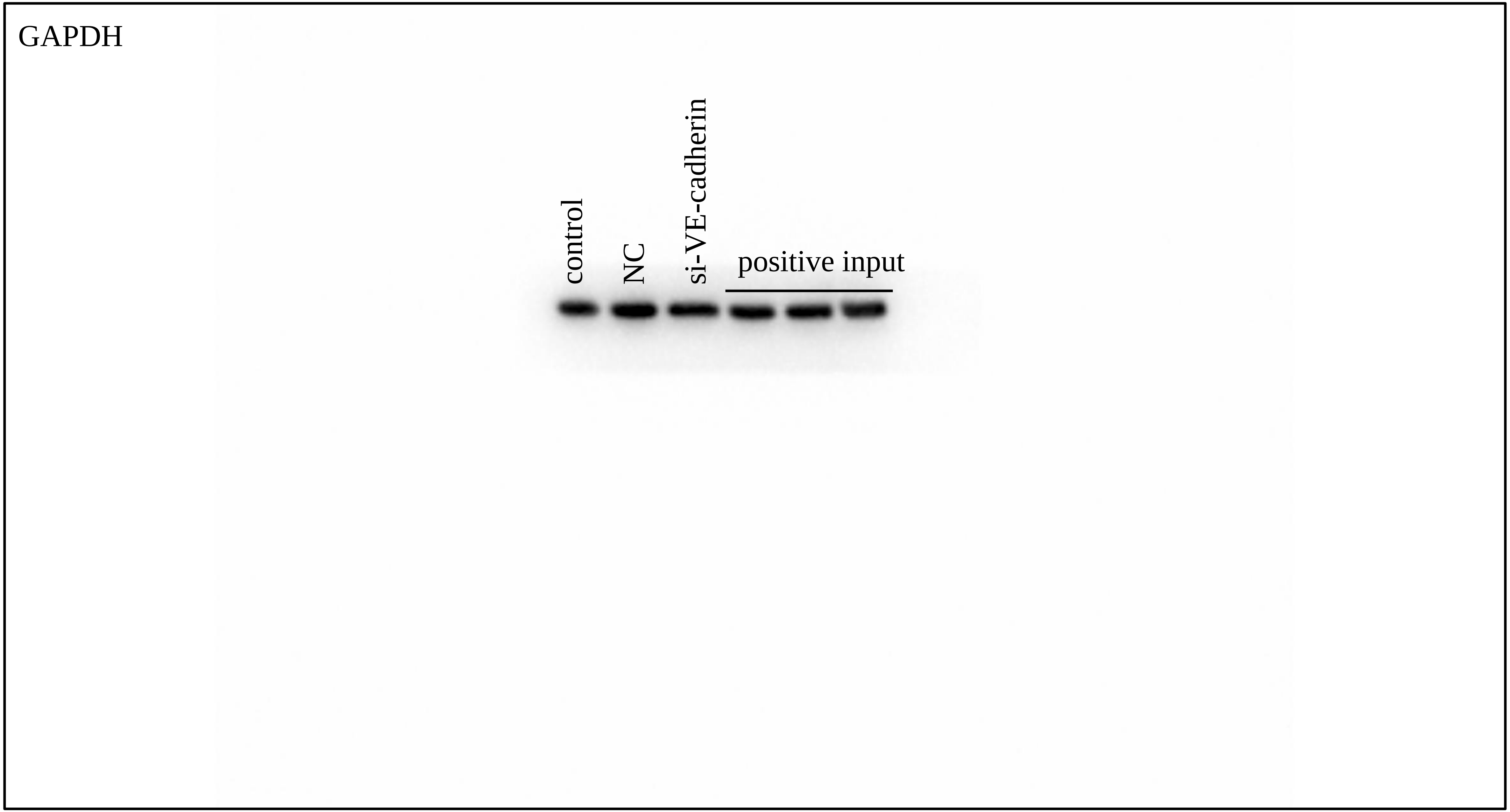

Figure S7 A-B Full scans of the entire original gels displayed in **Figure4 B**

Figure S7 A
Screenshot of gating strategies for flowcytometry shown in **Figure1A**

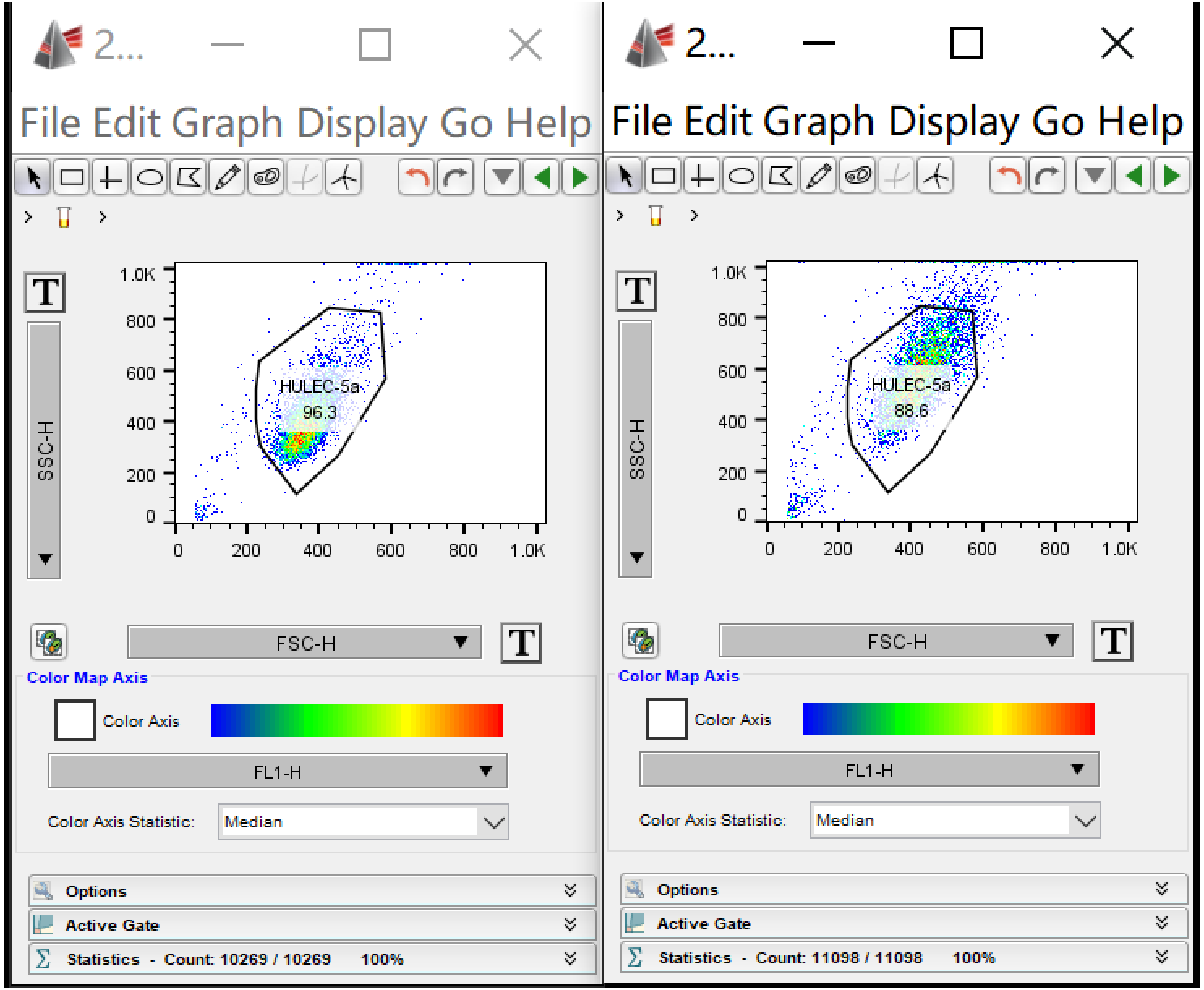

Figure S7 B
Screenshot of gating strategies for flowcytometry shown in **Figure1B**

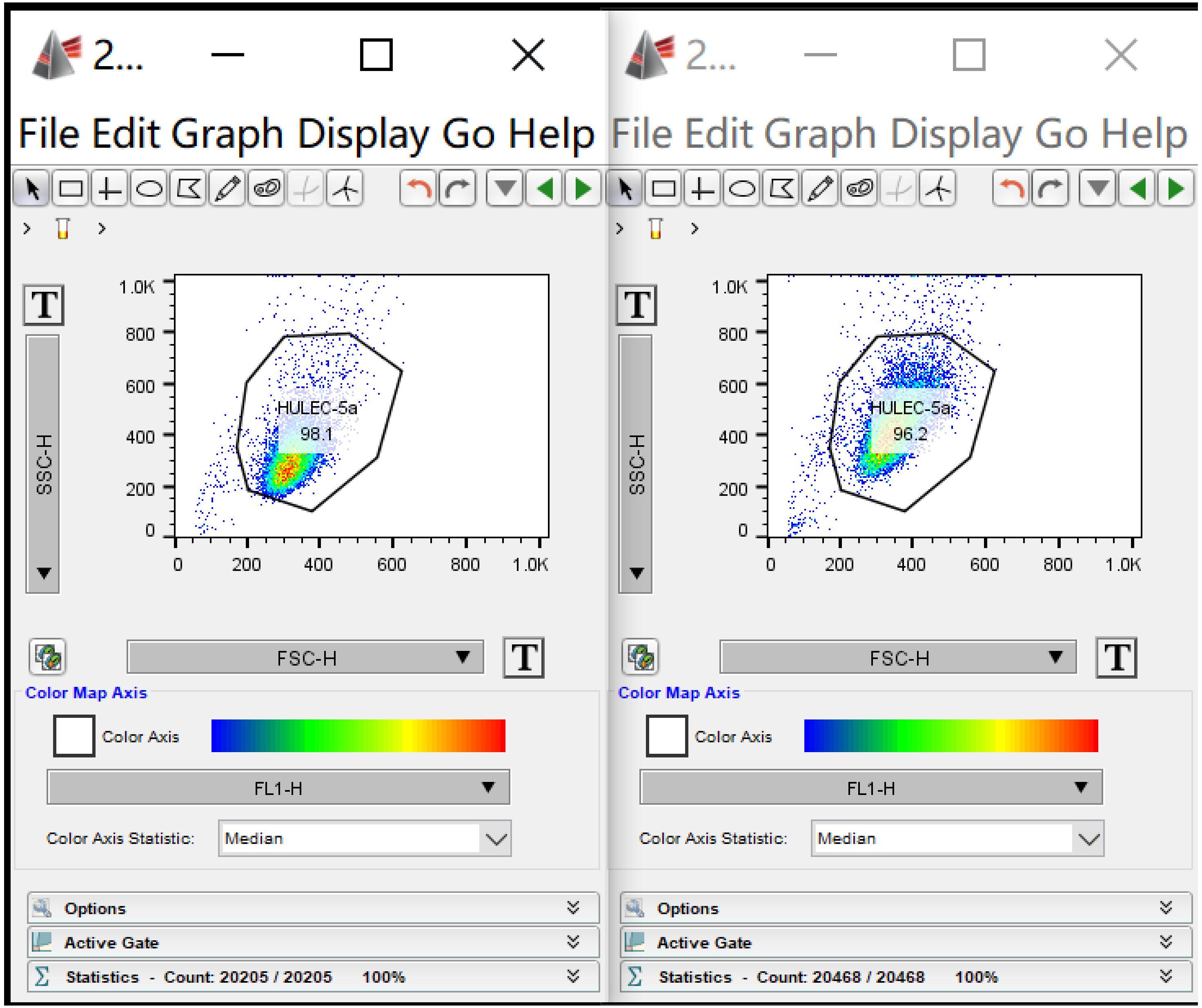

Figure S7 C

Screenshot of gating strategies for flowcytometry shown in **Figure2 A**

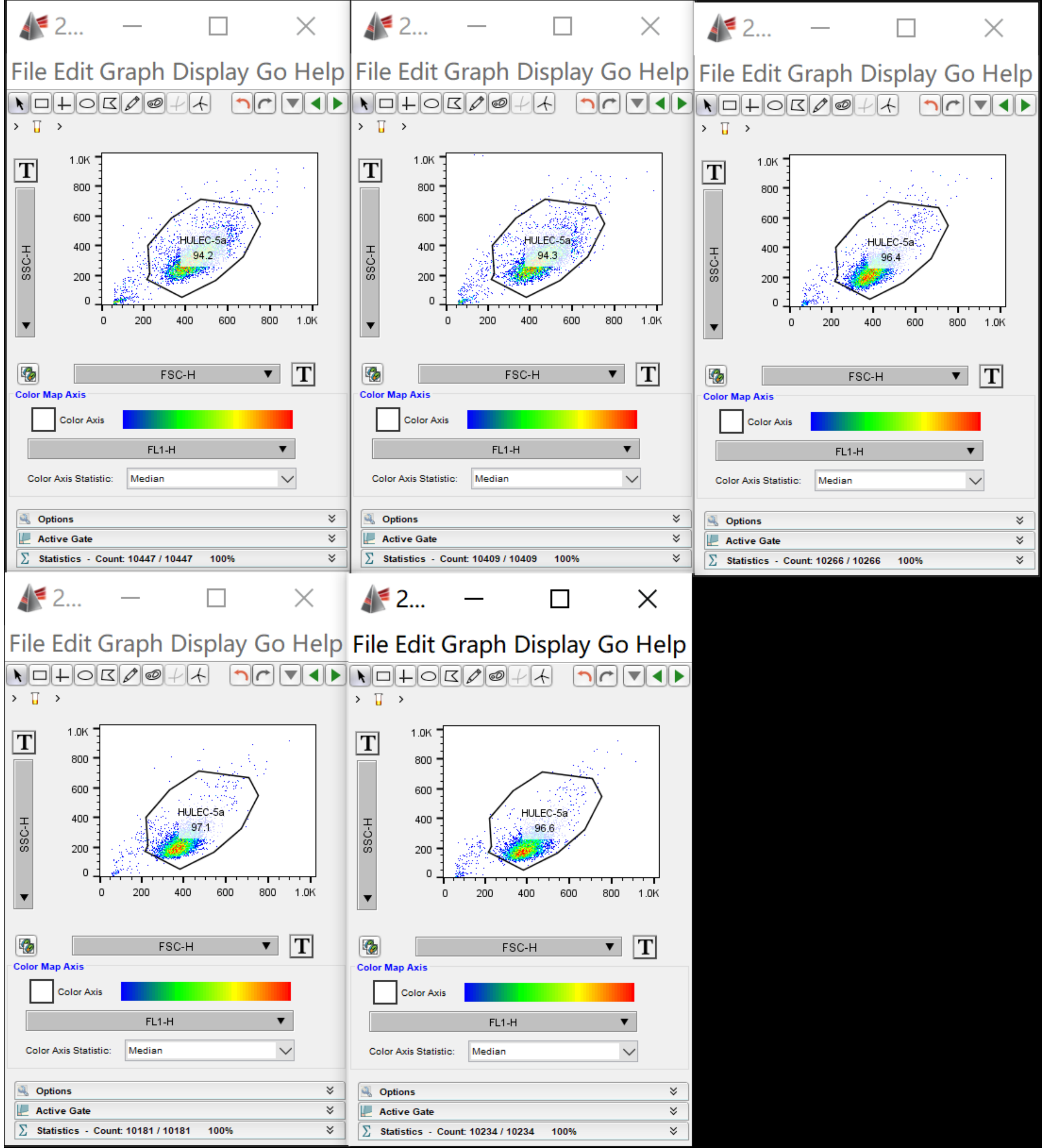

Figure S7 D
Screenshot of gating strategies for flowcytometry shown in **Figure2 B**

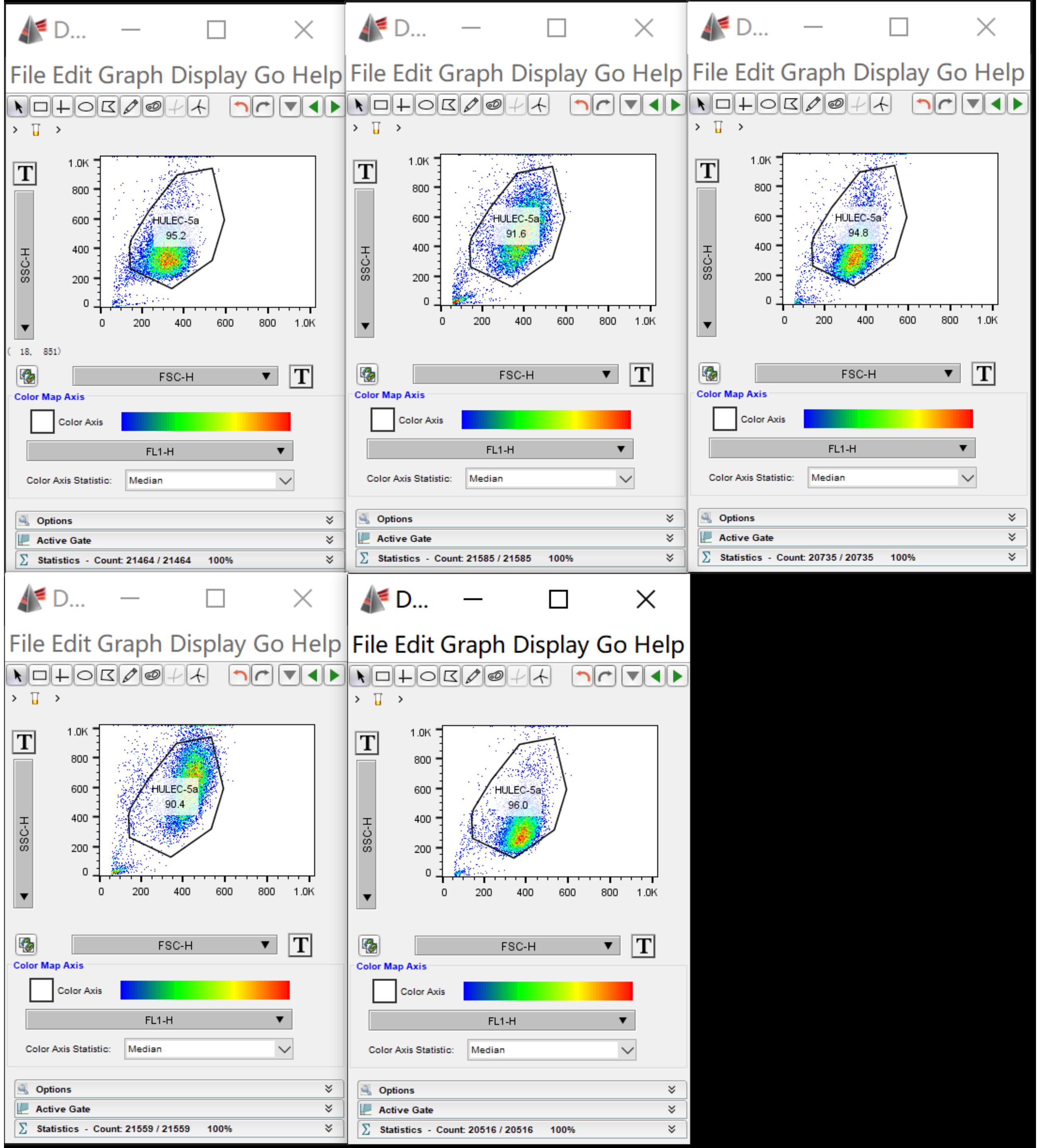

Figure S7 E Screenshot of gating strategies for flowcytometry shown in **Figure3 D.F**

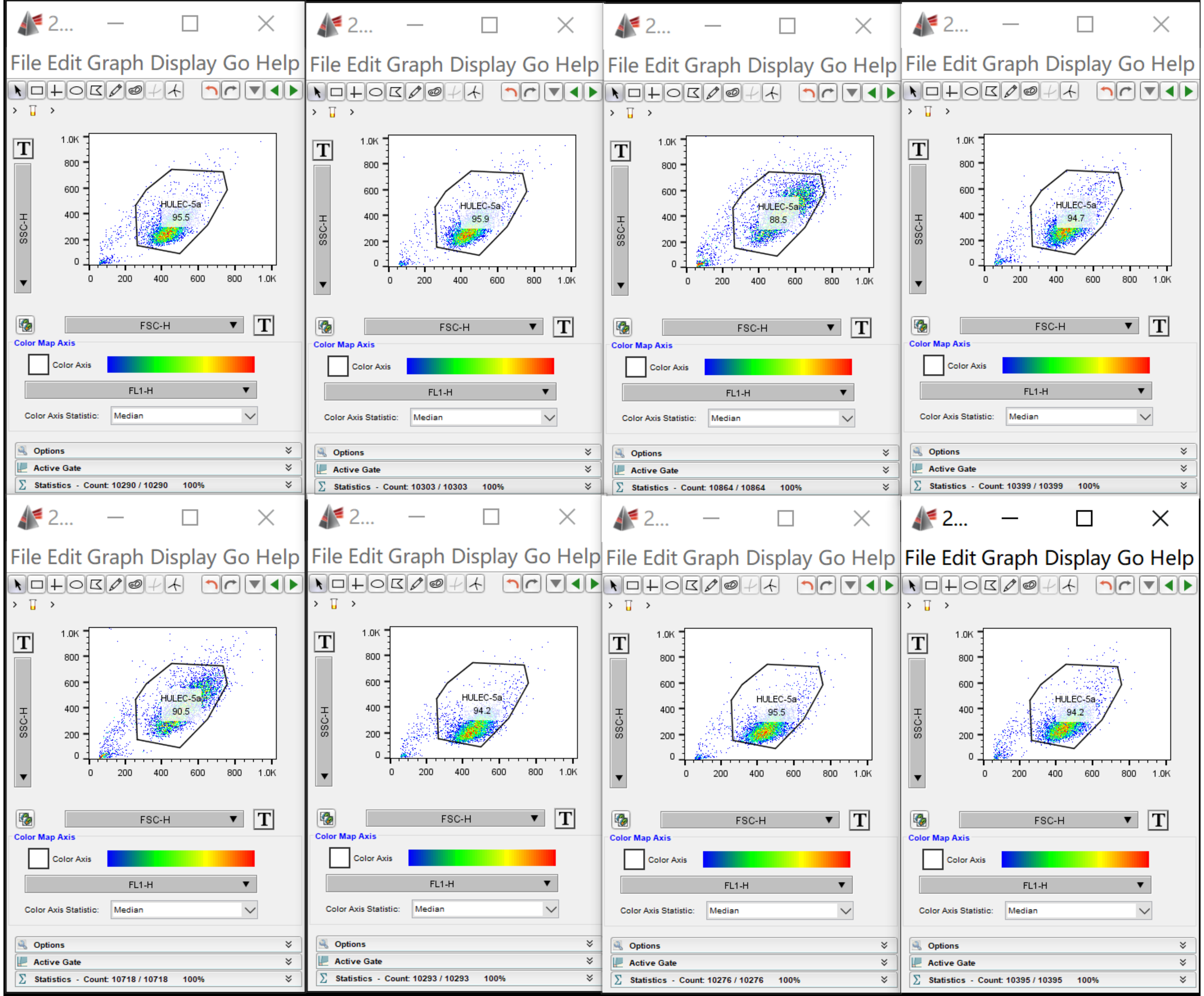

Figure S7 F
Screenshot of gating strategies for flowcytometry shown in **Figure3 E.G**

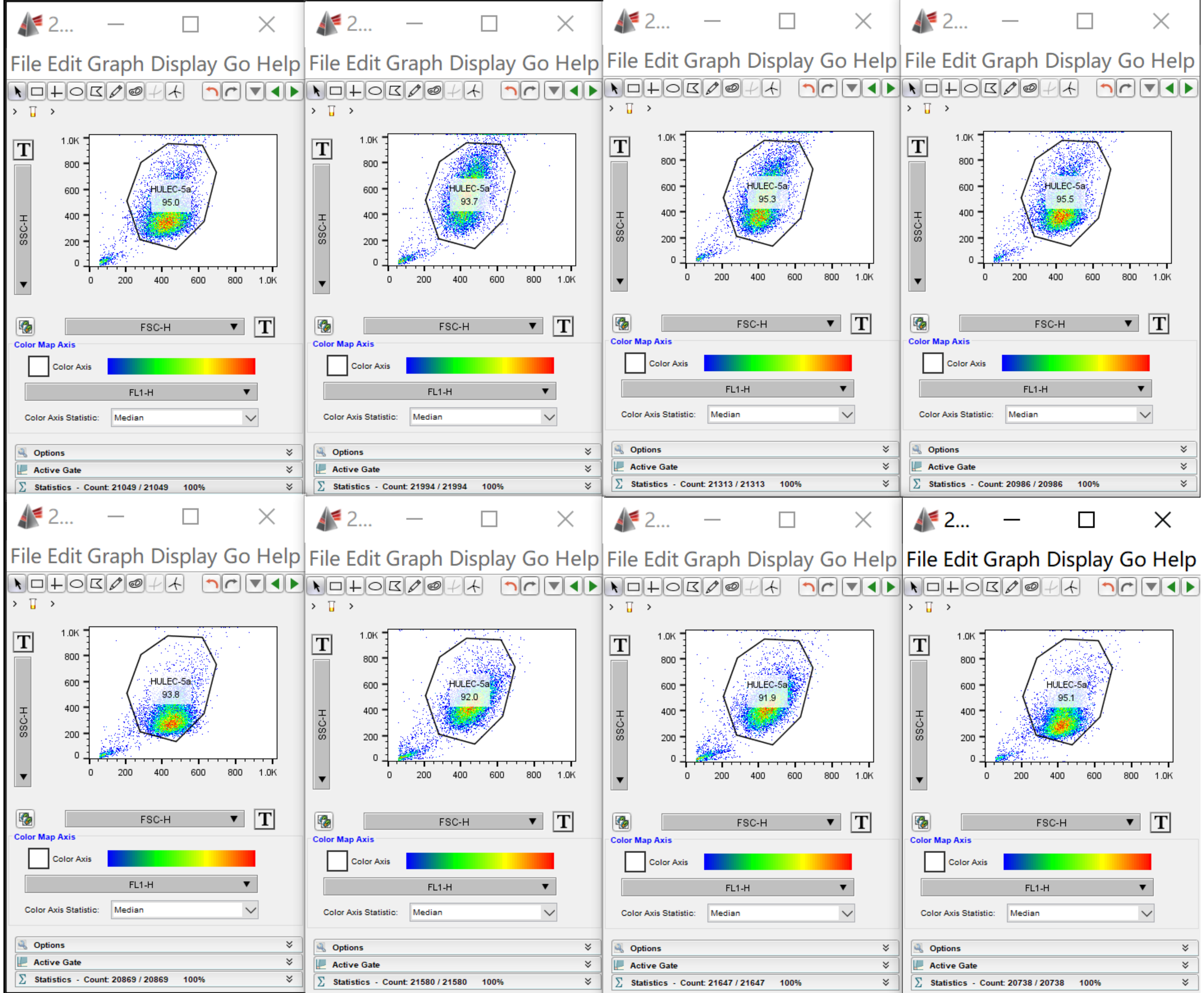

Figure S7 G
Screenshot of gating strategies for flowcytometry shown in **Figure4 E.H**

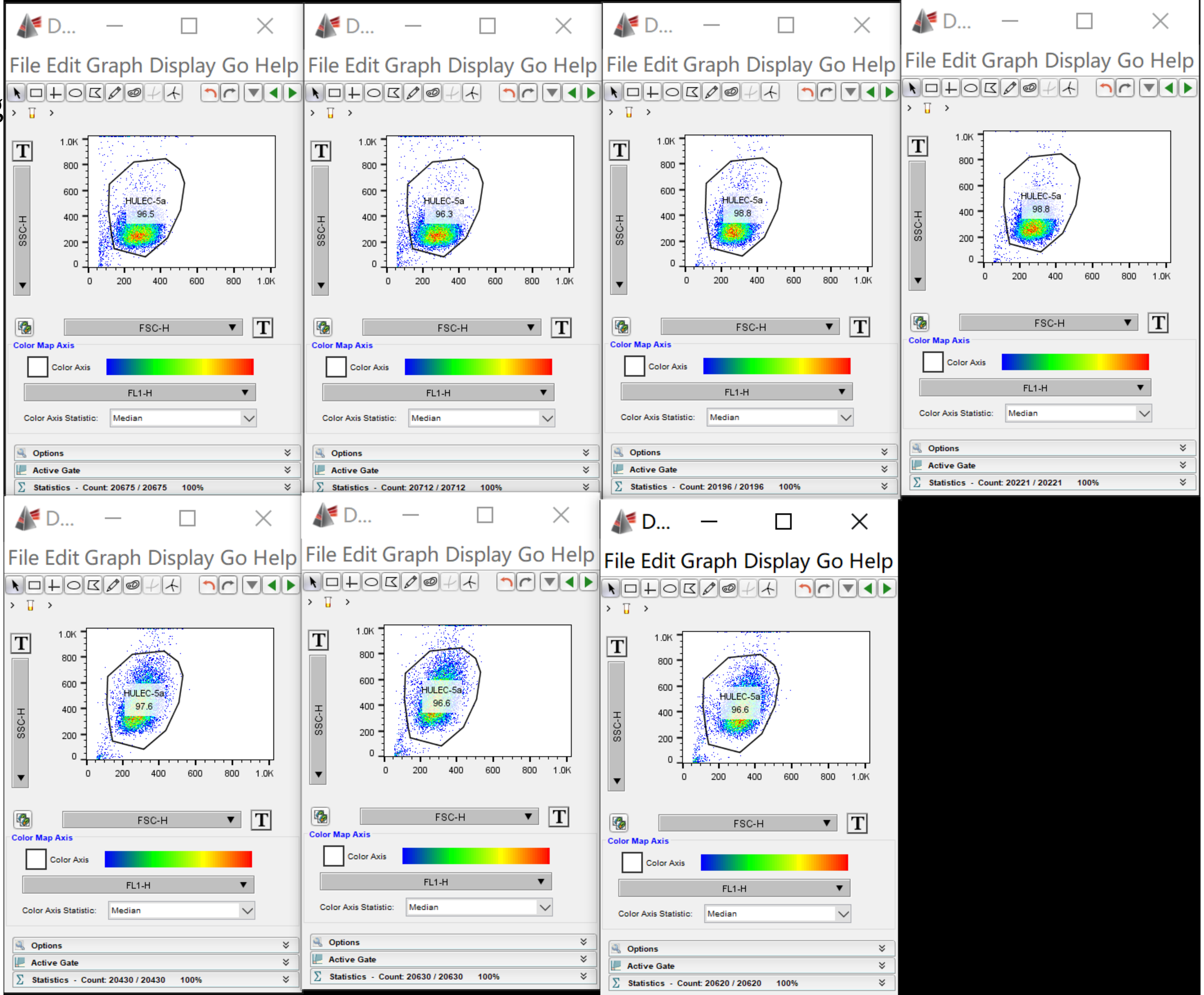

Figure S7 H
Screenshot of gating strategies for flowcytometry shown in **Figure4 F.I**

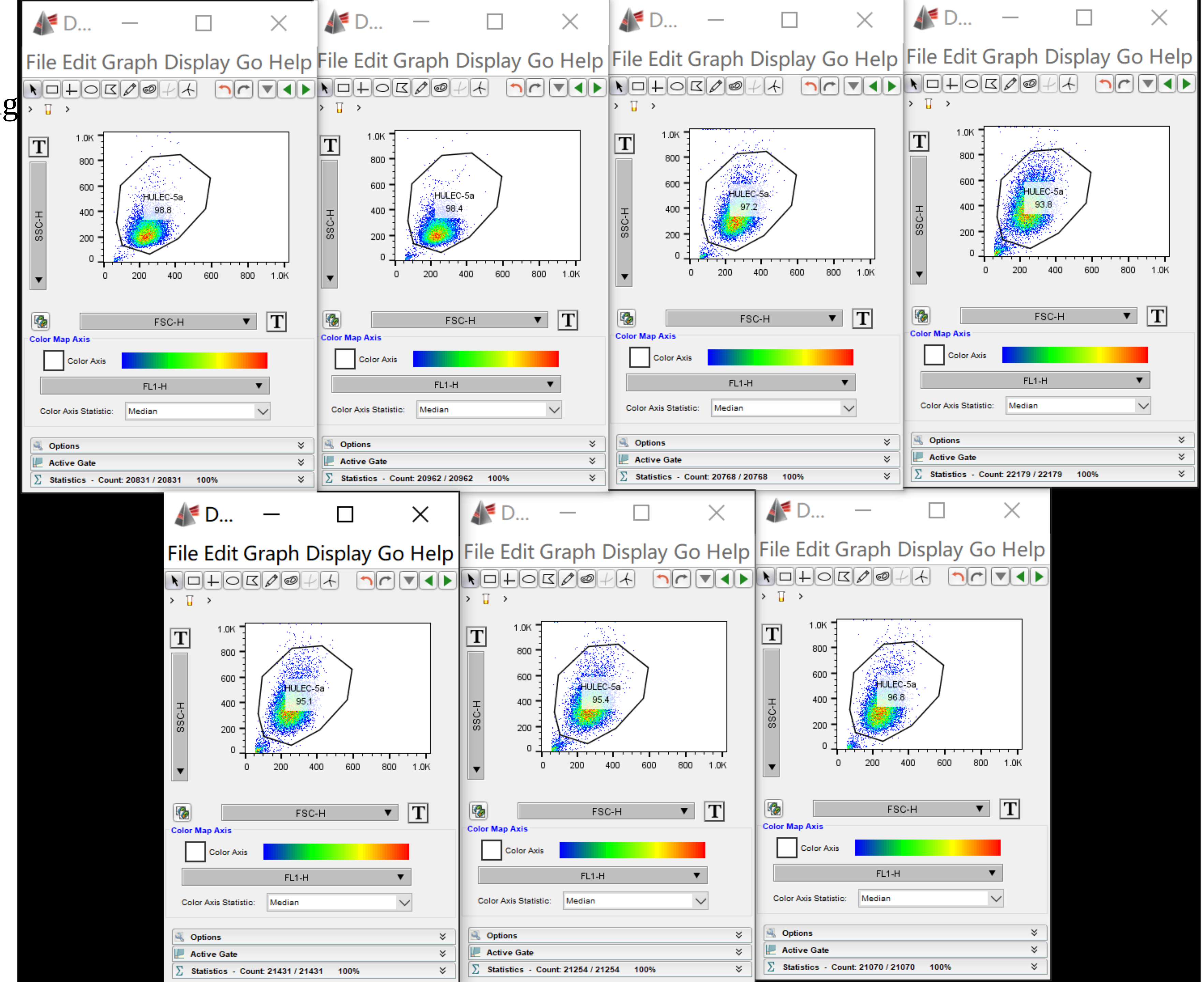

Supplement: Supplementary file 4 [file DataSheet1.PDF]
